# Systematic review of the relationships between sedentary behaviour and health indicators in the early years (0–4 years)

**DOI:** 10.1186/s12889-017-4849-8

**Published:** 2017-11-20

**Authors:** Veronica J. Poitras, Casey E. Gray, Xanne Janssen, Salome Aubert, Valerie Carson, Guy Faulkner, Gary S. Goldfield, John J. Reilly, Margaret Sampson, Mark S. Tremblay

**Affiliations:** 1 Healthy Active Living and Obesity Research Group, RI #1, Children’s Hospital of Eastern Ontario Research Institute, 401 Smyth Road, Ottawa, ON K1H 8L1 Canada; 2 University of Strathclyde, School of Psychological Science and Health, G1 1QE, Glasgow, Scotland UK; 3 Faculty of Physical Education and Recreation, University of Alberta, Edmonton, AB T6G 2H9 Canada; 4 School of Kinesiology, University of British Columbia, Vancouver, BC V6T 1Z3 Canada; 5 Library and Media Services, Children’s Hospital of Eastern Ontario, Ottawa, ON K1H 8L1 Canada

**Keywords:** Sedentary behaviour, Infants, Toddlers, Preschoolers, Early years, Screen time, Sitting, Reading, Adiposity, Motor development, Cognitive development, Bone and skeletal health, Cardiometabolic health, Fitness, Risks

## Abstract

**Background:**

The purpose of this systematic review was to examine the relationships between sedentary behaviour (SB) and health indicators in children aged 0 to 4 years, and to determine what doses of SB (i.e., duration, patterns [frequency, interruptions], and type) were associated with health indicators.

**Methods:**

Online databases were searched for peer-reviewed studies that met the a priori inclusion criteria: population (apparently healthy, 1 month to 4.99 years), intervention/exposure and comparator (durations, patterns, and types of SB), and outcome/health indicator (critical: adiposity, motor development, psychosocial health, cognitive development; important: bone and skeletal health, cardiometabolic health, fitness, risks/harm). The quality of the evidence was assessed by study design and outcome using the Grading of Recommendations Assessment, Development and Evaluation (GRADE) framework.

**Results:**

Due to heterogeneity, meta-analyses were not possible; instead, narrative syntheses were conducted, structured around the health indicator and type of SB. A total of 96 studies were included (195,430 participants from 33 countries). Study designs were: randomized controlled trial (*n* = 1), case-control (*n* = 3), longitudinal (*n* = 25), longitudinal with additional cross-sectional analyses (*n* = 5), and cross-sectional (*n* = 62). Evidence quality ranged from “very low” to “moderate”. Associations between objectively measured total sedentary time and indicators of adiposity and motor development were predominantly null. Associations between screen time and indicators of adiposity, motor or cognitive development, and psychosocial health were primarily unfavourable or null. Associations between reading/storytelling and indicators of cognitive development were favourable or null. Associations between time spent seated (e.g., in car seats or strollers) or in the supine position, and indicators of adiposity and motor development, were primarily unfavourable or null. Data were scarce for other outcomes.

**Conclusions:**

These findings continue to support the importance of minimizing screen time for disease prevention and health promotion in the early years, but also highlight the potential cognitive benefits of interactive non-screen-based sedentary behaviours such as reading and storytelling. Additional high-quality research using valid and reliable measures is needed to more definitively establish the relationships between durations, patterns, and types of SB and health indicators, and to provide insight into the appropriate dose of SB for optimal health in the early years.

**Electronic supplementary material:**

The online version of this article (10.1186/s12889-017-4849-8) contains supplementary material, which is available to authorized users.

## Background

Sedentary behaviour is defined as any waking behaviour with an energy expenditure of ≤1.5 metabolic equivalents (METs) while in a sitting or reclining posture [1]. It is increasingly recognized that too much sedentary behaviour can have negative health effects across the lifespan [2–4], which are distinct from those that result from low physical activity [5]. This may be of particular importance in the early years of life, given that these years are critical for growth and development, and that lifestyle behaviours established early in life tend to track over time [6–8].

In this regard, the *Canadian Sedentary Behaviour Guidelines for the Early Years (ages 0–4 years)* [9], and guidelines in other countries around the world (e.g., Australia [10] and USA [11]), recommend that children <2 years of age have no exposure to screens, and that those aged 2 to 4 years have <1 h/day of screen time. In addition, guidelines (e.g., in Canada [9], Australia [10], and the United Kingdom [12]) recommend that parents and caregivers minimize the time that children spend sitting or being restrained (e.g., in a stroller or high chair) while awake.

In contrast to these recommendations, ≥ 80% of young children are exposed to screens before the age of 2 years [13, 14], only 22% of Canadian children aged 3 to 4 years are meeting the screen time guidelines of <1 h/day, and on average parent-reported screen time for this age group is 2.0 h/day [15]. Moreover, young children are spending a substantial proportion of their time sedentary, and no guidance regarding an “appropriate” amount of total sedentary time exists. This is a notable gap, given that a recent review including data from 10 countries reported that children aged 2 to 5 years were sedentary for 34% to 94% of the day [16]. For instance, objectively measured data from a large, nationally representative sample of Canadian children showed that, on average, 3- to 4-year-olds were sedentary for 436 min/day (7 h, 16 min), which was roughly equivalent to 60% of their waking time [15].

The *Canadian Sedentary Behaviour Guidelines* were informed by a systematic review of the evidence that found that high levels of television (TV) time were associated with increased adiposity and reduced psychosocial health and cognitive development [2]. However, there was no evidence of benefits or harms for any other type of sedentary behaviour, for total sedentary time, or for patterns (e.g., frequency, interruptions) of sedentary time. This may be in part because only intervention and longitudinal studies were included in this earlier review [2]. This is a critical limitation because in recent years there has been a dramatic shift in the media landscape (e.g., evolving technologies including smartphones and tablets) [17], and because different types of sedentary behaviour (e.g., reading, sitting, playing video games) [18, 19] and different patterns of sedentary behaviour [20] may have different health effects. Evidence from large cross-sectional studies (with samples representative of the general population), together with new studies published since the original review, may provide additional insight.

In the intervening years, new systematic reviews have been conducted to investigate the relationships between sedentary behaviour and particular health indicators. For instance, Hinkley et al. found that too little evidence existed to draw conclusions regarding associations between sedentary behaviours and psychosocial well-being [21], and Carson et al. identified that different types of sedentary behaviour may have different effects on cognitive development in the early years of life (e.g., screen time may be detrimental, and reading beneficial) [18]. These recent reviews present focused summaries; however, no previous review has provided a balanced consideration of different types of sedentary behaviour and a range of holistic health indicators across study designs. Accordingly, a comprehensive review of the literature was needed in order to: 1) understand the health effects of sedentary behaviour in the early years, 2) inform and update population-level recommendations, and 3) identify research gaps and guide the design of future research and/or assist in the translation of current research to practice.

Therefore, the purpose of this study was to perform a systematic review that examined the relationships between sedentary behaviour and health indicators in children in their early years (0 to 4 years). An additional aim was to determine what doses of sedentary behaviour (i.e., duration, patterns [frequency, interruptions], and type) were associated with health indicators.

## Methods

### Protocol and registration

This systematic review was registered with the International Prospective Register of Systematic Reviews (PROSPERO; Registration no. CRD42016035270; available from http://www.crd.york.ac.uk/PROSPERO/display_record.asp?ID=CRD42016035270), and was conducted and reported following the Preferred Reporting Items for Systematic Reviews and Meta-Analyses (PRISMA) statement [22].

### Eligibility criteria

The Population, Interventions, Comparisons, Outcomes, and Study design (PICOS) framework [23] was used to identify key study concepts in the research question, and to facilitate the search process.

#### Population

The population of interest was apparently healthy children (i.e., general populations, including those with overweight and obesity; samples of clinical populations were ineligible) with a mean age of 1 month to 4.99 years (or, if no mean age was reported, samples described as: infants, toddlers, preschoolers, pre-elementary or pre-primary school age) for at least one sedentary behaviour measurement point. Subgroups were defined as follows: infants, 1 month to 1 year; toddlers, 1.1 to 3.0 years; and preschoolers, 3.1 to 4.99 years.

#### Intervention (exposure)

The intervention/exposure was a specific measure of sedentary behaviour (e.g., TV viewing, video gaming, iPad/tablet/touch-screen, smart phone, reading, puzzles, bouts, breaks, sedentary time, and “screen time” – defined as composite measures of screen use) obtained via objective (e.g., accelerometry) or subjective (e.g., proxy-report) methods. For infants, sedentary behaviour was operationally defined as any waking behaviour characterized by low energy expenditure (i.e., non-purposefully active) while restrained (e.g., in a stroller/pram, high chair, car seat/capsule), or when sedate (e.g., lying/sitting in a chair with little movement but not restrained). Time spent in the prone position (“tummy time”) was not considered sedentary behaviour because this is deemed “physical activity” in this age group. For toddlers and preschoolers, sedentary behaviour was defined as any waking behaviour characterized by an energy expenditure of ≤1.5 METs while in a sitting or reclining posture [1]. Studies defining sedentary behaviour as “physical inactivity” or “failing to meet physical activity guidelines” were excluded, because these definitions do not differentiate between sedentary behaviour and light-intensity physical activity. Studies of active video gaming exposures (e.g., Nintendo Wii™, Microsoft Kinect™, Sony’s Playstation Move™) were excluded because these games may elicit energy expenditure > 1.5 METs [24], as were studies reporting background TV or screen access (e.g., TV is turned on, but not necessarily being watched by the child) because the child could be engaged in a non-sedentary behaviour. For experimental studies, interventions had to target sedentary behaviour exclusively and not multiple health behaviours (e.g., both sedentary behaviour and diet).

#### Comparison

Various durations, patterns (frequencies, interruptions), and types of sedentary behavior were used for comparison where available. A comparison or control group was not required.

#### Outcomes (health indicators)

Eight health indicators were chosen by expert consensus among a 22-member group with expertise in movement behaviours in children. The health indicators were selected given consideration of the literature (previous reviews; e.g., [2]) and of the importance of including a range of holistic health indicators (i.e., physical, psychological/social, and cognitive health). Four health indicators were identified as *critical* (primary) by expert consensus: (1) adiposity (e.g., % body fat, weight status, waist circumference); (2) motor development (e.g., developmental milestones, gross/fine motor skills, locomotor-object control); (3) psychosocial health (e.g., depressive/anxiety symptoms, prosocial behaviour, aggression, self-regulation); and (4) cognitive development (e.g., language development, attention, executive function). Four health indicators were identified as *important* (secondary) by expert consensus: (1) bone and skeletal health (e.g., bone mineral density, bone mineral content, skeletal area); (2) cardiometabolic health (e.g., blood pressure, insulin resistance, blood lipids); (3) fitness (cardiovascular, musculoskeletal); and (4) risks (injury)/harm (e.g., plagiocephaly, torticollis).

#### Study designs

All study designs were considered. For longitudinal studies, any follow-up length was allowed as long as there was at least one measure of sedentary behaviour between the ages of 1 month to 4.99 years. For logistic reasons, and to maximize generalizability, minimum sample size requirements were imposed [25]; randomized controlled trials (RCTs) and non-randomized intervention studies were required to have at least 15 participants in at least one intervention group, and observational studies were required to have a minimum sample size of 100 participants. Published peer-reviewed original manuscripts and in-press manuscripts, in English or French, were eligible for inclusion. Grey literature (except for registered clinical trials) and conference abstracts were excluded.

### Information sources and search strategy

The following databases were searched using the Ovid interface: MEDLINE (1946 to April 13, 2016), EMBASE (1980 to 2016 week 15), PsycINFO (1806 to April Week 1 2016), and CENTRAL (February 2016). PubMed was searched for any additional studies not yet indexed in MEDLINE (April 11, 2016). SPORTdiscus (1949 to April 14, 2016) and Communication Source (April 12, 2016) were searched using the EBSCOhost interface, and the Communications and Mass Media Collection was searched using Gale. The MEDLINE search strategy was created by a research librarian with expertise in systematic review searching and peer-reviewed by a second research librarian. The search was then adapted for other databases. No study design limits were applied, and searches were limited to English and French publications. Updates to all search strategies, limited to randomized controlled trials for logistical reasons, were performed on November 1, 2016, to capture any additional studies that had been published in the interim between the initial searches and the data synthesis. The search strategies are presented in Additional file 1. Trial registries were also searched (https://clinicaltrials.gov/ and http://www.who.int/ictrp/en/; October 11, 2016) for ongoing clinical trials, using search terms for the sedentary behaviour concept and age group of interest. The International Journal of Child-Computer Interaction was hand-searched, because this journal was not yet indexed in any of these databases.

Bibliographic records were extracted as text files from the Ovid, EBSCOHost, and Gale interfaces and imported into Reference Manager Software (Version 11; Thompson Reuters, San Francisco, CA, USA), where duplicate records were removed. Titles and abstracts of the remaining records were uploaded to DistillerSR (Evidence Partners, Ottawa, ON, Canada), a secure internet-based software, where they were screened against inclusion criteria independently by two reviewers. Exclusion by both reviewers was required for a study to be excluded at the title and abstract stage; all other studies passed to full-text article screening. Two independent reviewers examined all full-text articles, and consensus was required for article inclusion in the review. Discrepancies between reviewers were resolved by discussion between themselves, or with the larger review team if needed. Relevant review articles identified during screening were also procured, and their reference lists manually checked for studies potentially missed by the search.

### Data extraction

Data extraction forms were created by the study coordinators, and reviewed and piloted by the review team. Extraction was completed in Microsoft Excel by one reviewer and checked for accuracy by a second reviewer. Reviewers were not blinded to the authors or journals when extracting data. Information was extracted regarding important study characteristics (e.g., citation, study design, country, sample size, age, and sex of participants); exposure (i.e., sedentary behaviour characteristics [e.g., type, volume, duration, frequency, pattern, and measurement and/or description of sedentary behaviour intervention]); outcome/health indicators (e.g., measurement type); results (e.g., odds ratio, difference in means); and covariates included in the analyses (if applicable; e.g., diet, physical activity). If data were unavailable for extraction (e.g., reported only in a graph, or described as “data not shown”), the authors were contacted. If data were presented subdivided by sex, the data were extracted independently for each sex only if data pooled across sex were unavailable. If analyses were reported for any other subsets of data, results were extracted for only the analyses using the full sample. The results from finally adjusted models were extracted when studies presented multiple models. Study findings were considered statistically significant at *p* < 0.05.

### Risk of bias and study quality assessment

The risk of bias was systematically evaluated in each primary research study using the methods described in the Cochrane Handbook [26]. All individual studies were assessed for the following potential sources of bias: selection bias, performance bias, detection bias, attrition bias, reporting bias, and other sources of bias (see Poitras et al. [25] for details).

The quality of evidence for each health indicator by each type of study design was assessed using the Grading of Recommendations, Assessment, Development and Evaluation (GRADE) framework [27]. The “quality of evidence” is the level of confidence in the estimate of effect. As such, the higher the quality of the evidence, the greater the confidence in the findings, and the lower the quality, the more likely it is that future research will change the level of confidence in the estimates and change the estimates themselves. According to GRADE, there are four levels of quality (“high”, “moderate”, “low”, and “very low”); evidence quality ratings start at “high” for randomized studies and at “low” for all other studies. The quality of evidence is downgraded if there are limitations across studies due to serious risk of bias, inconsistency (e.g., unexplained heterogeneity in the direction of the effect), indirectness (e.g., differences between the population, intervention and/or outcomes in included studies and those of interest, such as a surrogate measure instead of a direct measure of an outcome), or imprecision (e.g., wide confidence intervals that lead to uncertainty about the true magnitude of the effect) [28]. If there is no reason to downgrade, the quality of evidence can be upgraded if there is a large effect size, there is a dose-response gradient, or an effect is detected in the presence of plausible confounders or other biases that would decrease an apparent treatment effect [29].

In the present review, the overall quality of evidence for each study design within each health indicator was evaluated by two independent reviewers and verified by the larger review team. The review team decided a priori not to downgrade for risk of bias if the only potential sources of bias identified were use of a convenience sample or lack of exposure/outcome blinding, as in previous movement behaviour systematic reviews [25, 30].

### Synthesis of results

Meta-analyses were planned if data were sufficiently homogeneous in terms of statistical, clinical, and methodological characteristics. If meta-analyses were not possible, qualitative syntheses structured around the health indicator and type of sedentary behaviour were conducted, with all studies weighted equally, and the results presented narratively. Results were presented in “evidence profile” tables by outcome (health indicator) as per the GRADE framework (see Guyatt et al. [27] for details). For the purposes of this review, sedentary behaviours were grouped into three categories: 1) objectively measured sedentary time, 2) screen-based sedentary behaviours, and 3) other sedentary behaviours (e.g., reading, storytelling).

## Results

### Description of studies

**Fig. 1:**
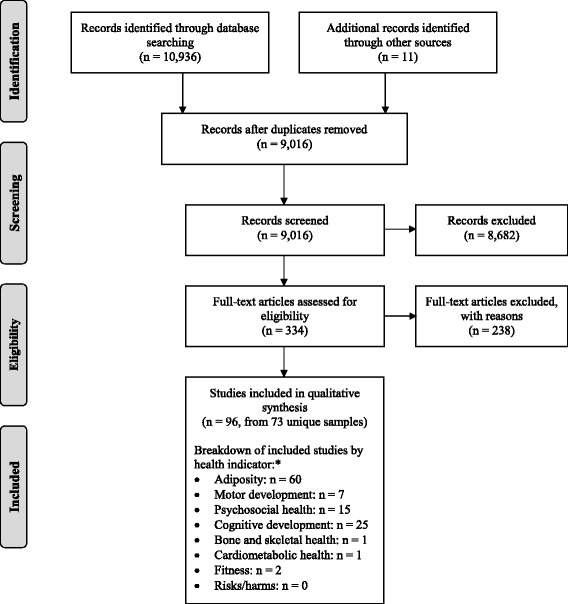
PRISMA flow diagram for the identification, screening, eligibility, and inclusion of studies. *Note that the numbers for each health indicator do not sum to the total number of included studies because more than one health indicator was reported in some studies

A total of 10,830 records were identified in the initial searches, and an additional 11 were identified by checking the reference lists of review articles (Fig. 1). After de-duplication, 8915 records remained. In the search update, an additional 106 records were identified (making a total of 10,936), and 101 of these remained after de-duplication. No relevant records were identified in the Trial Registry searches. After screening the 9016 titles and abstracts (from the initial and updated searches), 334 full-text articles were obtained for further review. Reasons for exclusion were: not in English or French language (*n* = 1), review paper (*n* = 2), sedentary behaviour included only as a covariate or outcome and not as the exposure (n = 2), sedentary behaviour defined as “failing to meet physical activity guidelines” (n = 2), sedentary behaviour exposure included background screens (*n* = 3), intervention did not target sedentary behaviour specifically/exclusively (*n* = 9), not original research (n = 9), no sedentary behaviour exposure (n = 9), sample size (*n* = 15), did not assess the relationship between sedentary behaviour and a relevant health indicator (*n* = 77), participants were not within appropriate age range (*n* = 92), and other (*n* = 17; e.g., comparator was the same “dose” of sedentary behaviour with different content, predatory publisher and problems with data such as incongruent values in text and tables). Some studies were excluded for multiple reasons. A total of 96 studies (from 73 unique samples) met the inclusion criteria (Fig. 1).

Detailed findings for the individual 96 studies are presented in Additional file 2: Tables S1-S7 and summarized in Tables 1, 2, 3, 4, 5, 6, 7 and 8. Data across studies involved 195,430 participants (147,752 from 73 unique samples), ranging from 103 [31] to 50,589 [32] participants. Participants from one study were not included in this sample size calculation because the sample size for the age group of interest was not reported [33]. Studies were conducted in 33 different countries, but were most commonly conducted in the United States (*n* = 44), Belgium (*n* = 7), Canada (n = 7), Australia (*n* = 6), Germany (*n* = 5), and the Netherlands (n = 5), with four or fewer studies from all other countries (Additional file 2: Tables S1-S7). The approximate baseline age ranged from 0.3 to 4.95 years. One study used an experimental design (randomized controlled trial); the remaining 95 studies used observational designs, including case-control (*n* = 3), longitudinal (*n* = 25), longitudinal with additional cross-sectional analyses (n = 5), and cross-sectional (*n* = 62).

**Table 1:** The relationship between sedentary behaviour and adiposity

No. of participants (No. of studies)	Design	Quality assessment					Absolute effect	Quality
		Risk of bias	Inconsistency	Indirectness	Imprecision	Other		
The range of mean ages at time of exposure measurement was ~0.75 to 4.95 years; the oldest mean age at follow-up was 15.5 years. Data were collected by randomized trial, case-control, cross-sectionally, and up to 12 years of follow-up. Adiposity measures were: BMI (absolute, z-score, SD score, percentile); fat mass index, lean mass index, trunk fat mass index; % body fat (measured using DXA); skinfold ratio (triceps skinfold thickness to subscapular skinfold thickness); sum of skinfolds; waist-to-height ratio; waist-to-hip ratio; weight-for-height (z-score); weight-for-age (z-score); waist circumference (absolute, z-score for age); weight status (CDC, IOTF, or WHO cut-points; Flemish reference data; French reference standards; Rolland Cachera reference curves; United Kingdom reference standards in 1999); total fat mass (SD score); lean mass (SD score).								
412 (1)	Randomized trial^a^	Serious risk of bias^b^	No serious inconsistency	No serious indirectness	No serious imprecision	None	**Screen time** ^c^ was significantly lower in the intervention vs control group at 2, 6, and 9 months post-intervention^d^. **BMI z-scores** were not different between the intervention and control groups at baseline or 9-month follow-up, but **BMI z-scores** increased in both groups [34].	Moderate^e^
32,699 (13)	Longitudinal^f^	Serious risk of bias^g^	No serious inconsistency	No serious indirectness	No serious imprecision	None	**Screen-based sedentary behaviours:** *Computer (duration):* **1/1 studies** reported null associations [85] *Computer games (frequency):* **1/1 studies** reported null associations [82] *Screen time (duration):* **2/3 studies** reported unfavourable associations [33, 84] **1/3 studies** reported null associations [87] *TV time (duration):* **6/10 studies** reported unfavourable associations [33, 54, 81, 83, 88, 90] **1/10 studies** reported null associations [86] **3/10 studies** reported mixed unfavourable and null associations [82, 85, 89] *Watching DVDs (duration):* **1/1 studies** reported unfavourable associations [83] **Other sedentary behaviours:** *Time in baby seats (duration):* **1/1 studies** reported mixed unfavourable, null, and favourable associations [45] *Time in the car (duration):* **2/2 studies** reported null associations [81, 82]	Very low^h^
1242 (2)	Case-control^i^	Serious risk of bias^j^	No serious inconsistency	No serious indirectness	No serious imprecision	None	**TV time** [35, 36] and **total sedentary time** [36] were not different between children with **overweight/obese** (case group) or **normal weight** (control group) status, but watching **TV for ≥ 1 h/day** was unfavourably associated with having **overweight status** (OR = 1.71, 95% CI: 1.07, 2.75, *p* = 0.02) [35].	Very low^k^
94,191 (47)	Cross-sectional^l^	Serious risk of bias^m^	No serious inconsistency	No serious indirectness	No serious imprecision	None	**Objectively measured sedentary time:** *Sedentary time 30-min bouts (accelerometer derived):* **1/1 studies** reported null associations [40] *Total sedentary time (accelerometer-derived):* **10/11 studies** reported null associations [37–40, 47, 53, 60, 75, 78, 80] **1/11 studies** reported mixed unfavourable and null associations [77] **Screen-based sedentary behaviours:** *Computer (duration):* **3/4 studies** reported null associations [63, 67, 79] **1/4 studies** reported mixed unfavourable and null associations [71] *Screen time (duration):* **6/18 studies** reported unfavourable associations [32, 33, 46, 50, 59, 73] **10/18 studies** reported null associations [44, 52, 57, 58, 62, 64, 65, 71, 72, 79] **2/18 studies** reported mixed unfavourable and null associations [41, 61] *TV time (duration):* **5/23 studies** reported unfavourable associations [33, 55, 66, 67, 71] **11/23 studies** reported null associations [31, 42, 43, 49, 50, 56, 60, 63, 69, 75, 76] **5/23 studies** reported mixed unfavourable and null associations [48, 51, 54, 68, 79] **1/23 studies** reported mixed null and favourable associations [74] **1/23 studies** reported mixed unfavourable, null, and favourable associations [70] *Using the internet (duration):* **1/1 studies** reported null associations [69] *Video games (duration):* **1/1 studies** reported unfavourable associations [69] *Watching DVDs/videos (duration):* **1/1 studies** reported null associations [69] **Other sedentary behaviours:** *Sedentary quiet play (duration):* **1/1 studies** reported mixed unfavourable and null associations [79] *Time in baby seats (duration):* **1/1 studies** reported null associations [45] *Using books (duration):* **1/1 studies** reported null associations [69]	Very low^n^

*BMI* Body Mass Index, *CDC* Centers for Disease Control and Prevention, *DXA* dual-energy X-ray absorptiometry, *IOTF* International Obesity Task Force, *SD* standard deviation, *WHO* World Health Organization

^a^Includes **1 randomized controlled trial** [34]

^b^Serious risk of bias. Unclear if allocation was adequately concealed prior to group assignment; group allocation was adequately concealed from control, but not intervention group during the study; unclear if height and weight were directly measured or proxy-reported; baseline data were not reported, making it impossible to determine if baseline imbalances existed between groups [34]

^c^Screen time was significantly lower in the intervention vs control group at 2 mo, 6 mo, and 9 mo follow-up post-intervention (mean ± SD: 2 mo: 39.48 ± 16.36 vs 86.64 ± 21.63 min/day; 6 mo: 24.72 ± 4.45 vs 84.95 ± 14.77 min/day; 9 mo: 21.15 ± 6.12 vs 93.96 ± 18.84 min/day; all *p* < 0.001)

^d^Intervention: 3 printed materials and interactive CDs and one counselling call intended to decrease screen time; 8-week duration. Control: Usual care; unaware of counselling interventions

^e^The quality of evidence from the randomized trial was downgraded from “high” to “moderate” because of a serious risk of bias that diminished the level of confidence in the observed effects

^f^Includes **13 longitudinal studies** [33, 45, 54, 81–90] from **9 unique samples**. Pagani et al. [90] and Fitzpatrick et al. [89] reported data from the Quebec Longitudinal Study of Child Development; Reilly et al. [81] and Leary et al. [82] reported data from the Avon Longitudinal Study of Parents and Children (ALSPAC); Gooze et al. [84] and Flores and Lin [83] reported data from the Early Childhood Longitudinal Study-Birth Cohort (ECLS-B); and Fuller-Tyszkiewicz et al. [54] and Wheaton et al. [85] reported data from the Longitudinal Study of Australian Children (LSAC). Results are presented separately and participants are counted only once

^g^Serious risk of bias. Questionable validity and reliability of the exposure measure [33, 45, 54, 81–90]. Data were reported as missing, but amount and reasons were not provided [89]. Height and weight data were incomplete without explanation for 23% of the analyzed sample and 60.7% of the original cohort [81]. Possible selective reporting: differences between included and excluded participants were reported for confounding variables but not exposure variables without explanation [82]. BMI at age 3 yr was analyzed, but was not reported in the purpose or methods [88]. Did not account for potentially important confounding variables or mediating factors: sugar-sweetened beverage consumption and sleep were assessed but not accounted for [33]; diet was not measured or included in the analysis [45]; adjusted for physical activity [89]; of the potential child and family confounders that were assessed, potential confounders were included or omitted from analyses based on the authors’ determination of what was “likely to be linked to our predictor or outcome variables,” without providing a basis for that determination [89]. Data were pooled from the control and experimental groups of a messaging-based obesity prevention intervention study [33]

^h^The quality of evidence from the longitudinal studies was downgraded from “low” to “very low” because of a serious risk of bias that diminished the level of confidence in the observed effects

^i^Includes **2 case-control studies **[35, 36]

^j^Serious risk of bias. Questionable validity and reliability of the 1-day physical activity recall questionnaire [36]. Potentially inappropriate statistical analysis: investigators dichotomized participants by category of TV viewing of ≥1 h/day or <1 h/day based on exploratory bivariate analyses that showed 1 h to be the duration most related to children’s weight status [35]

^k^The quality of evidence from the case-control studies was downgraded from “low” to “very low” because of a serious risk of bias that diminished the level of confidence in the observed effects

^l^Includes **47 cross-sectional studies** [31–33, 37–80] from **40 unique samples**. Williams et al. [37], Byun et al. [39], and Byun et al. [38] reported data from the Children’s Activity and Movement in Preschool Study (CHAMPS); Sijtsma et al. [45] and Sijtsma et al. [46] reported data from the Groningen Expert Center for Kids with Obesity (GECKO) Drenthe birth cohort; Manios et al. [48], Kourlaba et al. [49], and van Stralen et al. [50] reported data from the Growth, Exercise and Nutrition Epidemiological Study in preSchoolers (GENESIS); Mendoza et al. [71] reported data from the National Health and Nutrition Examination Survey (NHANES) 1999 to 2002, Fulton et al. [72] from NHANES 1999 to 2006, and Twarog et al. [73] from NHANES 2008 to 2012; Taverno Ross et al. [76] and Espana-Romero et al. [77] reported data from the Study of Health and Activity in Preschool Environments (SHAPES); Brown et al. [55] and Fuller-Tyszkiewicz et al. [54] reported data from the Longitudinal Study of Australian Children (LSAC); Dolinsky et al. [53] and Boling Turer et al. [45] reported data from Kids and Adults Now: Defeat Obesity! (KAN-DO). Results are presented separately and participants are counted only once

^m^Serious risk of bias. Potentially inappropriate sampling technique: participants were a non-representative convenience sample [66]; sampling deviated from protocol and specific deviations were not documented [57]. Potentially inappropriate measurement tools were used: questionable validity and reliability of the exposure measure [31–33, 41, 43–46, 49–51, 54–62, 64–76] and outcome measure [65]; questionable validity of exposure measure [42, 52, 63, 79]; poor reliability of exposure measure [42]; height and weight were obtained by parent-report [44, 70]; options for 2–3 h and 4–5 h were missing from the Likert-type scale used to assess screen time [74]; applied accelerometry cut-points were not validated for the age group of interest [47]. Potential attrition bias: amount of unexplained missing exposure or outcome data is unknown [42, 50] or ranged from 14% to 67% [39, 40, 42, 43, 59, 60, 69, 71, 73, 74, 76], and reason may be related to the true outcome of interest [40, 43, 66, 71]. Potential selective reporting bias: statistics for non-significant relationships were not reported [48, 64]; authors decided post-hoc not to report analyses with continuous exposure variables [59]; only final model was reported [44]; results for correlations described in the methods section were not reported [62]; composite outcomes were presented without individual components; results for categorical screen time and total screen time described in the methods section were not reported [32]; outcomes from pooled hierarchical linear regression and variance information of included results were not reported [70]. Did not account for potentially important confounding variables or mediating factors: diet [43, 45, 46, 50, 58, 60, 63, 64, 67, 71, 72, 77, 80]; sugar-sweetened beverage consumption; and sleep [33]. Controlled for physical activity [59, 61, 66, 78]. Sleep during the day was considered sedentary time [40]

^n^The quality of evidence from the cross-sectional studies was downgraded from “low” to “very low” because of a serious risk of bias that diminished the level of confidence in the observed effects

**Table 2:** The relationship between sedentary behaviour and motor development

No. of participants (No. of studies)	Design	Quality assessment					Absolute effect	Quality
		Risk of bias	Inconsistency	Indirectness	Imprecision	Other		
Participant ages at time of exposure measurement ranged from ~4 mo (0.3 yr) to 3–4 years; the oldest mean age at follow-up was 5.4 years. Data were collected cross-sectionally and up to 3 years of follow-up. Motor development indicators were assessed by parent-report unless otherwise indicated; specific indicators were: age at first sitting, age at first crawling, age at first walking, locomotion/locomotor skills (assessed by a “test of gross motor development” or CHAMPS Motor Skill Protocol), motor skill development (assessed by the PDMS-2 or CHAMPS Motor Skill Protocol), motor skills (assessed by a “neurological optimality score”), object control (assessed by a “test of gross motor development”, or CHAMPS Motor Skill Protocol), and visual-motor abilities (assessed by the WRAVMA test).								
3413 (3)	Longitudinal^a^	Serious risk of bias^b^	No serious inconsistency	No serious indirectness	No serious imprecision	None	**Screen-based sedentary behaviours:** *TV time (duration):* **2/3 studies** reported null associations [88, 91] **1/3 studies** reported mixed unfavourable and null associations [92] **Other sedentary behaviours:** *Time in a baby carrier/sling (duration):* **1/1 studies** reported null associations [91] *Time in a car seat (duration):* **1/1 studies** reported mixed null and favourable associations [91] *Time in a high chair or other chair (duration):* **1/1 studies** reported null associations [91] *Time in a playpen (duration):* **1/1 studies** reported null associations [91] *Time in a stroller (duration):* **1/1 studies** reported null associations [91]	Very low^c^
681 (4)	Cross-sectional^d^	Serious risk of bias^e^	No serious inconsistency	No serious indirectness	No serious imprecision	None	**Objectively measured sedentary time:** *Sedentary time 30-min bouts (accelerometer-derived):* **1/1 studies** reported null associations [40] *Total sedentary time (accelerometer-derived):* **1/2 studies** reported null associations [40] **1/2 studies** reported mixed unfavourable and null associations [37] **Screen-based sedentary behaviours:** *TV time (duration)*: **1/1 studies** reported unfavourable associations [94] **Other sedentary behaviours:** *Time in supine position (duration):* **1/1 studies** reported mixed unfavourable and null associations [93]	Very low^f^

*CHAMPS* Children’s Activity and Movement in Preschool Study, *PDMS-2* Peabody Developmental Motor Scales–second edition, *WRAVMA* Wide-Range Assessment of Visual Motor Ability

^a^Includes **3 longitudinal studies** [88, 91, 92] from **3 unique samples**

^b^Serious risk of bias. Questionable validity and reliability of exposure measure [88, 91, 92]

^c^The quality of evidence from longitudinal studies was downgraded from “low” to “very low” because of a serious risk of bias that diminished the level of confidence in the observed effects

^d^Includes **4 cross-sectional studies** [37, 40, 93, 94] from **4 unique samples**

^e^Serious risk of bias. Questionable validity and reliability of exposure measure [93, 94]; large amount (30.9%) of unexplained missing data and pattern of nonresponse indicates reason for missing data may have been related to the outcome of interest [40]; sleep during the day was included in sedentary time exposure [40]

^f^The quality of evidence from cross-sectional studies was downgraded from “low” to “very low” because of a serious risk of bias that diminished the level of confidence in the observed effects

**Table 3:** The relationship between sedentary behaviour and psychosocial health

No. of participants (No. of studies)	Design	Quality assessment					Absolute effect	Quality
		Risk of bias	Inconsistency	Indirectness	Imprecision	Other		
The range of mean ages at time of exposure measurement was ~1 to 4.3 years; the oldest mean age at follow-up was ~12 years. Data were collected by randomized trial, cross-sectionally, and up to 9.5 years of follow-up. Psychosocial health measures were: aggression toward a sibling (assessed by the Aggressive Sibling Social Behavior Scale); aggressive behaviours/aggression, delinquent behaviours, total behaviour problems, externalizing problems, internalizing problems, emotional reactivity, anxious or depressed symptoms, and attention problems (assessed by the CBCL or Japanese CBCL); attentional problems (assessed by the hyperactivity subscale of the BPI); attention problems and hyperactivity (assessed by the BASC-2); bullying (assessed by unpublished questionnaire); co-operation, assertion, responsibility, self-control, and total social skills (assessed by the Social Skills Rating System); emotional symptoms/problems, conduct problems, hyperactivity-inattention, peer problems, and prosocial behaviour (assessed using the SDQ); self-esteem, emotional well-being, family functioning, and social networks (assessed using the KINDL^R^); social-emotional competence (assessed by the MIT-SEA); soothability, sociability, and emotionality (assessed by the CTQ); victimization, anxiety, physical aggression, and prosocial behaviour (assessed by the SBQ); and risk of being a bully, victim, or bully-victim (assessed by unpublished questionnaire).								
412 (1)	Randomized trial^a^	Serious risk of bias^b^	No serious inconsistency	No serious indirectness	No serious imprecision	None	**Screen time** ^c^ was significantly lower in the intervention vs control group at 2, 6, and 9 months post-intervention^d^. **Aggressive and delinquent** behaviours were not different between the intervention and control groups at baseline, but were significantly lower in the intervention vs control group at 9-months post-intervention [34].	Moderate^e^
13,301 (9)	Longitudinal^f^	Serious risk of bias^g^	No serious inconsistency	No serious indirectness	No serious imprecision	None	**Screen-based sedentary behaviours:** *Time e-gaming or on a computer (duration):* **1/1 studies** reported mixed unfavourable and null associations [96] *TV time (duration):* **2/9 studies** reported unfavourable associations [95, 103] **5/9 studies** reported mixed unfavourable and null associations [90, 92, 96, 97, 99] **1/9 studies** reported null associations [100] **1/9 studies** reported mixed null and favourable associations [102]	Very low^h^
9429 (7)	Cross-sectional^i^	Serious risk of bias^j^	No serious inconsistency	No serious indirectness	No serious imprecision	None	**Objectively measured sedentary time:** *Total sedentary time (accelerometer-derived):* **1/1 studies** reported null associations [104] **Screen-based sedentary behaviours:** *TV time (duration):* **2/6 studies** reported unfavourable associations [101, 103] **2/6 studies** reported null associations [100, 106] **1/6 studies** reported mixed unfavourable and null associations [105] **1/6 studies** reported mixed null and favourable associations [98]	Very low^k^

*BASC-2* Behavior Assessment System for Children, *BPI* Behavior Problems Index, *CBCL* Child Behavior Checklist, *CTQ* Child Temperament Questionnaire, *KINDL* ^*R*^ Questionnaire for Measuring Health-Related Quality of Life in Children and Adolescents-Revised Version, *MIT-SEA* Modified Infant-Toddler Social and Emotional Assessment, *SBQ* Social Behavior Questionnaire, *SDQ* Strengths and Difficulties Questionnaire

^a^Includes **1 randomized controlled trial** [34]

^b^Serious risk of bias. Unclear if allocation was adequately concealed prior to group assignment; group allocation was adequately concealed from control, but not intervention group during the study; knowledge of outcome of interest was not prevented and outcome measurement is likely to have been influenced by lack of blinding; baseline data were not reported, making it impossible to determine if baseline imbalances existed between groups [34]

^c^Screen time was significantly lower in the intervention vs control group at 2-, 6-, and 9-month follow-up post-intervention (mean ± SD: 2 month: 39.48 ± 16.36 vs 86.64 ± 21.63 min/day; 6 month: 24.72 ± 4.45 vs 84.95 ± 14.77 min/day; 9 month: 21.15 ± 6.12 vs 93.96 ± 18.84 min/day; all *p* < 0.001)

^d^Intervention: 3 printed materials and interactive CDs and one counselling call, intending to decrease screen time; 8-week duration. Control: Usual care; unaware of counselling interventions

^e^The quality of evidence from the randomized trial was downgraded from “high” to “moderate” because of a serious risk of bias in the single randomized controlled trial that diminished the level of confidence in the observed effects

^f^Includes **9 longitudinal studies** [90, 92, 95–97, 99, 100, 102, 103] from **6 unique samples**. Verlinden et al. [97, 99] reported data from the Generation R Study; and Pagani et al. [90, 92] and Watt et al. [95] reported data from the Quebec Longitudinal Study of Child Development (QLSCD). Results are presented separately and participants are counted only once

^g^Serious risk of bias. Questionable validity and reliability of television duration exposure measure [90, 92, 97, 99, 100, 102, 103]; questionable validity and reliability of television duration exposure measure on weekdays only [96]; poor reliability of outcome measures for responsibility [102] and emotional symptoms, conduct problems, peer problems, and prosocial behaviour [100]; large amount of unexplained missing data and pattern of nonresponse indicates reason for missing data may have been related to the outcome of interest [97]; complete results were not reported for all relationships examined [99]

^h^The quality of evidence from longitudinal studies was downgraded from “low” to “very low” because of a serious risk of bias that diminished the level of confidence in the observed effects

^i^Includes **7 cross-sectional studies** [98, 100, 101, 103–106] from **7 unique samples**

^j^Serious risk of bias. Questionable validity and reliability of television duration exposure measure [98, 100, 101, 103, 105, 106]; poor reliability of outcome measures for emotional symptoms, conduct problems, peer problems, and prosocial behaviour [100]; small amount (218/4020) of unexplained missing outcome data at 3-year follow-up [92]

^k^The quality of evidence from cross-sectional studies was downgraded from “low” to “very low” because of a serious risk of bias that diminished the level of confidence in the observed effects

**Table 4:** The relationship between sedentary behaviour and cognitive development

No. of participants (No. of studies)	Design	Quality assessment					Absolute effect	Quality
		Risk of bias	Inconsistency	Indirectness	Imprecision	Other		
The range of mean ages at time of exposure measurement was ~0.5 to 4.4 years; the oldest age range at follow-up was 9 to 10 years. Data were collected cross-sectionally and up to 8 years of follow-up. Cognitive development indicators were: ADHD symptoms (assessed by checklists based on the DSM-IV); attentional problems (assessed by the BPI); attention span (assessed by the CTQ); classroom engagement (assessed by a Classroom Engagement Scale and an unpublished questionnaire); cognitive ability (assessed by the Imitation Sorting Task); cognitive development (assessed by BSID-II and BSID-III); cognitive inhibitory control (assessed by the Animal Stroop Task); executive function (assessed as a composite of cognitive inhibitory control and working memory capacity; the BASC-2; four tasks: grass/snow, whisper, backward digit span, tower); language development (total), auditory comprehension, expressive communication (assessed by ASQ, PLS-4, CELF-P2, CELF-4, CDI, K-ASQ, Thai CLAMS, medical diagnosis, and developmental assessment with Denver-II test); mathematical success (assessed as relative to the class distribution); mathematics, reading recognition, reading comprehension (assessed by the PIAT); number knowledge (assessed by NKT); receptive and total vocabulary (assessed by PPVT); short-term memory (assessed by the Memory for Digit Span of the WISC); speech disorders (assessed by the Chuturik test and Child Behavior Checklist by Achenbach, conversation with parents, and clinical examination); and working memory capacity (assessed using the Animal Stroop Task and K-ABC number recall test).								
8927 (11)	Longitudinal^a^	Serious risk of bias^b^	No serious inconsistency	No serious indirectness	No serious imprecision	None	**Screen-based sedentary behaviours:** *Electronic media exposure (duration):* **1/1 studies** reported unfavourable associations [112] **Other sedentary behaviours:** *Parents reading (frequency):* **1/1 studies** reported favourable associations [121] *TV time (duration):* **5/10 studies** reported unfavourable associations [90, 92, 100, 120, 121] **4/10 studies** reported null associations [88, 102, 113, 122] **1/10 studies** reported mixed unfavourable, null, and favourable associations [119]	Very low^c^
166 (1)	Case-control^d^	Serious risk of bias^e^	No serious inconsistency	No serious indirectness	No serious imprecision	None	**Screen-based sedentary behaviours:** *TV time:* **1/1 studies** reported unfavourable associations [116]	Very low^f^
9330 (16)	Cross-sectional^g^	Serious risk of bias^h^	No serious inconsistency	No serious indirectness	No serious imprecision	None	**Objectively measured sedentary time:** *Total sedentary time (accelerometer-derived):* **1/1 studies** reported null associations [104] **Screen-based sedentary behaviours:** *Computer use (yes, no):* **1/1 studies** reported null associations [109] *Mobile phone use (yes, no):* **1/1 studies** reported unfavourable associations [109] *TV time (duration):* **3/9 studies** reported unfavourable associations [94, 108, 123] **4/9 studies** reported null associations [90, 100, 114, 115, 121] **1/9 studies** reported mixed unfavourable and null associations [118] *Total media exposure (duration):* **1/1 studies** reported mixed null and unfavourable associations [124] *Video games (duration):* **1/1 studies** reported null associations [107] **Other sedentary behaviours:** *Reading with parents (duration, frequency):* **1/3 studies** reported null associations [110] **1/3 studies** reported favourable associations [117] **1/3 studies** reported mixed null and favourable associations [124] *Screen time (duration):* **1/1 studies** reported unfavourable associations [111] *Storytelling with parents (frequency):* **2/2 studies** reported mixed null and favourable associations [117, 124]	Very low^i^

*ADHD* Attention-Deficit/Hyperactivity Disorder, *ASQ* Ages and Stages Questionnaire, *BASC-2* Behavior Assessment System for Children, *BSID-II and BSID-III* Bayley Scales of Infant Development–second and third editions, *BPI* Behavioral Problems Index, *CDI* Communicative Development Inventory, *CELF-P2* Clinical Evaluation of Language Fundamentals–Preschool, *CELF-4* Clinical Evaluation of Language Fundamentals Fourth Edition, *CLAMS* Clinical Linguistic Auditory Milestone Scale, *CTQ* Child Temperament Questionnaire, *DSM-IV* Diagnostic and Statistical Manual of Mental Disorders–4, *K-ABC* Kaufman Assessment Battery for Children, *K-ASQ* Korean–Ages and Stages Questionnaire, *NKT* Number Knowledge Test, *PIAT* Peabody Individual Achievement Test, *PLS-4* Preschool Language Scale–4, *PPVT* Peabody Picture Vocabulary Test, *WISC* Wechsler Intelligence Scale for Children

^a^Includes **11 longitudinal studies** [88, 90, 92, 100, 102, 112, 113, 119–122] from **8 unique samples**. Tomopoulos et al. [112] reported data from the Bellevue Project for Early Language, Literacy, and Education Success (BELLE); McKean et al. [121] reported data from the Early Language in Victoria Study (ELVS); Pagani et al. [90, 92] reported data from the Quebec Longitudinal Study of Child Development (QLSCD); Schmidt et al. [88] reported data from Project Viva; and Foster and Watkins [113], Christakis et al. [120] and Zimmerman and Christakis [119] reported data from the National Longitudinal Survey of Youth, Children, and Young Adults (NLSY-Child). Results are presented separately and participants are counted only once

^b^Serious risk of bias. Questionable validity and reliability of television duration exposure measure in all studies [88, 90, 92, 100, 102, 112, 113, 119–122]; poor reliability of Attention Problems subscale of the Child Behavior Checklist (ɑ =0.59) [102]; possible reporting bias, because the relationship between TV exposure and BMI at age 3 yr was analyzed despite not being described in the methods section [88]; two studies had unexplained missing data (34% and 40% missing) and the pattern of nonresponse indicates the reason for missing data may have been related to the outcome of interest [112, 121]; data were reported incompletely for the relationship between TV exposure and reading achievement [90]; the methods section of one study indicated that bivariate analysis would be performed, but included variables and the results of the analysis were not reported [121]

^c^The quality of evidence from longitudinal studies was downgraded from “low” to “very low” because of a serious risk of bias that diminished the level of confidence in the observed effects

^d^Includes **1 case-control study** [116]

^e^Serious risk of bias. Exposure measure was described in poor detail; questionable validity and reliability of television duration exposure measure; the Denver II Scale is useful for detecting severe developmental problems but has been criticized as being unreliable for predicting less severe or specific problems; the regression model that predicted developmental delay from a composite of “age of onset of TV viewing” and “TV viewing >2 h/day” was not pre-specified in the methods, and composite variables were not combined in analyses with other outcomes [116]

^f^The quality of evidence from the case-control study was downgraded from “low” to “very low” because of a serious risk of bias that diminished the level of confidence in the observed effects

^g^Includes **16 cross-sectional studies** [90, 94, 100, 104, 107–111, 114, 115, 117, 118, 121, 123, 124]. Zimmerman et al. [117] and Ferguson and Donnellan [124] reported data from the same sample. Results are presented separately and participants are counted only once

^h^Serious risk of bias. Potentially inappropriate sampling technique resulted in a sample with higher income and education than the overall population from which it was recruited [117, 124]; questionable validity and reliability of the exposure measure [90, 106–109, 111, 115, 117, 121–124]; questionable validity of exposure measure [94]; validation study showed overestimation of TV time exposure measure [110]; questionable validity and/or reliability of the outcome measure [109, 110]; unknown amount [109, 117] or between 28% and 60% [121, 124] of unexplained missing data and pattern of nonresponse indicates reason for missing data may have been related to the outcome of interest; incomplete reporting of exposure [109] and outcome [90, 110]; longitudinal relationships were reportedly collected but not reported in the results [115]; the methods section of one study indicated that bivariate analysis would be performed, but included variables and the results of the analysis were not reported [121]

^i^The quality of evidence from longitudinal studies was downgraded from “low” to “very low” because of a serious risk of bias that diminished the level of confidence in the observed effects

**Table 5:** The relationship between sedentary behaviour and bone and skeletal health

No. of participants (No. of studies)	Design	Quality assessment				Absolute effect	Quality
		Risk of bias	Inconsistency	Indirectness	Imprecision		
The mean age was 4.4 years. Data were collected cross-sectionally. Bone and skeletal health were assessed objectively using quantitative ultrasound.							
1512 (1)	Cross-sectional^a^	Serious risk of bias^b^	No serious inconsistency	No serious indirectness	Serious imprecision^c^	**Objectively measured sedentary time:** After adjusting for MVPA, accelerometer-derived **sedentary time** was no longer significantly associated with **bone stiffness index (SI)** in preschool children (β = -0.37; R^2^ = 19%; *p* = 0.28) [125]. **Screen-based sedentary behaviours:** There was no association between parent-reported **screen time** and **SI** (β = −0.04; R^2^ = 18.4%; *p* = 0.50) [125].	Very low^d^

*MVPA* moderate-to-vigorous physical activity, *SI* bone stiffness index

^a^Includes **1 cross-sectional study** that reported data from the Identification and prevention of dietary- and lifestyle-induced health effects in children and infants (IDEFICS) sample [125]

^b^Serious risk of bias. Study participants were selected by “judgment sample”; questionable validity and reliability of subjective and objective exposure measures, and of quantitative ultrasound for measurement of bone stiffness in children [125]

^c^Serious imprecision. It was not possible to estimate the precision of the findings since the study did not provide a measure of variability in the results

^d^The quality of evidence from the cross-sectional study was downgraded from “low” to “very low” because of: (1) a serious risk of bias that diminished the level of confidence in the observed effects, and (2) serious imprecision

**Table 6:** The relationship between sedentary behaviour and cardiometabolic health

No. of participants (No. of studies)	Design	Quality assessment				Absolute effect	Quality
		Risk of bias	Inconsistency	Indirectness	Imprecision		
The mean age was 3.1 years. Data were collected cross-sectionally. Cardiometabolic health was assessed using an objective measure of blood pressure.							
276 (1)	Cross-sectional^a^	Serious risk of bias^b^	No serious inconsistency	No serious indirectness	No serious imprecision	**Screen-based sedentary behaviours:** Watching **TV for ≥ 2 h/day** was not associated with **high blood pressure** (compared to <2 h/day, Prevalence Ratio = 0.9, 95% CI: 0.5, 1.4, *p* = 0.568) [126].	Very low^c^

^a^Includes **1 cross-sectional study** [126]

^b^Serious risk of bias. Unknown reliability and validity of the exposure measure [126]

^c^The quality of evidence from the cross-sectional study was downgraded from “low” to “very low” because of a serious risk of bias that diminished the level of confidence in the observed effects

**Table 7:** The relationship between sedentary behaviour and fitness

No. of participants (No. of studies)	Design	Quality assessment				Absolute effect	Quality
		Risk of bias	Inconsistency	Indirectness	Imprecision		
The mean age at exposure measurement ranged from ~29 to 53 months (~2.4 to 4.4 yr). Data were collected longitudinally up to 8 years of follow-up. Fitness was assessed as: lower body explosive strength (standing long jump) and fitness level (parent-report level relative to other children).							
1314 (2)	Longitudinal^a^	Serious risk of bias^b^	No serious inconsistency	Serious indirectness^c^	No serious imprecision	**Screen-based sedentary behaviours:** **Higher TV time** (hr/day) at age ~29 mo was unfavourably associated with **standing long-jump performance** (cm) at age 97.8 mo (B = −0.361; 95% CI: −0.576, −0.145; *p* < 0.001) [89] and **physical fitness level** (scale from −2 to 2) in Grade 4 (β = −0.09, SE = 0.0004; B = −0.01, 95% CI: −0.002, −0.02; *p* < 0.01) [90]. A greater increase in **TV time** (hr/week) between age ~29 and ~53 months was unfavourably associated with **standing long-jump performance** (cm) at age 97.8 months (B = −0.285; 95% CI: −0.436,-0.134; *p* < 0.01) [89] and **physical fitness level** (scale from −2 to 2, relative to other children) in Grade 4 (β = −0.10, SE = 0.0003, *p* < 0.01) [90].	Very low^d^

^a^Includes **2 longitudinal studies** [89, 90] from **1 unique sample** (QLSCD)

^b^Serious risk of bias. Questionable reliability and validity of the exposure [89, 90] and outcome [90] measures; large unexplained loss to follow-up and unclear if included participants differed from missing participants [89]; controlled for physical activity [89, 90]

^c^Serious indirectness. Differences between outcomes of included studies and those of interest; only one study reported a measure of lower-body musculoskeletal fitness (lower-body strength assessed by standing long-jump performance) [89], and one study reported an indirect measure of physical fitness [90]. No studies reported direct measures of total body musculoskeletal or cardiovascular fitness

^d^The quality of evidence from the longitudinal studies was downgraded from “low” to “very low” because of: 1) a serious risk of bias that diminished the level of confidence in the observed effects, and 2) indirectness of the comparisons being assessed

**Table 8:** High-level summary of findings by health indicator

Health indicator	Number of studies	Quality of evidence	Summary of findings: Number of studies reporting unfavourable/null/favourable associations with at least one health indicator measure by SB type^a^
Critical			
Adiposity	60	Very low to moderate	**Objectively measured sedentary time:**
			*Sedentary time in 30-min bouts (accelerometer-derived):* null (1)
			*Total sedentary time (accelerometer-derived):* unfavourable (1), null (12)
			**Screen-based sedentary behaviours:**
			*Computer (duration, frequency):* unfavourable (1), null (6)
			*Internet (duration):* null (1)
			*Total screen time (duration):* unfavourable (9), null (14)
			*TV time (duration):* unfavourable (20), null (24), favourable (2)
			*Video games (duration):* unfavourable (1)
			*Other screens (DVDs/videos; duration):* unfavourable (1), null (1)
			**Other sedentary behaviours:**
			*Reading (duration):* null (1)
			*Sitting (baby seats, car, sedentary quiet play; duration):* unfavourable (2), null (4), favourable (1)
Motor development	7	Very low	**Objectively measured sedentary time:**
			*Sedentary time in 30-min bouts (accelerometer-derived):* null (1)
			*Total sedentary time (accelerometer-derived):* unfavourable (1), null (2)
			**Screen-based sedentary behaviours:**
			*TV time (duration):* unfavourable (2), null (3)
			**Other sedentary behaviours:**
			*Sitting (baby carrier/sling, car seat, high chair/other chair, playpen, stroller; duration):* null (1), favourable (1)
			*Supine position (duration):* unfavourable (1), null (1)
Psychosocial health	15	Very low to moderate	**Objectively measured sedentary time:**
			*Total sedentary time (accelerometer-derived):* null (1)
			**Screen-based sedentary behaviours:**
			*Computer (duration):* unfavourable (1), null (1)
			*Total screen time (duration):* unfavourable (1)
			*TV time (duration):* unfavourable (9), null (11), favourable (2)
Cognitive development	25	Very low	**Objectively measured sedentary time:**
			*Total sedentary time (accelerometer-derived):* null (1)
			**Screen-based sedentary behaviours:**
			*Computer (yes, no):* null (1)
			*Mobile phone use (yes, no):* unfavourable (1)
			*Total screen time (duration):* unfavourable (1)
			*TV time (duration):* unfavourable (11), null (10), favourable (1)
			*Video games (duration):* null (1)
			*Other screens (total or electronic media exposure; duration):* unfavourable (2), null (1)
			**Other sedentary behaviours:**
			*Reading (duration, frequency):* null (2), favourable (3)
			*Storytelling with parents (frequency):* null (2), favourable (2)
Important			
Bone and skeletal health	1	Very low	**Screen-based sedentary behaviours:**
			*Screen time (duration):* null (1)
			**Objectively measured sedentary time:**
			*Total sedentary time (accelerometer-derived):* null (1)
Cardiometabolic health	1	Very low	**Screen-based sedentary behaviours:**
			*TV time (duration):* null (1)
Fitness	2	Very low	**Screen-based sedentary behaviours:**
			*TV time (duration):* unfavourable (2)
Risks / harms	0	N/A	N/A

^a^Note that the number of studies reporting unfavourable/null/favourable associations does not sum to the total number of studies for a given indicator since some studies reported mixed associations. N/A: not applicable

### Quality of evidence

Overall, the quality of evidence ranged from “very low” to “moderate” across study designs and health indicators. The most common reason for downgrading the quality of evidence was because of a serious risk of bias that reduced the level of confidence in the observed effects. Common sources of bias included: not accounting for potentially important confounders or mediating factors (e.g., diet); the use of potentially inappropriate measurement tools (e.g., exposure or outcome measures with unknown reliability and/or validity); and an unknown amount of, or reasons for, missing data. The quality of evidence was not upgraded in any instance. For specific details regarding the quality of evidence by study design and health indicator, see Tables 1, 2, 3, 4, 5, 6 and 7.

### Data synthesis

Meta-analyses could not be performed because of heterogeneity in the sedentary behaviour exposure and health indicators (statistical, clinical, and methodological). Instead, narrative syntheses are presented. Unless otherwise stated, results did not differ by sex, age, or specific sub-indicator within the eight health indicator categories. Within each health indicator, results are presented first by study design, then by type of sedentary behaviour exposure (objectively measured sedentary time, screen-based sedentary behaviours, and other sedentary behaviours), and finally by sub-indicator (i.e., specific measures of the eight health indicators). The reader is referred to the Additional file 2: Tables S1-S7 for statistic values and additional details.

### Critical (primary) health indicators

#### Adiposity

The relationships between sedentary behaviour and adiposity were examined in 60 studies (see Table 1 and Additional file 2: Table S1) [31–90]. Study designs were: randomized controlled trial (*n* = 1) [34], longitudinal (*n* = 13) [33, 45, 54, 81–90], case-control (*n* = 2) [35, 36], and cross-sectional design or also reported cross-sectional findings (*n* = 47) [31–33, 37–80]. Indicators of adiposity (e.g., body mass index [BMI]) were measured objectively (e.g., measured by dual-energy X-ray absorptiometry) or assessed subjectively (e.g., parent-reported height and weight; see Table 1 for summary of measures). The quality of evidence ranged from “very low” to “moderate” across study designs (Table 1).

In the randomized controlled trial of an intervention to reduce screen time, screen time was significantly lower for preschoolers in the intervention versus control group at 2, 6, and 9 months post-intervention [34]. BMI z-scores were not different between the intervention and control groups at baseline or 9-month follow-up, but BMI z-scores increased in both groups [34] (Additional file 2: Table S1).

Among the 13 longitudinal studies, sedentary behaviour was assessed from age ~9 months to 4.95 years as screen-based (i.e., computer time, frequency of playing computer games, time watching DVDs, TV time, and total screen time) or other sedentary behaviours (i.e., time spent in the car or in baby seats). Adiposity indicators were assessed between ~1.25 and 12 years follow-up.

For screen-based sedentary behaviours, computer time [85], and frequency of playing computer games [82] at age 4.8 years were not associated with total fat mass or lean mass, or weight status, at ~6 and 12 years of follow-up respectively. Time watching DVDs at ages ~3–4 years was unfavourably associated with weight status at kindergarten entry [83]. Total screen time in toddlers was unfavourably associated with weight status at preschool or school age in 2/3 studies [33, 84]. In the third study, total screen time was not associated with weight status [87].

Ten longitudinal studies examined the relationships between TV time (at ages ranging from ~6 months to 4.8 years) and adiposity indicators at ~1.5 to 12 years of follow-up. Of these, unfavourable associations were reported in 6/10 studies [33, 54, 81, 83, 88, 90], null associations in 1/10 studies [86], and mixed unfavourable and null associations in 3/10 studies [82, 85, 89]. Specifically, TV time was prospectively unfavourably associated with these adiposity indicators: BMI z-score in 1/1 studies [88], BMI in 2/3 studies [54, 90], % change in BMI and % change in waist-to-height ratio in 1/1 studies [33], fat mass in 1/1 studies [82], and weight status in 2/2 studies [81, 83] (Additional file 2: Table S1). TV time at age ~3 years was not associated with the rate of weight gain from ages 3 to 5 years [86]. TV time at age 2.4 years was not associated with waist circumference at age 10.15 years, but the change in TV time from ages 2.4 to 4.4 years was unfavourably associated with waist circumference at age 10.15 years [89]. TV time at age 3.2 years was unfavourably associated with fat mass at age 15 years.

Regarding other sedentary behaviours, types of sitting were examined in three longitudinal studies. Among preschoolers, time in the car was not prospectively associated with adiposity indicators in 2/2 studies [82, 85]; however, among infants there were mixed unfavourable, null, and favourable associations between time in baby seats and adiposity indicators [45]. Specifically, time in baby seats at age ~9 months was unfavourably associated with a change in weight-for-height and change in weight-for-age from ~9 months to 2 years, was not associated with weight-for-height or weight-for-age at age ~2 years, and was favourably associated with waist circumference-for-age at age ~2 years and change in waist circumference-for-age from ~9 months to 2 years [45] (Additional file 2: Table S1).

In the two case-control studies, TV time [35, 36] and total sedentary time (assessed by one-day parent-recall) [36] were not significantly different between preschoolers with overweight/obese (case group) or normal-weight (control group) status, but watching TV for ≥1 h/day was unfavourably associated with having overweight status [35] (Additional file 2: Table S1).

Among the 47 cross-sectional studies, sedentary behaviour was assessed as accelerometer-derived sedentary time, screen-based (i.e., computer time, time playing inactive video games, using the internet, watching DVDs/videos, TV time, and total screen time), or other sedentary behaviours (i.e., sedentary quiet play, and time in the car or in baby seats).

The relationships between accelerometer-derived sedentary time and adiposity indicators in toddlers and preschoolers were examined in 11 cross-sectional studies; null associations were reported in 10/11 studies [37–40, 47, 53, 60, 75, 78, 80] and mixed unfavourable and null associations in 1/11 studies [77] (Additional file 2: Table S1). Specifically, total sedentary time was not associated with: % body fat, fat mass index, trunk fat mass index, or lean mass index in 1/1 studies [78]; BMI in 1/1 studies [75]; BMI z-score in 4/4 studies [37–39, 47]; and weight status in 4/4 studies [40, 53, 60, 80] (Additional file 2: Table S1). Total sedentary time was not associated with BMI z-score percentile or waist circumference, but was associated with waist circumference percentile in girls (not boys) in 1/1 studies [77]. Accelerometer-derived sedentary time in 30-min bouts was not associated with weight status [40].

For screen-based sedentary behaviours, time playing inactive video games was unfavourably associated with preschoolers’ BMI percentile, but using the internet and watching DVDs/videos were not cross-sectionally associated with BMI percentile [69] (Additional file 2: Table S1). Computer time was not associated with preschoolers’ weight status in 4/4 studies [63, 67, 71, 79], but was unfavourably associated with sum of skinfold thicknesses in 1/1 studies [71].

The relationships between total screen time and adiposity indicators were examined in 18 cross-sectional studies; unfavourable associations were reported in 6/18 studies [32, 33, 46, 50, 59, 73], null associations in 10/18 studies [44, 52, 57, 58, 62, 64, 65, 71, 72, 79], and mixed unfavourable and null associations in 2/18 studies [41, 61] (Additional file 2: Table S1). Of these, screen time was unfavourably associated with: sum of skinfold thicknesses in 0/1 studies, waist-to-height ratio in 1/1 studies [33], BMI in 2/2 studies [46, 50], and at least one measure of weight status in 6/16 studies [32, 33, 41, 59, 61, 73]. Only one of these studies was in infants (no association between screen time and weight status [58]); the rest were in toddlers and preschoolers.

The relationships between TV time and adiposity indicators in toddlers and preschoolers were examined in 23 cross-sectional studies; unfavourable associations were reported in 5/23 studies [33, 55, 66, 67, 71], null associations in 11/23 studies [31, 42, 43, 49, 50, 56, 60, 63, 69, 75, 76], mixed unfavourable and null associations in 5/23 studies [48, 51, 54, 68, 79], mixed null and favourable associations in 1/23 studies [74], and mixed unfavourable, null, and favourable associations in 1/23 studies [70] (Additional file 2: Table S1). Of these, TV time was unfavourably associated with: waist-to-hip ratio in 0/1 studies, waist-to-height ratio in 1/1 studies [33], triceps skinfold thickness in 0/1 studies, waist circumference in 0/2 studies, sum of skinfolds in 1/3 studies [71], BMI percentile in 0/1 studies, BMI in 2/11 studies [51, 54], and at least one measure of weight status in 9/13 studies [33, 48, 55, 66–68, 70, 71, 79]. Weekday (but not weekend) TV time was favourably associated with the ratio of triceps to subscapular skinfold thickness (representing limb-to-trunk adiposity ratio) in girls but not boys in 1/1 studies [74]. TV time was favourably associated with BMI z-score in boys but not girls in 1/1 studies [70] (Additional file 2: Table S1).

Regarding other sedentary behaviours, infants’ time in baby seats was not cross-sectionally associated with weight-for-height/age or waist circumference-for-age [45]. Among preschoolers, time using books [69] was not associated with BMI percentile [69]. Sedentary quiet play (defined as “e.g., looking into books, playing with blocks, playing with dolls, drawing, construction”) on weekdays or weekend days was not associated with weight status in boys [79]. In girls, sedentary quiet play on weekend days (but not weekdays) was unfavourably associated with weight status [79].

#### Motor development

The relationships between sedentary behaviour and motor development were examined in seven studies (see Table 2 and Additional file 2: Table S2) [37, 40, 88, 91–94]. Study designs were: longitudinal (*n* = 3) [88, 91, 92], and cross-sectional (*n* = 4) [37, 40, 93, 94]. Indicators of motor development were measured objectively (e.g., visual-motor abilities measured using the Wide-Range Assessment of Visual Motor Ability) or assessed subjectively by parent-report (e.g., age at first sitting; see Table 2 for summary of measures). The quality of evidence was “very low” across study designs (Table 2).

Among the three longitudinal studies, sedentary behaviour was assessed from age 3.9 months to 2.4 years as screen-based (i.e., TV time) or other sedentary behaviours (i.e., time in a baby carrier/sling, car seat, high chair/other chair, playpen, or stroller). Motor development indicators were assessed after 1.3 to 3 years of follow-up. For screen-based sedentary behaviours, TV time was not prospectively associated with age at first sitting, crawling, or walking [91], visual-motor abilities [88], or object control [92], but was unfavourably associated with locomotion skills [92].

Regarding other sedentary behaviours, infants’ time in a baby carrier/sling, stroller, high chair or other chair, or playpen was not associated with age at first sitting, crawling, or walking [91] (Additional file 2: Table S2). Greater time in a car seat at age ~9 months was associated with earlier (i.e., favourable) age at first sitting and age at first crawling, but was not associated with age at first walking; time spent in a car seat at ages ~4 months and 1.7 years was not associated with age at first sitting, crawling, or walking [91].

In the 4 cross-sectional studies, sedentary behaviour was assessed as accelerometer-derived sedentary time, screen-based (i.e., TV time), or other sedentary behaviours (i.e., time in the supine position). The relationships between accelerometer-derived sedentary time and motor development were examined in two of the cross-sectional studies. Total sedentary time was not associated with motor skills at age ~2 years [40] or ~3 to 4 years [37], or with object control skills at age ~3 to 4 years [37], but % sedentary time was unfavourably associated with locomotor skills at age ~3 to 4 years [37]. The number of 30-min bouts of sedentary behaviour was not associated with motor skills [40].

For screen-based sedentary behaviours, TV time was unfavourably associated with motor skill development; children with delayed motor skill development spent more time watching TV compared to children with typical motor skill development, and children who were frequently exposed to TV (>0 h/day for children <2 years and >2 h/day for children ≥2 years) were more likely to have delayed motor skill development than those who were infrequently exposed [94].

For other sedentary behaviours, time in the supine position before 6 months of age was not associated with gross motor performance, but time in the supine position after age 6 months was unfavourably associated with gross motor performance [93].

#### Psychosocial health

The relationships between sedentary behaviour and psychosocial health in toddlers and preschoolers were examined in 15 studies (no studies in infants; see Table 3 and Additional file 2: Table S3) [34, 90, 92, 95–106]. Study designs were: randomized controlled trial (*n* = 1) [34], longitudinal (*n* = 9) [90, 92, 95–97, 99, 100, 102, 103], and cross-sectional design or additionally reported cross-sectional findings (*n* = 7) [98, 100, 101, 103–106]. Indicators of psychosocial health (e.g., aggression, symptoms of anxiety and depression) were assessed subjectively by parent-, teacher-, or self-report using questionnaires (see Table 3 for summary of measures). The quality of evidence ranged from “very low” to “moderate” across study designs (Table 3).

In the randomized controlled trial of an intervention to reduce screen time, preschoolers’ screen time was significantly lower in the intervention versus control group at 2, 6, and 9 months post-intervention [34]. Aggressive and delinquent behaviours were not significantly different between the intervention and control groups at baseline, but were significantly lower in the intervention versus control group at 9-months post-intervention [34] (Additional file 2: Table S3).

Among the nine longitudinal studies, screen-based sedentary behaviour (i.e., time e-gaming or on a computer, or TV time) was assessed from age ~1.5 to 5 years. Psychosocial health indicators were assessed after ~1 to 9.5 years of follow-up.

Time spent e-gaming or on a computer (on weekdays or weekend days) at age 4.3 years was not associated with being at risk for the following at age 6.3 years: peer problems, self-esteem problems, social well-being problems, social functioning problems, or family functioning problems [96]. Time spent e-gaming or on a computer on weekdays (but not weekend days) at age 4.3 years was unfavourably associated with being at risk for emotional problems at age 6.3 years in girls but not boys [96] (Additional file 2: Table S3).

The relationships between TV time among toddlers/preschoolers and psychosocial health indicators at follow-up were examined in nine longitudinal studies; unfavourable associations were reported in 2/9 studies [95, 103], null associations in 1/9 studies [100], mixed unfavourable and null associations in 5/9 studies [90, 92, 96, 97, 99], and mixed null and favourable associations in 1/9 studies [102] (Additional file 2: Table S3). Specifically, TV time was prospectively unfavourably associated with the following psychosocial health indicators: victimization [90, 95], victimization by classmates [92], being a victim of bullying [97], being a bully [103], externalizing problems [99], and being at risk for family functioning problems [96] (Additional file 2: Table S3). Null associations were reported between TV time and emotional symptoms [100]; conduct problems [100]; peer-problems [100]; prosocial behaviour [92, 100]; externalizing problems [99, 102]; anxiety or depressive symptoms [92, 102]; physical aggression [100] or aggressive behaviour [102]; being a bully, being a victim of bullying, or being a bully-victim [97]; being at risk for emotional problems, peer problems, self-esteem problems, emotional well-being problems, or social functioning problems [96]; and co-operation, self-control, assertion, responsibility, or total social skills [102]. TV time at age ~2.5 years was favourably associated with emotional reactivity scores after ~3 years of follow-up [102].

In the 7 cross-sectional studies, sedentary behaviour was assessed as accelerometer-derived total sedentary time or screen-based (i.e., TV time) sedentary behaviour. Total sedentary time (accelerometer-derived) was not cross-sectionally associated with preschoolers’ psychosocial health indicators (soothability, sociability, or emotionality) [104].

The relationships between TV time and psychosocial health indicators in toddlers and preschoolers were examined in six cross-sectional studies; unfavourable associations were reported in 2/6 studies [101, 103], null associations in 2/6 studies [100, 106], mixed unfavourable and null associations in 1/6 studies [105], and mixed unfavourable and favourable associations in 1/6 studies [98]. Specifically, TV time was unfavourably associated with aggression [101], bullying [103], total externalizing behaviour problems [105], and total behaviour problems [105]. Null associations were reported between TV time and emotional symptoms, conduct problems, peer problems, and prosocial behaviour [100], aggression toward a sibling [106], and internalizing behaviour problems [105]. TV time was favourably associated with social-emotional competence in one study [98].

#### Cognitive development

The relationships between sedentary behaviour and cognitive development were examined in 25 studies (see Table 4 and Additional file 2: Table S4) [88, 90, 92, 94, 100, 102, 104, 107–124]. Study designs were: longitudinal (*n* = 11) [88, 90, 92, 100, 102, 112, 113, 119–122], case-control (n = 1) [116], and cross-sectional design or additionally reported cross-sectional findings (*n* = 16) [90, 94, 100, 104, 107–111, 114, 115, 117, 118, 121, 123, 124]. Indicators of cognitive development were measured objectively (e.g., working memory capacity measured using the Memory for Digit Span test) or assessed subjectively by parent-report interview or questionnaire (e.g., receptive vocabulary; see Table 4 for summary of measures). The quality of evidence was “very low” across study designs (Table 4).

Among the 11 longitudinal studies, sedentary behaviour was assessed from age ~6 months to 5 years as screen-based (i.e., electronic media exposure and TV time) or other sedentary behaviours (i.e., frequency of parents reading). Cognitive development indicators were assessed after ~8 months to 8 years of follow-up.

For screen-based sedentary behaviours, electronic media exposure at age ~6 months was unfavourably associated with the following at age 14 months: cognitive development, language development, and auditory comprehension [112]. The relationships between TV time and cognitive development indicators in toddlers and preschoolers were examined in 10 longitudinal studies; unfavourable associations were reported in 5/10 studies [90, 92, 100, 120, 121], null associations in 4/10 studies [88, 102, 113, 122], and mixed unfavourable, null, and favourable associations in 1/10 studies [119]. Specifically, TV time was prospectively unfavourably associated with the following cognitive development indicators: rate of change in language development [121], receptive vocabulary and number knowledge [92], classroom engagement [90, 92], mathematical achievement [90], attentional problems [120], and hyperactivity-inattention [100] (Additional file 2: Table S4).

Regarding other sedentary behaviours, the frequency of parents reading to their child from ages ~8 months to 4 years was favourably associated with both language development at age 4 years and the rate of change in language development between ages 5 to 7 years [121] (Additional file 2: Table S4).

In the case-control study, toddlers with language delay (cases) had significantly greater TV time than those with normal language development (controls) [116]. Compared with toddlers who viewed ≤2 h/day TV time, those with >2 h/day TV time had increased odds of language delay [116].

In the 16 cross-sectional studies, sedentary behaviour was assessed as accelerometer-derived sedentary time, screen-based (i.e., computer use, mobile phone use, time playing inactive video games, TV time, total media exposure, and total screen time), or other sedentary behaviours (i.e., reading or storytelling with parents). Only one cross-sectional study examined the association between accelerometer-derived total sedentary time and cognitive development indicators; total sedentary time was not associated with attention span in preschoolers [104].

For screen-based sedentary behaviours, computer use was not associated with the prevalence of speech disorders, but mobile phone use (any versus none) was unfavourably associated with speech disorders in toddlers and preschoolers [109]. Time playing inactive video games was not associated with hyperactivity or attention problems in preschoolers [107]. Total screen time was unfavourably associated with communication development in toddlers [111], and total media exposure was unfavourably associated with receptive language development and expressive language development in infants and toddlers aged ~6 months to 1.3 years, but not with total language development in toddlers aged ~1.4 to 2.3 years [124].

The relationships between TV time and cognitive development in toddlers and preschoolers were examined in nine cross-sectional studies; unfavourable associations were reported in 3/9 studies [94, 108, 123], null associations in 5/9 studies [90, 100, 114, 115, 121], and mixed unfavourable and null associations in 1/9 studies [118] (see Additional file 2: Table S4 for statistics). Specifically, TV time was unfavourably associated with language development or capacity in 2/5 studies [94, 108] (Additional file 2: Table S4). TV time was unfavourably associated with delayed executive function [123] and cognitive development [94], but was not associated with cognitive ability [90] (Additional file 2: Table S4). TV time was not associated with hyperactivity-inattention in toddlers [100], and was unfavourably associated with teacher-reported, but not parent-reported, attention-deficit/hyperactivity disorder (ADHD) symptoms in preschoolers [118] (Additional file 2: Table S4).

Regarding other sedentary behaviours, the relationships between reading with parents and cognitive development indicators in infants, toddlers, and preschoolers were examined in three cross-sectional studies [110, 117, 124], two of which analyzed the same dataset in different ways [117, 124]; reading with parents was favourably associated with language development percentile in both infants and toddlers [117], but was not associated with absolute language development in toddlers (not analyzed in infants) [124]. Reading with parents was favourably associated with absolute receptive language development, but not expressive language development, in infants [124]. In the third study, reading with parents was not associated with executive function in preschoolers [110]. Storytelling with parents was favourably associated with language development percentile in infants [117]. In toddlers, storytelling was favourably associated with absolute language development [124], but not language development percentile [117]. Storytelling with parents was favourably associated with absolute receptive language development, but not expressive language development, in infants [124] (Additional file 2: Table S4).

### Important (secondary) health indicators

#### Bone and skeletal health

The relationship between sedentary behaviour and bone and skeletal health in preschoolers was examined in one cross-sectional study (see Table 5 and Additional file 2: Table S5) [125]. The quality of evidence was rated as “very low”. As summarized in Table 5, parent-reported screen time and accelerometer-derived total sedentary time were not associated with bone stiffness index in preschool children [125]. No other indices of bone and skeletal health were examined.

#### Cardiometabolic health

The relationship between sedentary behaviour and cardiometabolic health in preschoolers was examined in one cross-sectional study (see Table 6 and Additional file 2: Table S6) [126]. The quality of evidence was rated as “very low”. Watching TV for ≥2 h/day was not associated with high blood pressure in preschool children [126]. No other cardiometabolic biomarkers were examined.

#### Fitness

The relationship between sedentary behaviour and fitness in toddlers and preschoolers was examined in two longitudinal studies (no studies in infants; see Table 7 and Additional file 2: Table S7) [89, 90]. The quality of evidence was rated as “very low”.

As summarized in Table 7, greater TV time at age ~2.4 years was unfavourably associated with standing long-jump performance at age ~8.2 years [89] and physical fitness level (assessed as “relative to other children” via parent-report) in Grade 4 (age ~10 years) [90]. A greater increase in TV time between age ~2.4 and ~4.4 years was unfavourably associated with standing long-jump performance at age 8.2 years [89] and physical fitness level in Grade 4 [90].

#### Risks/harm

No studies examined harms associated with sedentary behaviour.

## Discussion

The objective of this study was to perform a systematic review that examined the relationships between sedentary behaviours and health indicators in children 0 to 4 years, and to determine what doses of sedentary behaviours (i.e., duration, patterns [frequency, interruptions], and type) were associated with health indicators. The main findings are the following: 1) associations between objectively measured total sedentary time and health indicators (adiposity and motor development) were predominantly null; 2) associations between screen-based sedentary behaviours and health indicators (adiposity, motor or cognitive development, and psychosocial health) were largely unfavourable or null; 3) associations between reading or storytelling and cognitive development were favourable or null; and 4) associations between time spent seated (e.g., in baby seats, car seats, high chairs or strollers) or in the supine position and health indicators (adiposity, motor development) were primarily unfavourable or null. Few studies examined indicators of bone and skeletal health, cardiometabolic health, or fitness, and no studies reported on risks or harms (e.g., torticollis, injuries) associated with sedentary behaviours. These findings suggest that, in the early years, total sedentary time may have a negligible impact on health, but the way that time is spent is important, with screen-based and seated/supine sedentary behaviours likely to have unfavourable or null health effects (unlikely to have favourable effects), and interactive non-screen-based activities such as reading and storytelling likely to have favourable health effects. A summary of the findings is presented in Table 8.

The finding that there are no associations between objectively measured total sedentary time and health indicators in the early years (0 to 4 years) is in contrast to the relationships in older age groups, in particular adults [4, 127]. While this suggests that in the early years a certain amount of sedentary behaviour may be innocuous and perhaps even necessary for healthy growth and development, these findings should be interpreted with caution. First, objectively measured total sedentary time was examined only in cross-sectional studies, and a plausible explanation for the perceived lack of association between total sedentary time and health indicators is that there had simply been insufficient time for those effects to manifest, rather than there being no effect. This hypothesis is supported by comparison of findings from longitudinal and cross-sectional studies for subsets of total sedentary behaviour. For instance, 9/10 (90%) longitudinal studies reported at least one unfavourable association between TV time and adiposity indicators, compared to only 11/22 (50%) cross-sectional studies. However, total sedentary time was examined only in relation to adiposity and motor development (and in one study each for indicators of psychosocial health, cognitive development, and bone and skeletal health); it remains possible that total sedentary time is associated with other health indicators, particularly those likely to be acutely affected in the early years, such as cognitive development. More well-designed studies with objective measures of sedentary behaviour are needed.

Second, in the present review, studies that utilized accelerometry measures applied a range of sampling intervals (epochs) and cut-points. Given that these measurement parameters influence the amount of sedentary behaviour captured [128, 129], individual studies may have under- or overestimated the total amount of sedentary time and may therefore have resulted in an underestimation or overestimation of true effects. However, Byun et al. applied three different accelerometry cut-points in two cross-sectional datasets to test whether this would influence the findings, and found no association between total sedentary time and BMI z-score, regardless of the cut-points used [38]. Nonetheless, the most appropriate way to objectively measure sedentary behaviour in the early years is still unknown and remains an important area for future work.

Lastly, total sedentary time was not objectively assessed in any studies in the infant age group; however, such measures may not be meaningful in non-ambulatory infants. Although the associations between total sedentary time and health indicators were primarily null, the present data do not allow for recommendations regarding “appropriate” amounts or patterning (e.g., breaks) of total sedentary time.

Regarding screen-based sedentary behaviours, the present findings support and extend those of the earlier systematic review [2]; overall, screen time (namely TV time) was unfavourably associated with a range of health indicators. Notably, TV time was the predominant measure of screen-based behaviour, followed by total screen time, with only eight studies reporting relationships between computer use and any health indicator; two studies for each of DVDs/videos, electronic/total media exposure, and inactive video games; and one study for mobile phone and internet use. Findings for these other screen exposures were mixed (unfavourable or null), and suggest no benefits and some potential for harm. Although it seems intuitive that different types of screens may exert different effects (e.g., interacting on video-chat versus passive screen use), research on children’s use of such technologies lags behind their adoption [130]; this is a substantial research gap. Importantly, screen-based behaviours are used as a proxy for sedentary behaviour; however, it is uncertain whether children in this age group are actually sedentary while using screens, and there may be screen-related health effects that are independent of the “lack of movement” [131, 132]. Notwithstanding these limitations, the present findings indicate that less screen-based sedentary behaviour is better for optimal health in the early years of life.

Other sedentary behaviour exposures were less frequently examined, and findings were mixed. In general, reading [110, 117, 121, 124] and storytelling [117, 124] were favourably associated with cognitive development, while various types of time spent seated (e.g., in a car seat, high chair, or stroller) had mixed unfavourable and null associations with indicators of adiposity and motor development [45, 81, 82, 91]. An age-dependent effect was observed in the only study that assessed time in the supine position; time spent supine before 6 months of age was not associated with gross motor performance, but greater time in the supine position after age 6 months was associated with worse gross motor performance [93]. Overall, there was a paucity of data regarding the relationships between other types of sedentary behaviours and health indicators. Research shows that children are spending ~7 h of the day in sedentary pursuits [15], and ~2 h of these are occupied by screen time [15]; this leaves an additional 5 h that are unaccounted for. Other types of sedentary behaviours are thus highly understudied, and this is an important research gap.

Most studies examined the duration of sedentary behaviours in relation to health indicators, with only three studies specifically examining the impact of patterns of behaviour (i.e., breaks, frequency). Specifically, there was no association between accelerometer-derived sedentary time in 30-min bouts and indicators of adiposity and motor development [40], or between the frequency of playing computer games and adiposity indicators [82], but there were favourable associations between the frequency of parents reading or storytelling and child cognitive development [121]. These findings are consistent with those of studies that examined sedentary behaviour duration; however, it remains difficult to draw conclusions regarding patterns of sedentary behaviour for optimal health in the early years.

### Strengths, limitations, and future directions

Strengths of this review include the use of a comprehensive search strategy that was developed and peer-reviewed by librarians with expertise in systematic reviews, as well as inclusion of all study designs and a broad range of health indicators that represent various dimensions of health. Rigorous methodological standards were used in this review, including application of the GRADE framework to guide the review process and assess the quality of the evidence [27]. To our knowledge, this systematic review is the first to synthesize the evidence regarding the relationships between objectively and subjectively measured sedentary behaviour across the most comprehensive range of health indicators in children in the early years of life.

In terms of limitations, sample size restrictions were imposed for feasibility reasons and to maximize generalizability, but it is possible that studies with smaller sample sizes might have provided additional insight. Further, because of heterogeneity in the measurement of sedentary behaviour and health indicators, meta-analyses were not possible and all studies were weighted equally in the narrative synthesis. The direction of associations (i.e., unfavourable, null, favourable) was based on statistical significance; clinical significance was not considered.

Although an abundance of evidence was synthesized in this review, several limitations of this area of research were identified that remain to be addressed. As mentioned, data were limited regarding the relationships between sedentary behaviour and four relevant health indicators (two or fewer studies for each of bone and skeletal health, cardiometabolic health, fitness, and risks/harms); TV time was the primary sedentary exposure, with few studies examining “other” types of screens (e.g., tablets, mobile phones) or sedentary behaviours (e.g., reading, puzzles); and objective measures of total sedentary time were employed only in cross-sectional studies. Although adiposity was the most commonly measured health indicator (60 studies), direct measures of adiposity were used in only two studies [78, 82] while the remainder used surrogate measures such as BMI. Only one randomized controlled study was included in the present review, and the quality of the evidence ranged from “very low” to “moderate” across the study designs and health indicators. There is a need for high-quality studies with strong designs to better establish the magnitude of effects and the nature of dose-response gradients (if applicable), to assess cause-and-effect relationships, and to examine potential subgroup differences (e.g., based on age, sex, or socio-economic status). When RCTs are not possible because of the inherent challenges of research in this age group, quasi-experimental or longitudinal designs that use validated sedentary behaviour measures and outcome measures that are sensitive enough to detect changes are recommended.

Across the health indicators, the most common reason for downgrading the quality of evidence was the serious risk of bias associated with sedentary behaviour measures with no known psychometric properties. Consequently, development and use of reliable and valid subjective measures of sedentary behaviour are needed. Defining and measuring sedentary behaviour in young children, particularly in non-ambulatory infants, remains a challenge. For instance, infants in the supine position may be vigorously moving arms and legs, and thus being “active”, but existing questionnaire-based measures do not capture this. Future research using inclinometers, which can more accurately capture postures [133], as well as limb-worn devices, will help to address the challenges associated with quantifying sedentary behaviours in the early years. Finally, the question of whether different types of sedentary behaviour “content” (e.g., educational versus recreational TV programming) exert different health effects was beyond the scope of this review, and remains an important area for future work.

## Conclusions

This systematic review synthesized findings from 96 studies with ~200,000 participants in 33 countries around the world; the quality of the evidence ranged from “very low” to “moderate”. In summary, the findings demonstrate that in the early years (0 to 4 years), total sedentary time may have a negligible impact on health, but the quality of that time is important, with screen-based and seated/supine sedentary behaviours likely to have no benefit and a potential for harm, and interactive non-screen-based activities such as reading with caregivers having favourable health effects. These findings continue to support the importance of minimizing screen time for disease prevention and health promotion in the early years [2, 9], and also highlight the potential benefits of interactive non-screen-based sedentary behaviours such as reading and storytelling. There is a need for additional research using valid and reliable measures and high-quality study designs, to more definitively establish the relationships between sedentary behaviours and health indicators, and to provide insight into the appropriate dose (durations, patterns, type) of sedentary behaviour for optimal health in the early years.

## Additional files

Additional file 1: Search strategies. (PDF 58 kb)

Additional file 2: Supplementary **Tables S1-S7.** (PDF 1166 kb)

## Abbreviations

ADHD: Attention-deficit/hyperactivity disorder
BMI: Body mass index
GRADE: Grading of recommendations, assessment, development and evaluation
METS: Metabolic equivalent
PICOS: Population, intervention, comparison, outcomes and study designs framework
PRISMA: Preferred reporting items for systematic reviews and meta-analyses
RCT: Randomized controlled trial
SB: Sedentary behaviour
TV: Television
