# Supplementary material for: Systematic review of the relationships between sedentary behaviour and health indicators in the early years (0–4 years)
Source: BMC Public Health. 2017 Nov 20;17(Suppl 5):868. doi: 10.1186/s12889-017-4849-8 (PMC5773886; doi:10.1186/s12889-017-4849-8)
Supplement: Supplementary file 1 — Search strategies. (PDF 58 kb) [file 12889_2017_4849_MOESM1_ESM.pdf]

Additional File 1. Search strategies.

MEDLINE

1. Sedentary Lifestyle/
2. (sedentary or inactiv* or (lack adj2 activity)).tw,kf.
3. ((low adj3 energy expend*) or physical* inactiv*).tw,kf.
4. ((chair or stroller or car or automobile* or auto or motor vehicle* or bus or indoor* or in-door or computer) adj3 time).tw,kf.
5. sitting.tw,kf.
6. Television/ or Video Games/ or Software/ or Videodisc Recording/ or Cartoons as Topic/ or Motion Pictures as Topic/
7. exp Internet/ or exp Computers, handheld/
8. Communications Media/ or Mass Media/
9. (television or screentime or ((screen or computer) adj3 time) or ((watch* or view*) adj2 (dvd* or video*)) or screen media or social media or video gam* or videogam* or computer gam* or electronic gam*).tw,kf.
10. (Smartphone* or ipad or apps or app or mobile applications).tw,kf.
11. screen based entertainment.tw,kf.
12. or/1-11
13. limit 12 to ("infant (1 to 23 months)" or "preschool child (2 to 5 years)")
14. 12 and (pre-school* or preschool* or early childhood).tw,kf.
15. 13 or 14
16. exp obesity/
17. obes*.tw,kf.
18. exp overweight/
19. (overweight or over-weight).tw,kf.
20. exp Body Fat Distribution/
21. exp body composition/
22. (adipos* or fat).mp.
23. waist.mp.
24. Skinfold Thickness/
25. (skinfold* or skin fold*).tw,kf.
26. (body composition* or BMI or body mass index).tw,kf.
27. exp "body weights and measures"/
28. (bio-impedance analysis or BIA).tw,kf.
29. Absorptiometry, Photon/
30. (absorptiomet* or densitomet* or photodensitomet* or DXA or DEXA).tw,kf.
31. Physical Fitness/
32. (physical conditioning or fitness).tw,kf.
33. Range of Motion, Articular/
34. Postural Balance/
35. Metabolic Syndrome X/
36. Insulin Resistance/
37. ((metabolic adj2 syndrome) or syndrome x).tw,kf. and Risk Factors/

38. exp blood glucose/ or exp diabetes mellitus, type 2/
39. exp glucose intolerance/ or glucose tolerance test/
40. Hemoglobin A, Glycosylated/
41. HbA1c.tw,kf.
42. Bone Density/
43. (bone adj2 (density or mass)).tw,kf.
44. (bone mineral content or bone strength).tw,kf.
45. Bone development/
46. Osteogen*.mp.
47. exp Vitamin D Deficiency/
48. exp Cardiovascular Diseases/
49. ((cardiovascular or heart or vascular) adj2 risk$).tw,kf.
50. exp Hypertension/
51. (hypertens* or high blood pressure).tw,kf.
52. exp blood pressure determination/
53. blood pressure/
54. Hypercholesterolemia/
55. exp Hyperlipidemias/
56. (cholesterol or hypercholester* or hyperlipid* or dyslipid*).tw,kf.
57. exp Plagiocephaly/
58. Plagioceph*.tw,kf.
59. Torticollis/
60. exp Psychomotor Performance/
61. motor development.tw,kf.
62. Motor Activity/
63. Gross motor skill*.tw,kf.
64. (fine motor skill* or locomotor control or object control).tw,kf.
65. exp "Wounds and Injuries"/
66. exp Child Development Disorders/
67. Child Development/
68. Developmental milestone*.mp.
69. gd.fs.
70. Child Behavior Disorders/
71. Child Behavior/
72. exp Adaptation, Psychological/
73. (pro-social behav* or prosocial behav* or social behav*).tw,kf.
74. exp *Social Behavior/
75. (behavio?ral conduct or behavio?r* disorder* or conduct disorder*).tw,kf.
76. exp Aggression/
77. Interpersonal Relations/
78. Attention/
79. Attention Deficit Disorder with Hyperactivity/
80. concentrat*.tw,kf.
81. Cognitive develop*.tw,kf.

82. Achievement/
83. (academic achievement or educational achievement).tw,kf.
84. Educational Achievement/
85. School Admission Criteria/
86. (grade-point average or grade point average or GPA).tw,kf.
87. Educational measurement/
88. Educational status/
89. Language Development Disorders/
90. Language Development/
91. Communication/
92. Speech Perception/
93. Verbal Behavior/
94. Vocabulary/
95. exp Self Concept/
96. (self-esteem or self esteem).tw,kf.
97. Self Efficacy/
98. Self-Control/
99. (self regulation or self control).tw,kf.
100. Executive function/
101. exp Memory/
102. Depression/
103. exp Mood Disorders/
104. exp Anxiety Disorders/
105. Affective Symptoms/
106. Stress, Psychological/
107. "Quality of Life"/
108. Temperament/
109. Depression/
110. (depression or depressive).tw,kf.
111. or/16-110
112. 15 and 111
113. limit 112 to (english or french)
114. limit 113 to journal article
115. remove duplicates from 114

EMBASE

1. sedentary lifestyle/
2. (sedentary or inactiv* or (lack adj2 activity)).tw,kw.
3. ((low adj3 energy expend*) or physical* inactiv*).tw,kw.
4. ((chair or stroller or car or automobile* or auto or motor vehicle* or bus or indoor* or in-door or computer) adj3 time).tw,kw.
5. sitting.tw,kw.
6. bed rest.mp.
7. television viewing/ or television/ or exp computer/
8. internet/
9. Social Media/ or Mobile Application/ or Mobile Phone/
10. (screen based entertainment or screen time).tw.
11. (texting or text messag* or app or apps or mobile applications).tw.
12. (smartphone* or smart phone* or cell phone* or mobile phone* or small screen*).tw.
13. (iphone* or ipad* or ipod* or tablet* or laptop*).tw.
14. (social media or Facebook or Youtube or Twitter or Snapchat or Instagram or Pinterest or Skype or Vine).tw.
15. (television or screentime or ((screen or computer) adj3 time) or ((watch* or view*) adj2 (dvd* or video*)) or screen media or social media or video gam* or videogam* or computer gam* or electronic gam*).tw,kw.
16. ((television adj watch*) or tv watch*).tw.
17. or/1-16
18. exp obesity/ or exp adipose tissue/ or body composition/ or body fat/ or body fat distribution/ or skinfold thickness/ or exp body weight/ or body mass/
19. (obes* or overweight or over-weight or adipos* or fat or waist or skin fold* or skinfold* or body composition* or bmi or body mass index).tw,kw.
20. dual energy x ray absorptiometry/ or photon absorptiometry/
21. (bio-impediance analysis or bia or absorptiomet* or densitometr* or photodensitometr* or DXA or DEXA).tw,kw.
22. fitness/ or "range of motion"/ or body equilibrium/
23. (physical conditioning or fitness).tw,kw.
24. metabolic syndrome X/ or insulin resistance/ or non insulin dependent diabetes mellitus/ or glucose blood level/ or glucose intolerance/ or exp glucose tolerance test/ or hemoglobin A1c/
25. ((metabolic adj2 syndrome) or syndrome x).tw,kw. and Risk Factor/
26. HbA1c.tw,kw.
27. bone density/ or bone development/ or (bone adj2 (density or mass)).tw,kw. or (bone mineral content or bone strength).tw,kw. or osteogen*.mp.
28. vitamin d deficiency/ or exp cardiovascular disease/ or ((cardiovascular or heart or vascular) adj2 risk*).tw,kw.
29. exp hypertension/ or exp blood pressure measurement/ or exp blood pressure/ or hyperlipidemia/ or exp hypertriglyceridemia/

30. (hypertens* or high blood pressure or cholesterol or hypercholester* or hyperlipid* or dyslipid*).tw,kw.
31. Plagiocephaly/ or plagiocephal*.tw,kw.
32. torticollis/
33. exp psychomotor performance/
34. exp motor activity/
35. (motor development or gross motor skill* or fine motor skill* or locomotor control or object control).tw,kw.
36. exp wound/
37. exp child development/ or developmental milestone.tw,kw.
38. exp behavior disorder/ or child behavior/ or adaptive behavior/ or exp aggression/ or exp *social behavior/ or human relation/
39. (prosocial behav* or social behav* or ((behavio?ral adj (conduct or disorder*)) or conduct disorder*)).tw,kw.
40. attention deficit disorder/ or exp attention/ or cognitive development/ or achievement/ or exp academic achievement/ or educational status/
41. (concentrat* or academic achievement or educational achievement or grade-point average or grade point average or GPA).tw,kw.
42. exp developmental language disorder/
43. language development/
44. exp interpersonal communication/
45. speech perception/
46. exp verbal behavior/
47. exp developmental language disorder/ or language development/ or exp interpersonal communication/ or speech perception/ or exp verbal behavior/
48. exp self concept/
49. (self esteem or self confidence or self regulation or self control).tw,kw.
50. executive function/ or exp memory/ or exp mood disorder/ or exp anxiety disorder/ or emotional disorder/ or mental stress/ or exp "quality of life"/ or exp temperament/
51. (depression or depressive or anxious* or anxiet*).tw,kw.
52. or/18-51
53. 17 and 52
54. limit 53 to (infant or preschool child <1 to 6 years>)
55. 53 and (pre-school* or preschool* or early childhood).tw,kw.
56. 54 or 55
57. limit 56 to (english or french)
58. limit 57 to embase
59. limit 58 to conference abstract
60. 58 not 59
61. remove duplicates from 60

PsycINFO

1. sedentar*.tw,id.
2. ((low adj3 energy expend*) or physical* inactiv*).tw,id.
3. ((chair or stroller or car or automobile* or auto or motor vehicle* or bus or indoor* or in-door or computer) adj3 time).tw,id.
4. sitting.tw,id.
5. ((television adj watch*) or tv watch* or cartoon*).tw,id.
6. television viewing/
7. (television or screentime or ((screen or computer) adj3 time) or ((watch* or view*) adj2 (dvd* or video*)) or screen media or social media or video gam* or videogam* or computer gam* or electronic gam* or gaming).tw,id.
8. screen based entertainment.tw,id.
9. exp Social Media/
10. exp Mobile Devices/
11. (smartphone* or smart phone* or cell phone* or mobile phone* or small screen*).tw,id.
12. (texting or text messag* or app or apps or mobile applications).tw,id.
13. (iphone* or ipad* or ipod* or tablet* or laptop*).tw,id.
14. bed rest.tw,id.
15. or/1-14
16. exp overweight/ or body weight/ or weight gain/ or weight loss/ or body fat/ or body mass index/ or weight control/ or body size/
17. (obes* or overweight or over-weight or adipos* or fat or waist or skinfold* or skin fold* or body composition or bmi or body mass index).tw,id.
18. (bio-impedance analysis or BIA or absorptiomet* or densitometr* or photodensitometr* or DXA or DEXA).tw,id.
19. physical fitness/ or physical endurance/ or physical strength/ or "range of motion"/
20. (physical conditioning or fitness).tw,id.
21. metabolic syndrome/ or insulin/ or blood suger/ or type 2 diabetes/ or glucose metabolism/ or glucose/
22. ((metabolic adj2 syndrome) or syndrome x).tw,id. and Risk Factors/
23. (glycosylated hemoglobin A or HbA1c).tw,id.
24. (bone adj2 (density or mass or development)).tw,id.
25. (bone mineral content or bone strength or osteogen*).tw,id.
26. bones/
27. exp vitamin deficiency disorders/
28. exp Cardiovascular Disorders/ or exp hypertension/ or cholesterol/ or lipids/
29. ((cardiovascular or heart or vascular) adj2 risk*).tw,id.
30. (hypertens* or high blood pressure or cholesterol or hypercholester* or hyperlipid* or dyslipid*).tw,id.
31. torticollis/
32. plagioceph*.tw,id.
33. exp motor development/ or exp motor processes/
34. (motor development or motor skill* or locomotor control or object control).tw,id.

35. injuries/ or exp head injuries/ or exp spinal cord injuries/ or exp wounds/
36. developmental disabilities/ or exp delayed development/ or exp intellectual development disorder/
37. developmental milestone*.tw,id.
38. behavior disorders/ or exp aggressive behavior/ or exp antisocial behavior/ or exp behavior problems/
39. exp social behavior/
40. interpersonal relationships/ or exp interpersonal interaction/
41. ((behavio?ral adj (conduct or disorder*)) or conduct disorder* or prosocial behav* or social behav*).tw,id.
42. exp attention/ or exp attention deficit disorder/ or concentration/ or distraction/ or concentrat*.tw,id.
43. exp cognitive development/
44. exp academic achievement/ or academic achievement prediction/ or academic aptitude/ or academic failure/ or educational attainment level/ or achievement/ or student admission criteria/ or educational measurement/
45. (cognitive develop* or academic achievement or educational achievement or grade-point average or grade point average or GPA).tw,id.
46. exp Language Development/ or exp Language Disorders/ or exp communication/ or speech perception/ or vocabulary/
47. self-perception/ or exp self-concept/ or self-efficacy/ or self-esteem/ or self-control/ or anger control/ or exp emotional control/ or emotional regulation/ or exp impulse control disorders/ or self-regulation/
48. (self esteem or self regulation or self control).tw,id.
49. exp executive function/ or exp cognitive ability/ or exp memory/ or exp affective disorders/ or exp anxiety disorders/ or psychological stress/ or "quality of life"/ or exp Life Satisfaction/ or exp Well Being/ or personality/
50. (depression or depressive).tw,id.
51. or/16-50
52. 15 and 51
53. limit 15 to (2330 motor processes or 2340 cognitive processes or 2343 learning & memory or 2346 attention or 2800 developmental psychology or 2820 cognitive & perceptual development or 2840 psychosocial & personality development or 2900 social processes & social issues or 3020 group & interpersonal processes or 3040 social perception & cognition or 3211 affective disorders or 3230 behavior disorders & antisocial behavior or 3250 developmental disorders & autism or 3295 cardiovascular disorders)
54. 52 or 53
55. limit 54 to (140 infancy <2 to 23 mo> or 160 preschool age)
56. 54 and (pre-school* or preschool* or early childhood).mp.
57. 55 or 56
58. limit 57 to (english or french)
59. limit 58 to "0100 journal"
60. remove duplicates from 59

CENTRAL

1. (sedentary or inactiv* or (lack adj2 activity)).tw,kw.
2. ((low adj3 energy expend*) or physical* inactiv*).tw,kw.
3. ((chair or stroller or car or automobile* or auto or motor vehicle* or bus or indoor* or in-door or computer) adj3 time).tw,kw.
4. sitting.tw,kw.
5. (television or screentime or ((screen or computer) adj3 time) or ((watch* or view*) adj2 (dvd* or video*)) or screen media or social media or video gam* or videogam* or computer gam* or electronic gam*).tw,kw.
6. (Smartphone* or ipad or apps or app or mobile applications).tw,kw.
7. screen based entertainment.tw,kw.
8. media.tw,kw.
9. or/1-8
10. obese.tw,kw.
11. (overweight or over-weight).tw,kw.
12. (adipos* or fat).mp.
13. waist.mp.
14. (skin folds or skinfold or skin-fold*).tw,kw.
15. (body composition* or BMI or body mass index).tw,kw.
16. (bio-impedance analysis or BIA).tw,kw.
17. (absorptiometery or densitometry or photodensitometry or DXA or DEXA).tw,kw.
18. (physical conditioning or fitness).tw,kw.
19. (((metabolic adj2 syndrome) or syndrome x) and risk).tw,kw.
20. (diabetes or blood glucose or glucose intolerance or glucose intolerance).tw,kw.
21. HbA1c.tw,kw.
22. (bone adj2 (density or mass)).tw,kw.
23. (bone mineral content or bone strength).tw,kw.
24. Osteogen*.mp.
25. vitamin d defic*.tw,kw.
26. ((cardiovascular or heart or vascular) adj2 (risk$ or disease)).tw,kw.
27. (hypertens* or high blood pressure).tw,kw.
28. (cholesterol or hypercholester* or hyperlipid* or dyslipid*).tw,kw.
29. Plagioceph*.tw,kw.
30. torticollis.tw,kw.
31. motor development.tw,kw.
32. Gross motor skill*.tw,kw.
33. (fine motor skill* or locomotor control or object control).tw,kw.
34. (flexibility or (range adj2 motion)).tw,kw.
35. balance.tw,kw.
36. Injur*.tw,kw.
37. Developmental milestone*.mp.
38. (social or pro-social or prosocial).tw,kw.

39. (behavio?ral conduct or behavio?ral conduct or behavio?r* disorder* or conduct disorder*?).tw,kw.
40. concentrat*.tw,kw.
41. attention.tw,kw.
42. Cognitive develop*.tw,kw.
43. achievement.tw,kw.
44. (grade-point average or grade point average or GPA).tw,kw.
45. (language or communication or verbal or vocabulary).tw,kw.
46. (self-esteem or self esteem).tw,kw.
47. (self regulation or self control).tw,kw.
48. (depression or depressive).tw,kw.
49. (anxiety or anxious).tw,kw.
50. (quality adj2 life).tw,kw.
51. (memory or executive function).tw,kw.
52. psychosocial.tw,kw.
53. 9 and (or/10-52)
54. 53 and (infant* or child* or preschool* or pre-school*).tw,kw.
55. limit 54 to medline records
56. limit 54 to embase records
57. 54 not (55 or 56)
58. remove duplicates from 57

Pubmed

((sedentary OR inactive OR inactivity) and (Infant or child* or preschool or pre-school) and (pubstatusaheadofprint or publisher[sb] or pubmednotmedline[sb]))

SPORTdiscus

S14	s13 with Limiters - Language: English, French; Publication Type: Academic Journal ; Document Type: Article
S13	S11 and S12
S12	Infan* or child* or pre-school or preschool
S11	S1 or S2 OR S3 OR S4 OR S5 OR S6 OR S7 OR S8 OR S9 OR S10
S10	(Smartphone* or ipad or apps or app or mobile applications) or screen based entertainment
S9	(television N3 time or screentime or screen N3 time or computer N3 time or (watch* N2 dvd or watch N2 video* or view* N2 dvd* or view N2 video*)) or screen media or social media or video gam* or videogam* or computer gam* or electronic gam*
S8	chair N3 time or stroller N3 time or car N3 time or automobile* N3 time or auto N3 time or motor vehicle* N3 time or bus N3 time or indoor* N3 time or in-door N3 time or computer N3 time
S7	sitting
S6	((sedentary or inactiv* or (lack N2 activity)))
S5	((low N3 energy expend*) or physical* inactiv*)
S4	DE "MASS media"
S3	DE "INTERNET" OR DE "ELECTRONIC games" OR DE "INTERNET games" OR DE "MULTIPLAYER games" OR DE "VIDEO games")
S2	DE "SEDENTARY behavior"
S1	DE "SEDENTARY behavior in children"

Communication Source

(sedentary OR inactive OR inactivity) AND (infant or child* or preschool* or pre-school*)

Communications and Mass Media Collection

Keyword (sedentary Or inactive Or inactivity) AndKeyword (infant Or child* Or preschool* Or pre-school*) AndKeyword (health* Or development* Or obes* Or fitness Or self esteem Or disease* Or diabetes) LIMITS:Peer-Reviewed
