# Supplementary material for: Systematic review of the relationships between sedentary behaviour and health indicators in the early years (0–4 years)
Source: BMC Public Health. 2017 Nov 20;17(Suppl 5):868. doi: 10.1186/s12889-017-4849-8 (PMC5773886; doi:10.1186/s12889-017-4849-8)
Supplement: Supplementary file 2 — Supplementary Tables S1-S7. (PDF 1166 kb) [file 12889_2017_4849_MOESM2_ESM.pdf]

Additional File 2: Supplementary Tables

Table S1. Summary of findings for adiposity outcomes

Reference; Country; Larger Study/Cohort Name	Study Design	Sample	Exposure	Outcome	Main Findings	Covariates included in model (if applicable)
Study Design: RCT						
Yilmaz et al. 2014 [1]; Turkey	Randomized Controlled Trial (RCT)	N = 412<br><br>Intervention, n = 211;<br>Control, n = 201<br><br>Mean age:<br>Intervention, 3.52 yr<br>Control, 3.49 yr<br>Pre-schoolers	**Intervention:** 3 printed materials and interactive CDs and one counselling call, intending to decrease screen time.<br>**Control:** Usual care; unaware of counselling interventions.<br><br>**Screen time** (min/day) was assessed by parent-report 1-week time-record diary (unpublished format).<br><br>The intervention was 8 weeks in duration, with follow-up at 2, 6 and 9 months post-intervention.	**BMI z-score** calculated from "recorded" heights and weights (unclear if these were objectively measured or proxy-reported).	**Screen time** was significantly lower in the **intervention** vs **control** group at 2, 6 and 9 month follow-up post-intervention (mean ± SD: 2 month: 39.48 ± 16.36 vs 86.64 ± 21.63 min/day; 6 month: 24.72 ± 4.45 vs 84.95 ± 14.77 min/day; 9 month: 21.15 ± 6.12 vs 93.96 ± 18.84 min/day; all $p < 0.001$).<br><br>**BMI z-scores** were not different between the **intervention** and **control** groups at baseline (mean ± SD: intervention, -0.19 ± 1.12; control, -0.22 ± 0.81, $p = 0.375$) or 9-month follow-up (mean ± SD: intervention, -0.13 ± 1.05; control, -0.15 ± 0.95, $p = 0.80$).<br><br>**BMI z-scores** increased in both groups (no data provided).	N/A

Study Design: Longitudinal						
Sijtsma et al. 2013 [2]; Netherlands<br><br>Groningen Expert Center for Kids with Obesity (GECKO) Drenthe birth cohort	Longitudinal [~15 mo (1.25 yr) follow-up]	N = 1276<br><br>Approximate age:<br>T1, 9 mo (0.75 yr); Infants<br><br>T2, 24 mo (2 yr); Toddlers	**Time in baby seats** (hr/day; e.g., car seat or child seat) assessed by parent-report questionnaire (unpublished).	**Weight-for-height** (z-score) calculated from objectively measured weight and height.<br><br>**Weight-for-age** (z-score) calculated using objectively measured weight.<br><br>**Waist circumference-for-age** (z-score) calculating using objectively measured waist circumference (measured at the mid-point between the lower costal margin and the level of the anterior superior iliac spine).	**Time in baby seats** at age ~9 mo was not associated with **weight-for-height** (Never, -0.02, SD = 1.05; <1 hr, 0.02, SD = 0.98, ≥1 hr, -0.06, SD = 1.03; p > 0.05) or **weight-for-age** (Never, 0.12, SD = 1.01; <1 hr, 0.04, SD = 1.00; ≥1 hr, -0.11, SD = 0.92; p > 0.05) at ~24 mo.<br><br>**Time in baby seats** at age ~9 mo was favourably associated with **waist circumference-for-age** at 24 mo (Never, 0.17, SD = 0.91; <1 hr, 0.04, SD = 1.00; ≥1 hr, -0.19, SD = 0.90; p < 0.01; <1 hr vs ≥1 hr, p < 0.05), and with **change in waist circumference-for-age** from 9 to 24 mo (Never, 0.00, SD = 1.26; <1 hr, 0.03, SD = 1.15; ≥1 hr, -0.22, SD = 1.04; p < 0.05; <1 hr vs ≥1 hr, p < 0.05).<br><br>**Time in baby seats** at age ~9 mo was unfavourably associated with **change in weight-for-height** (Never, -0.34, SD = 0.63; <1 hr, -0.02, SD = 0.77; ≥1 hr, 0.00, SD = 0.71; p < 0.05; never vs <1 hr and never	N/A

					vs ≥1 hr, $p < 0.05$) and **change in weight-for-age** (Never, -0.30, SD = 0.82; <1 hr, 0.00, SD = 0.78; ≥1 hr, -0.11, SD = 0.74, $p < 0.05$) from 9 mo to 24 mo.	
Gooze et al. 2011 [3]; USA; Early Childhood Longitudinal Study, Birth Cohort (ECLS-B)	Longitudinal (~3.5 yr follow-up)	N ≈ 6650 (rounded to the nearest 50)<br><br>Approximate age:<br>T1, 24 mo (2 yr)<br>Toddlers<br><br>T2, 5.5 yr<br>School-age	**Screen time** (≤ or >2 hr/day; TV and videos) assessed by parent-report questionnaire (unpublished).	**Weight status** (2000 US growth reference cut-points [4]; categories: BMI-for-age ≥95th percentile, obese; <95th percentile, non-obese) determined from BMI (calculated from objectively measured height and weight).	The prevalence of **obesity** at age ~5.5 yr was significantly greater in children with **>2 hr/day screen time** (20.8%, 95% CI: 18.3, 23.2) compared to those with **≤2 hr/day screen time** (15.5%, 95% CI: 14.1, 16.9) at age ~2 yr ($p<0.001$).	N/A
Griffiths et al. 2010 [5]; England, Wales, Scotland, Northern Ireland; Millennium Cohort Study	Longitudinal (~2 yr follow-up)	N = 11652<br><br>Approximate age:<br>T1, 3 yr<br>Toddlers<br><br>T2, 5 yr<br>School-age	**TV time** (hr/day; categories: <1 hr/day, ≥1 to <3 hr/day, ≥3 hr/day) assessed by parent-report questionnaire (unpublished).	**Weight gain rate** from ages 3 to 5 yr (categories: “rapid weight gain”, children in the top quarter of the weight gain z-score distribution; “normal weight gain”, children below the top quarter of the weight gain z-score distribution) calculated from objectively measured height and weight.	**TV time** at age ~3 yr was not associated with **rapid weight gain** (compared to <1 hr/day: ≥1 to <3 hr/day, OR = 1.03, 95% CI: 0.92, 1.17; ≥3 hr/day, OR = 1.17, 95% CI: 1.00, 1.38; $p = 0.10$) from ages ~3 to 5 yr.	N/A
Schmidt et al. 2009 [6]; USA<br><br>Project Viva	Longitudinal (~2.5 yr follow-up)	N = 872<br><br>T1, Age Range: 6 mo to 2 yr<br>Toddlers and Pre-schoolers<br><br>T2, ~Age: 3 yr<br>Pre-schoolers	**TV time** (hr/day) assessed by parent-report questionnaire (unpublished) at ages 6 mo, 1 yr and 2 yr, and as the weighted average of TV exposure from birth and 2 yr [adapted from questionnaire used in the National Longitudinal Survey of Children and	**BMI z-score** (calculated from objectively measured height and weight).	**TV time** from birth to 2 yr was unfavourably associated with **BMI z-score** at age 3 yr (mean BMI z-score by TV time: 0 to 0.5 hr/day, mean = 0.20, SD = 1.04; 0.5 to < 1 hr/day, mean = 0.40, SD = 0.89; 1 to <2 hr/day, mean = 0.58, SD = 0.97; ≥2 hr/day, mean = 0.58, SD =	N/A

			Youth study (NLSY)].		1.14; p = 0.0001).	
Fuller-Tyszkiewicz et al. 2012 [7]; Australia<br><br>Longitudinal Study of Australian Children (LSAC)	Longitudinal (~2 yr follow-up)	N = 4724<br><br>Mean age:<br>T1, 2.29 yr<br>Toddlers<br><br>T2, 4.25 yr<br>Pre-schoolers<br><br>T3, 6.42 yr<br>School-age	**TV time** (min/week) assessed by parent-report interview (unpublished format).	**BMI** (kg/m^2) calculated from objectively measured height and weight.	**TV time** at age 2.29 yr was unfavourably associated with **BMI** at age 4.25 yr (*r* = 0.03, p < 0.05) and age 6.42 yr (*r* = 0.40, p < 0.05.<br><br>**TV time** at age 4.25 yr was unfavourably associated with **BMI** at age 6.42 yr (*r* = 0.04, p < 0.05).	N/A
Fitzpatrick et al. 2012 [8]; Canada<br><br>Quebec Longitudinal Study of Child Development (QLSCD)	Longitudinal (~8 yr follow-up)	N = 1314<br><br>T1, ~Age: 29 mo (2.4 yr)<br>Toddlers<br><br>T2, ~Age: 53 mo (4.4 yr)<br>Pre-schoolers<br><br>T3, Mean age: 121.8 mo (10.15 yr; Grade 4)<br>School-age	**TV time** (hr/week) at age 29 mo assessed by parent report questionnaire (unpublished)<br><br>**Change in TV time** (hr/week) between age 29 mo and age 53 mo.	**Waist circumference** (cm) measured objectively (at the midway points between the iliac crest and<br>the lowest rib on children's left and right sides).	**TV time** at age ~29 mo was not associated with **waist circumference** at 121.8 mo (B = 0.009, 95% CI: -0.005, 0.073, p > 0.05).<br><br>**Change in TV time** from ~29 to ~53 mo was unfavourably associated with **waist circumference** at 121.8 mo (B = 0.047, 95% CI: 0.001, 0.094, p < 0.05).	Maternal BMI, immigration status, level of education, child age in mo in Grade 2, weight status in Grade 2.
Pagani et al. 2010 [9]; Canada<br><br>QLSCD	Longitudinal (~8 yr follow-up)	N = 1314<br><br>T1, ~Age:<br>29 mo (2.4 yr)<br>Toddlers<br><br>T2, ~Age: 53 mo (4.4 yr)<br>Pre-schoolers<br><br>T3, Grade 4 (in	**TV time** (hr/week) at age 29 mo assessed by parent-report questionnaire (unpublished).<br><br>**Change in TV time** (hr/week) from age 29 mo to 53 mo.	**BMI** (kg/m^2) calculated from objectively measured height and weight.	**TV time** at age 29 months was unfavourably associated with **BMI** in Grade 4 (age ~9-10 yr; β = 0.05, SE = 0.02; B = 0.05, 95% CI: 0.01, 0.09; p < 0.01).<br><br>The **change in TV time** from age 29 mo to 53 mo was unfavourably	**TV time:** Change in TV time, concurrent TV time, sex, temperament, cognitive ability, impulsivity, emotional distress, physical aggression, *hours of sleep*, maternal education, family makeup, family functioning.

		Grade 4 in Quebec, children are aged 9 to 10; School-age)			associated with **BMI** in Grade 4 (age ~9-10 yr; β = 0.03, SE = 0.01; B = 0.03, 95% CI: 0.01, 0.05; p < 0.01).	**Change in TV time:** Same as above, except inclusion of "baseline TV time" instead of "change in TV time".
Reilly et al. 2005 [10]; England<br><br>Avon Longitudinal Study of Parents and Children (ALSPAC)	Longitudinal (~4 yr follow-up)	N = 5493<br><br>Approximate age at exposure measurement: 38 mo (3.2 yr)<br>Pre-schoolers<br><br>Median age at follow-up: 7.6 yr (range: 6.9 to 8.5 yr)<br>School-age	**TV time** (hr/week) and **time spent in the car** on weekdays and weekend days (hr/day) assessed by parent-report questionnaire (unpublished).	**Weight status** (United Kingdom reference standards in 1990; categories: categories: obese, non-obese) determined from BMI (calculated from objectively measured height and weight).	There was a linear dose-response unfavourable relationship between **TV time** at age 38 mo and odds of **obese weight status** at age ~7 yr (*unadjusted:* compared to ≤4 hr/week: 4.1-8 hr/week: OR=1.66, 95% CI: 1.28, 2.17; >8 hr/week: OR=2.10, 95% CI: 1.60, 2.77; p < 0.001; *adjusted:* compared to ≤4 hr/week: 4.1-8 hr/week: OR=1.37, 95% CI: 1.02, 1.83; >8 hr/week: OR=1.55, 95% CI: 1.13, 2.12; p < 0.010).<br><br>**Time spent in the car on weekdays** at age ~38 months was not associated with **obese weight status** at age ~7 yr (*unadjusted:* compared to none: <1 hr/week: OR = 1.19, 95% CI: 0.84, 1.69; ≥1 hr/week: OR = 1.37, 95% CI: 0.90, 2.07; p = 0.34).<br><br>**Time spent in the car on weekend days** at age ~38 months was not associated	Birth weight, maternal smoking, infant feeding, parental obesity, *sleep*, dietary patterns, maternal education, energy intake at age 3 years, sex.

					with **obese weight status** at age ~7 yr (*unadjusted:* compared to none: <1 hr/week: OR = 0.62, 95% CI: 0.40, 0.95; ≥1 hr/week: OR = 0.64, 95% CI: 0.41, 0.99; p = 0.090).	
Leary et al. 2015 [11]; England<br><br>ALSPAC	Longitudinal (~12 yr follow-up)	N = 4750<br><br>Approximate ages: T1, "38 month [3.2 yr] clinic visit" (TV and car measures) or "58 month [4.8 yr] clinic visit" (computer game exposure)<br>Pre-schoolers<br><br>T2, 15.5 yr<br>School-age	**Time spent in the car** (hr/week) and **TV time** (hr/week) at age 38 mo assessed by parent-report questionnaire (unpublished).<br><br>**Frequency of playing computer games** (categories: rarely/not at all, once a month, once a week, 2-7 times/week) at age 57 mo assessed by parent-report questionnaire (unpublished)	**Total fat mass** (SD score) and **lean mass** (SD score) measured objectively by DXA.<br><br>**BMI** (SD score) calculated from objectively measured height and weight.	**Time spent in the car** at age ~38 mo was not significantly associated with **fat mass** (compared to <4 hr/week: 4-5 hr/week, β = -0.02, 95% CI: -0.10, 0.06; >5 hr/week, β = 0.05, 95% CI: -0.06, 0.15, p = 0.50), **lean mass** (compared to <4 hr/week: 4-5 hr/week, β = -0.005, 95% CI: -0.05, 0.04; >5 hr/week, β = 0.02, 95% CI: -0.04, 0.08, p = 0.60), or **BMI** (compared to <4 hr/week: 4-5 hr/week, β = -0.04, 95% CI: -0.13, 0.05; >5 hr/week, β = 0.09, 95% CI: -0.03, 0.20, p = 0.30) at age ~15 yr.<br><br>**TV time** at age ~38 mo was unfavourably associated with **fat mass** (compared to ≤4 hr/week: 4.1-8 hr/week, β = 0.04, 95% CI: -0.04; >8 hr/week, β = 0.11, 95% CI: 0.02, 0.21, p = 0.01), but not **lean mass**	**Time spent in the car:** Gender, age at time of 15-yr clinic visit, height, parental factors, social factors, birthweight and gestation (plus energy intake at 38 mo for dietary patterns), maternal smoking, paternal smoking, breastfeeding, age at introduction to solids, time watching TV at 38 mo, frequency playing computer games at 57 mo.<br><br>**TV time:** Same as above, except instead of "time watching TV at 38 mo" inclusion of "time spent in the car at 38 mo".<br><br>**Frequency of playing computer games:** Same as for "time spent in the car", except with the addition of "time spent in the car at 38 mo".

						(compared to ≤4 hr/week: 4.1-8 hr/week, β = -0.01, 95% CI: -0.06, 0.03; >8 hr/week, β = -0.03, 95% CI: -0.08, 0.02, p = 0.30) or **BMI** (compared to ≤4 hr/week: 4.1-8 hr/week, β = 0.01, 95% CI: -0.08, 0.10; >8 hr/week, β = 0.08, 95% CI: -0.03, 0.18, p = 0.10), at age ~15 yr.<br><br>**Frequency of playing computer games** at age ~57 mo was not significantly associated with **fat mass** (compared to rarely/not at all: once a month, β = -0.03, 95% CI: -0.12, 0.06; once a week, β = 0.05, 95% CI: -0.03, 0.13; 2-7 times/week, β = -0.01, 95% CI: -0.10, 0.08, p = 0.70), **lean mass** (compared to rarely/not at all: once a month, β = 0.005, 95% CI: -0.05, 0.06; once a week, β = 0.03, 95% CI: -0.02, 0.07; 2-7 times/week, β = 0.05, 95% CI: -0.002, 0.11, p = 0.05), or **BMI** (compared to rarely/not at all: once a month, β = -0.005, 95% CI: -0.10, 0.09; once a week, β = 0.10, 95% CI: 0.003, 0.19; 2-7 times/week, β = 0.07, 95% CI: -0.03, 0.17, p =	

					0.06) at age ~15 yr.	
Flores and Lin 2013[12]; USA<br><br>Early Childhood Longitudinal Study-Birth Cohort (ECLS-B)	Longitudinal (~1 to 2 yr follow-up)	N = 6800<br><br>Approximate ages: Baseline, "preschool age" (3 to 4 yr) Pre-schoolers<br><br>Follow-up, "kindergarten entry" School-age	**TV time** (hr/day) and **time watching DVDs** (hr/day) on weekdays assessed by parent-report questionnaire (unpublished).	**Weight status** (Centers for Disease Control and Prevention (CDC) cut-points; categories: not overweight, overweight) determined from BMI (calculated from objectively measured height and weight).	Children with **overweight** status had greater **TV time** (2.5 hr/day) at preschool age than children **without overweight** status (2.2 hr/day) at entry to kindergarten (p = 0.03).<br><br>**Time watching DVDs** on weekdays at preschool age was unfavourably associated with **overweight status** at entry to kindergarten (compared to not overweight, for each additional hour of time watching DVDs, OR = 1.1, 95% CI: 1.01, 1.14).	Pacific islander race/ethnicity, non-English primary language spoken at home, and number of children in the household.
Olafsdottir et al. 2014 [13]; Estonia, Sweden, Germany, Belgium, Hungary, Italy, Spain and Cyprus<br><br>Identification and prevention of dietary and lifestyle-induced health effects in children and infants (IDEFICS)	Longitudinal (2 yr follow-up)	N = unclear (only the total study sample is reported, but this includes other age groups that are not of interest)<br><br>Age range at baseline: 2 to < 6 yr Toddlers and Pre-schoolers	**TV time** (hr/day; TV, video, DVD) and **screen time** (hr/day; TV, video, DVD, computer) assessed by parent-report questionnaire (unpublished).	**BMI** (kg/m^2) calculated from objectively measured height and weight.<br><br>**Waist-to-height ratio** (WHtR; no units, ratio) calculated from objectively measured height and waist circumference (measured at the midpoint between the iliac crest and the lower costal border or 10th rib).<br><br>**Weight status** (IOTF cut-points [14]; categories: normal weight or	**TV time** at baseline was unfavourably associated with **% change in BMI** from baseline to 2-yr follow-up (odds of being in highest quintile of % change in BMI for each hr/day of TV: OR = 1.23, 95% CI: 1.08, 1.40, p < 0.01).<br><br>**TV time** at baseline was unfavourably associated with **% change in WHtR** from baseline to 2-yr follow-up (odds of being in highest quintile of % change in WHtR for each	Age, sex, parental education, intervention and study centre.

				underweight, overweight or obese) determined from BMI (calculated from objectively measured height and weight).	hr/day of TV: OR = 1.32, 95% CI: 1.14, 1.52, p < 0.001).<br><br>**Screen time** at baseline was unfavourably associated with **% change in BMI** from baseline to 2-yr follow-up (odds of being in highest quintile of % change in BMI for each hr/day of screen time: OR = 1.15, 95% CI: 1.04, 1.28, p < 0.01).<br><br>**Screen time** at baseline was unfavourably associated with **% change in WHtR** from baseline to 2-yr follow-up (odds of being in highest quintile of % change in WHtR for each hr/day of screen time: OR = 1.22, 95% CI: 1.09, 1.36, p < 0.01).	
Wheaton et al. 2015 [15]; Australia<br><br>LSAC	Longitudinal (~ 6 yr follow-up)	N = 4169<br><br>Mean age:<br>T1, 4.80 yr;<br>Pre-schoolers<br><br>T2, 6.87 yr;<br>T3, 8.85 yr;<br>T4, 10.89 yr<br>School-age	**TV time** (hr/day) and **computer time** (hr/day) on weekdays and weekend days assessed by parent-report questionnaire (unpublished).	**Change in weight status** [World Health Organization (WHO) cut-points; overweight (OW; >+1 SD), obese (OB; >+2 SD); categories: normal (NW) to OW/OB, OW/OB to NW, remained NW, OW/OB] determined from BMI z-score (calculated from objectively measured height and weight).	**Weekday TV time** at age ~5 yr was not associated with **change in weight status** from **5 to ~7 yr** (compared to remained NW: NW to OW/OB, RRR = 0.84, SE = 0.19, p = 0.45; remained OW/OB, RRR = 1.02, SE = 0.15, p = 0.91; OW/OB to NW, RRR = 0.99, SE = 0.21, p = 0.34), **5 to ~9 yr** (compared to remained	**Weekday TV time:** Fruit and vegetable intake, high-fat foods, sugar-sweetened beverages; computer use week and weekend day; TV use weekend day; sex; ethnicity; socioeconomic position; mother and father BMI.<br><br>**Weekend TV time:** Same as above, except “TV use

					NW: NW to OW/OB, RRR = 1.01, SE = 0.18, p = 0.95; remained OW/OB, RRR = 1.18, SE = 0.18, p = 0.26; OW/OB to NW, RRR = 0.97, SE = 0.17, p = 0.87), or **5 to ~11 yr** (compared to remained NW: NW to OW/OB, RRR = 0.96, SE = 0.18, p = 0.82; remained OW/OB, RRR = 1.10, SE = 0.18, p = 0.54; OW/OB to NW, RRR = 1.08, SE = 0.17, p = 0.65).<br><br>**Weekend TV time** at age ~5 yr was not associated with **change in weight status** from **5 to ~ 7 yr** (compared to remained NW: NW to OW/OB, RRR = 1.27, SE = 0.26, p = 0.24; remained OW/OB, RRR = 1.21, SE = 0.15, p = 0.14; OW/OB to NW, RRR = 1.05, SE = 0.15, p = 0.76), or 5 to **~11 yr** (compared to remained NW: NW to OW/OB, RRR = 1.17, SE = 0.17, p = 0.28; remained OW/OB, RRR = 1.12, SE = 0.15, p = 0.39; OW/OB to NW, RRR = 1.19, SE = 0.18, p = 0.25), but was unfavourably associated with likelihood of changing from **normal**	weekday" instead of "TV use weekend day".<br><br>**Weekday computer time:** Same as for "weekday TV time", except "TV use weekday" instead of "computer use weekday".<br><br>**Weekend computer time:** Same as for "weekday TV time", except "TV use weekday" instead of "computer use weekend day".

					**weight** to **overweight/obese status** at age **~9 yr** (compared to remained NW: NW to OW/OB, RRR = 1.39, SE = 0.23, $p < 0.05$; remained OW/OB, RRR = 1.24, SE = 0.16, p = 0.87; OW/OB to NW, RRR = 1.11, SE = 0.17, p = 0.47).<br><br>**Weekday computer time** at age ~5 yr was not associated with **change in weight status** from **5 to ~ 7 yr** (compared to remained NW: NW to OW/OB, RRR = 1.17, SE = 0.21, p = 0.38; remained OW/OB, RRR = 1.04, SE = 0.12, p = 0.74; OW/OB to NW, RRR = 0.83, SE = 0.12, p = 0.19), **5 to ~9 yr** (compared to remained NW: NW to OW/OB, RRR = 0.94, SE = 0.13, p = 0.66; remained OW/OB, RRR = 1.00, SE = 0.12, p = 0.98; OW/OB to NW, RRR = 0.78, SE = 0.12, p = 0.10), or **5 to ~11 yr** (compared to remained NW: NW to OW/OB, RRR = 0.95, SE = 0.13, p = 0.87; remained OW/OB, RRR = 1.06, SE = 0.13, p = 0.70; OW/OB to NW, RRR = 0.78, SE = 0.11, p = 0.09).	

					**Weekend computer time** at age ~ 5 yr was not associated with **change in weight status** from **5 to ~7 yr** (compared to remained NW: NW to OW/OB, RRR = 0.87, SE = 0.15, p = 0.43; remained OW/OB, RRR = 1.07, SE = 0.11, p = 0.53; OW/OB to NW, RRR = 0.99, SE = 0.13, p = 0.96), **5 to ~9 yr** (compared to remained NW: NW to OW/OB, RRR = 0.94, SE = 0.12, p = 0.63; remained OW/OB, RRR = 1.09, SE = 0.11, p = 0.40; OW/OB to NW, RRR = 1.01, SE = 0.14, p = 0.97), or **5 to ~11 yr** (compared to remained NW: NW to OW/OB , RRR = 0.93, SE = 0.11, p = 0.56; remained OW/OB, RRR = 1.04, SE = 0.12, p = 0.73; OW/OB to NW, RRR = 1.01, SE = 0.13, p = 0.92).	
De Coen et al. 2013[16]; Belgium	Longitudinal [~30 mo (2.5 yr) follow-up]	N = 538<br><br>Mean age, baseline: 4.95 yr<br>Pre-schoolers<br><br>Follow-up at "18 mo" or "30 mo" after baseline (mean age	**Screen time** (hr/day; TV, computer, DVD, etc.) on weekdays and weekend days assessed by parent-report questionnaire (unpublished).	**Weight status** [Flemish reference data; categories: normal weight, overweight (>+1 SD)] determined from BMI z-scores (calculated from objectively measured height and weight).	**Weekday screen time** at age ~5 yr was not associated with having **overweight weight status** at ~18 mo follow-up (compared to ≤1 hr/day, OR = 1.91, 95% CI: 0.91, 3.06, p = 0.08) or ~30 mo (compared to ≤1 hr/day,	Weight status at baseline, living situation, number of children in the family, maternal education level, paternal education level, language spoken at home, maternal professional status, paternal professional status, soft

		not reported)<br>School-age			OR = 2.07, 95% CI: 0.98, 4.25, p = 0.06).<br><br>**Weekend screen time** at age ~5 yr was not associated with having **overweight weight status** at ~18 mo follow-up (compared to ≤2 hr/day, OR = 1.07, 95% CI: 0.49, 2.34, p > 0.10) or ~30 mo (compared to ≤2 hr/day, OR = 1.00, 95% CI: 0.39, 2.56, p > 0.10).	drinks consumption, water consumption, milk products consumption, fruit consumption, vegetable consumption, sweet and savory snacks consumption, *physical activity at home*, *structured physical activity*, screen time on weekends *or* weekdays.
Study Design:<br>Case-Control						
Koleilat et al. 2012 [17]; USA	Case-Control	N = 556<br>Cases (overweight), n = 260<br>Controls (healthy weight), n = 263<br><br>Age Range: 3-4 yr<br>Pre-schoolers	**TV time** (hr/day) assessed by parent-report interview (unpublished format).	**Weight status** (CDC cut-points; categories: overweight >95th percentile, healthy weight 25-75th percentile) determined from BMI (calculated from objectively measured height and weight).	The proportion of children with <1hr/day or ≥1 hr/day **TV time** was not different between the **healthy weight** and **overweight** groups (<1 hr/day: healthy weight, 20.27%; overweight, 14.23%; ≥1 hr/day: healthy weight, 79.73%; overweight, 85.77%; p > 0.05).<br><br>**Watching TV** for ≥1 hr/day was unfavourably associated with having **overweight status** (OR = 1.71, 95% CI: 1.07, 2.75, p = 0.02).	Gender, race, maternal BMI, maternal education, maternal occupation, number of hours in child care, number of days in child care, quality of the home environment.
Kain and Andrade 1999 [18]; Chile	Case-Control	N=686<br><br>Cases (Obese),	**TV time** (hr/day) and **total sedentary time** (hr/day; activities with	**Weight status** (WHO cut-points; categories: obese, weight/height ≥2 SD; non-	**TV time** was not different between children with **obese weight status**	N/A

		n=237<br>Control, n=449<br><br>Approximate age: 4 yr<br>Pre-schoolers	energy expenditure < 1.2-1.4 METs using previously published reference values for activities) assessed by one-day parent-recall of usual activities during a week day (unpublished format).	obese, weight/height -1 to 1 SD) determined from the ratio of objectively measured height to weight.	(*boys:* 3.2 hr/day; *girls:* 3.3 hr/day) and **non-obese weight status** (*boys:* 3.4 hr/day; *girls:* 3.2 hr/day) (both p > 0.05).<br><br>**Total sedentary time** was not different between children with **obese weight status** (*boys:* 7.8 hr/day; *girls:* 8.0 hr/day) and **non-obese weight status** (*boys:* 7.9 hr/day; *girls:* 8.1 hr/day) (both p > 0.05).	
Study Design:<br>Cross-sectional						
Sijtsma et al. 2013[2]; Netherlands<br><br>Groningen Expert Center for Kids with Obesity (GECKO) Drenthe birth cohort	Cross-sectional	N = 1722<br><br>Approximate age: 9 mo (0.75 yr)<br>Infants	**Time in baby seats** (hr/day; categories: never, <1 hr/day, ≥1 hr/day; e.g., car seat or child seat) assessed by parent-report questionnaire (unpublished).	**Weight-for-height** (z-score) calculated from objectively measured weight and height.<br><br>**Weight-for-age** (z-score) calculated using objectively measured weight.<br><br>**Waist circumference-for-age** (z-score) calculating using objectively measured waist circumference (measured at the mid-point between the lower costal margin and the level of the anterior superior iliac spine).	**Time in baby seats** was not associated with **weight-for-height** (Never, 0.28, SD = 1.06; <1 hr, 0.04, SD = 0.98; ≥1 hr, 0.02, SD = 1.01; p > 0.05), **weight-for age** (Never, 0.31, SD = 1.05; <1 hr, 0.03, SD = 0.98; ≥1 hr, 0.07, SD = 0.98; p > 0.05) or **waist circumference-for-age** (Never, 0.10, SD = 1.09; <1 hr, -0.02, SD = 1.00; ≥1 hr, 0.07, SD = 0.97; p > 0.05).	N/A

Wen et al. 2014 [19]; Australia<br><br>Healthy Beginnings Trial	Cross-sectional	N = 242<br><br>Approximate age: 2 yr<br>Toddlers	**TV time** (hr/week) assessed by parent-report questionnaire [20].	**BMI** (kg/m^2) calculated from objectively measured height and weight.<br><br>**Weight status** (categories: normal weight, overweight, obese) determined from BMI and IOTF cut-points from [14].	**TV time** was unfavourably associated with **BMI** (unadjusted: β = 0.08, 95% CI: 0.02, 0.14; adjusted: β = 0.09, 95% CI: 0.03, 0.15, p = 0.003) and having **overweight/obese weight status** in unadjusted (OR = 1.1, 95% CI: 1.01, 1.21) but not adjusted (data not reported) analyses.	Breastfeeding, birth weight, mothers' weight status, mothers' country of birth, marital status, education level.
Johansson et al. 2015 [21]; Sweden<br><br>Early Stockholm Obesity Prevention Project (Early STOPP)	Cross-sectional	N = 123<br><br>Mean age: 2.03 yr<br>Toddlers	**Total sedentary time** min/day; ≤89 counts/5 sec epoch) measured objectively by accelerometer (Actigraph, GT3X+ worn on the wrist).<br><br>**Number of 30 min bouts of sedentary behaviour** and **total time in 30 min bouts of sedentary behaviour** (number and min/day; 30 min of ≤89 counts/5 sec with a 1 min interruption of >89 counts/5 sec allowed).	**Weight status** (IOTF cut-[14]; categories: normal weight, overweight/obese) determined from BMI (calculated from objectively measured height and weight).	**Total sedentary time** was not different between children with **normal** (432, SD = 48 min/day) and **overweight** (422, SD = 40 min/day) **weight status** (p = 0.42).<br><br>The **number of 30 min bouts of sedentary behaviour** was not different between children with **normal** (2.2, SD = 1.3 bouts) and **overweight** (2.4, SD = 0.8 bouts) **weight status** (p = 0.73), nor was the **total time in 30 min bouts of sedentary behaviour** (normal weight: 155, SE = 83 min; overweight: 146, SD = 51 min; p = 0.63).	
Wijtzes et al. 2013 [22]; Netherlands<br><br>Generation R Study	Cross-sectional	N = 347<br><br>Approximate age: 25 mo (2.1 yr)<br>Toddlers	**Total sedentary time** (% of monitored time; measured on 1 weekday and 1 weekend day; ≤301 counts/15 sec epoch)	**BMI z-score** (no units) calculated from objectively measured height and weight.	**Total sedentary time** was not associated with **BMI z-score** (β = 0.10, 95% CI: -0.4 - 0.6, p > 0.05).	N/A

			measured objectively by accelerometer (Actigraph, AM-7164).			
Fuller-Tyszkiewicz et al. 2012 [7]; Australia<br><br>LSAC	Cross-sectional	N = 4724<br><br>Mean age:<br>T1, 2.29 yr<br>Toddlers<br><br>T2, 4.25 yr<br>Pre-schoolers	**TV time** (min/week) assessed by parent-report interview (unpublished format).	**BMI** (kg/m^2) calculated from objectively measured height and weight.	**TV time** was not cross-sectionally associated with **BMI** at age 2.29 yr (*r* = 0.01, p > 0.05), but was unfavourably associated with **BMI** at age 4.25 yr (*r* = 0.03, p < 0.05).	N/A
Williams et al. 2008 [23]; USA<br><br>Children's Activity and Movement in Preschool Study (CHAMPS)	Cross-sectional	N = 198<br><br>Approximate age:<br>3 yr and 4 yr<br>Pre-schoolers	**Total sedentary time** (hr/day; <37.5 counts/15 sec epoch) measured objectively by accelerometer (Actigraph, 7164).	**BMI z-score** (no units) calculated from objectively measured height and weight	**Total sedentary time** was not associated with **BMI z-score** (*r* = -0.09, p > 0.05).	N/A
Chiasson et al. 2016 [24]; USA	Cross-sectional	N = 47,287<br><br>Approximate age:<br>3 yr<br>Toddlers	**Screen time** (hr/day; TV videos, DVDs, using the computer, computer games) assessed by parent-report interview (unpublished format).	**Weight status** (CDC cut-points; categories: not obese, <85th percentile; obese, ≥95th percentile) determined from BMI (calculated from objectively measured height and weight).	**Screen time** was unfavourably associated with **obese weight status** (*unadjusted:* compared to >2 hr/day, ≤2 hr/day: OR = 0.85, 95% CI: 0.77, 0.93, p < 0.001; *adjusted:* OR = 0.87, 95% CI: 0.79, 0.95, p < 0.001).	Race, residence, birthweight, breastfeeding package, exposure to new "Women Infants and Children" food package, healthy food daily.
Dennison et al. 2002 [25]; USA	Cross-sectional	N = 1182<br><br>Age range: 1 to 4 yr<br>Toddlers and Pre-schoolers	**Screen time** (hr/day; TV, video) assessed by parent-report questionnaire (unpublished).	**Weight status** (categories: overweight, > 85th percentile; non-overweight, ≤85th percentile) determined from BMI (calculated from objectively measured height and weight).	**Screen time** was not associated with **weight status** (OR = 0.99, 95% CI: 0.91, 1.08, p = 0.80).	Child age, child sex, parental educational attainment, race/ethnicity, maternal BMI, TV set in bedroom.
Twarog et al. 2015 [26] ; USA	Cross-sectional	N = 1282	**Screen time** (hr/day; TV and videos) assessed by	**Weight status** (CDC cut-points; categories: not	**Screen time** was unfavourably associated	Age, race/ethnicity, gender, poverty status.

National Health and Nutrition Examination Survey (NHANES) (2009 to 2012)		Age range: 2 to 4 yr Toddlers and Pre-schoolers	parent-report using the Physical Activity section of the Family Questionnaire used in NHANES.	obese, <95th percentile; obese, ≥95th percentile) determined from BMI (calculated from objectively measured height and weight).	with **obese weight status** (compared to not obese weight status, odds for screen time >2 hr/day with obese weight status, OR = 1.58, 95% Wald Confidence Interval: 1.03, 2.44, p < 0.05).	
Manios et al. 2009 [27]; Greece<br><br>The Growth, Exercise and Nutrition Epidemiological Study in preSchoolers (GENESIS)	Cross-sectional	N = 2374<br><br>Age Range: 1 to 5 yr Toddlers and Pre-schoolers	**TV time** (hr/day) assessed by parent-report questionnaire (unpublished).	**Weight status** (CDC cut-points; categories: underweight/normal weight, overweight, obese) determined from BMI (calculated from "recorded" height and weight).	Children with **≥2 hr/day TV time** had a higher prevalence of **overweight** (18.6% vs 16.8%) and **obesity** (21.7% vs. 16.1%) than children with **<2 hr/day** (p = 0.003).<br><br>In those **aged 3-5 yr**, but not those **<3 yr** (data not reported), **≥2 hr/day TV time** was unfavourably associated with **obesity** in *unadjusted* (compared to <2 hr/day, *unadjusted*: OR = 1.3, 95% CI: 1.01, 1.70, p = 0.049), but not *adjusted* analyses (*adjusted:* OR = 1.26, 95% CI: 0.89, 1.79, p > 0.05).<br><br>In those **aged 3-5 yr**, each additional **hour of TV per day** was associated with 12% higher probability of having **obese weight status** (*unadjusted:* OR = 1.12, 95% CI: 1.03, 1.26; p = 0.042).	Child's gender, birthweight for gestational age, weight gain in the first 6 months, place of living, mother's educational status, mother's TV viewing time, father's TV viewing time, mother's smoking during pregnancy, parental weight status, *child's physical activity*, child's total energy intake.

Asplund et al. 2015 [28]; USA	Cross-sectional	N = 302<br><br>Age range: 0 to 5 yr Also grouped into those <2 yr and ≥2 yr<br>Infants, Toddlers, Pre-schoolers	**Total screen time** (hr/day; TV, video games, computers, cell phones and other electronic devices) assessed by parent-report questionnaire (unpublished).<br><br>Children were grouped into **categories based on "screen time guideline adherence"**: for those aged <2 yr, "no screen time", or "greater than no screen time"; for those aged "≥2 yr", <2 hr/day, or "≥2 hr/day".	**Weight status** (WHO cut-points, categories: underweight, healthy weight, overweight, obese) determined from BMI percentile (calculated from objectively measured height and weight).	**Total screen time** did not differ by **weight status** category (obese, 1.6 hr/day, SD = 1.1; overweight, 1.8 hr/day, SD = 1.7; healthy weight, 1.6 hr/day, SD = 1.7; p = 0.51).<br><br>There was no association between **"guideline adherence"** and **weight status** in children < 2 yr of age (% in each category with no screen time: obese, 28; overweight, 37; healthy weight, 56; p = 0.06) or ≥2 yr of age (% in each category with <2hr/day screen time: obese, 65; overweight, 59; healthy weight, 54; p = 0.50).<br><br>Children who were **overweight/obese** were not more or less likely to **meet the screen time guidelines** than children who were **normal weight** (healthy weight, reference; ages <2 yr: overweight/obese, OR = 0.35, 95% CI: 0.12, 1.05; ages ≥2 yr, overweight/obese, OR = 1.51, 95% CI: 0.67, 3.42).	Age, parent age, ethnicity, survey language, parent-weighted daily screen time, TV on during dinner, and number of TVs in home.

Nelson et al. 2006 [29]; USA	Cross-sectional	N = 375<br><br>Age range: 2 to 4 yr<br>Toddlers and Pre-schoolers	**Screen time** (min/day; TV and computer) assessed by parent-report questionnaire (modified NHANES 1999-2003 questionnaire).	**Weight status** (CDC cut-points; categories: at risk of overweight/overweight, ≥85th percentile; healthy weight, <85th percentile) determined from BMI (calculated from objectively measured height and weight).	**Screen time** was not associated with **weight status** (compared to <2 hr: OR = 1.54, 95% CI: 0.99, 2.38, p = 0.07) in *unadjusted analysis*, but was unfavourably associated with **at risk of overweight/overweight** status (compared to <2 hr: OR = 1.74, 95% CI = 1.00, 3.04, p = 0.05) in *adjusted analysis*.	Race, ethnicity, asthma, non-juice fruit drink, *physical activity*.
LaRowe et al. 2010 [30]; USA<br><br>Healthy Children Strong Families (HCSF)	Cross-sectional	N = 135<br><br>Age range: 2 to 5 yr<br>Toddlers and Pre-schoolers	**TV time** (hr/day) assessed by parent-report questionnaire (unpublished).<br><br>**Sedentary time** (hr/day; Pfeiffer et al. 2006 or Evenson et al. 2008 cut-points) measured objectively by accelerometer (Actical).	**Weight status** (CDC cut-points; categories: normal weight, <85th percentile; overweight, ≥85th percentile) determined from BMI (unclear if calculated from objectively measured or proxy-reported height and weight).	**TV time** was not different between children with **overweight** and **normal weight status** (mean TV time: overweight, 2.17, SD = 0.18 hr/day; normal weight, 1.83, SD = 0.14 hr/day; p < 0.23).<br><br>**Sedentary time** was not different between children with **overweight** and **normal weight status** (mean sedentary time: overweight, 9.4, SD = 0.61 hr/day; normal weight, 8.5, SD = 0.40 hr/day, p = 0.44).	
Fulton et al. 2009; USA [31]; National Health and Nutrition Examination Survey (NHANES; 1999 to 2006)	Cross-sectional	N = 2861<br>Girls, n = 1423<br>Boys, n = 1438<br><br>Age range: 2 to 5 yr<br>Toddlers to Pre-schoolers	**Screen time** (hr/day; TV, video, computer) assessed by interviewer-administered parent-report questionnaire (unpublished).	**Weight status** (CDC cut-points; categories: normal weight, BMI <85th percentile; overweight/obese, BMI ≥85th percentile) determined from BMI (calculated from	**Screen time** was not associated with **overweight/obese weight status** in *boys* (compared to 0 to 2 hr/day: *unadjusted:* 2.5 to 4 hr/day, OR = 1.2, 95% CI: 0.8, 1.7; ≥4.5 hr/day, OR	Race/ethnicity

				objectively measured height and weight).	= 1.4, 95% CI: 0.8, 2.3; *adjusted:* 2.5 to 4 hr/day, OR = 1.2, 95% CI: 0.8, 1.7; ≥4.5 hr/day, OR = 1.5, 95% CI: 0.9, 2.6) or *girls* (compared to 0 to 2 hr/day: *unadjusted:* 2.5 to 4 hr/day, OR = 1.0, 95% CI: 0.7, 1.4; ≥4.5 hr/day, OR = 1.4, 95% CI: 0.9, 2.3; *adjusted:* 2.5 to 4 hr/day, OR = 1.0, 95% CI: 0.7, 1.4; ≥4.5 hr/day, OR = 1.4, 95% CI: 0.8, 2.3).	
Chen et al. 2011 [32]; USA	Cross-sectional	N = 2036<br><br>Age range: 2 to 5.99 yr<br>Toddlers and Pre-schoolers	**Screen time** (hr/day) assessed by parent-report questionnaire (unpublished).	**Weight status** (CDC cut-points; categories: normal weight, <85th percentile; overweight, ≥85th to 90th percentile; obese, ≥95th percentile) determined from BMI (calculated from objectively measured height and weight).	**Screen time** was not associated with **overweight status** (compared to 3+ hr/day, ≤2 hr/day: OR = 0.91, 95% CI: 0.66, 1.27, p = 0.599) but was unfavourably associated with **obese weight status** (compared to 3+ hr/day, ≤2 hr/day: OR = 0.67, 95% CI: 0.46, 0.98, p = 0.039).	Gender, age, ethnicity, *physical activity*, fruit and vegetables, sweet drinks, fast food, chips.
Olafsdottir et al. 2014 [13]; Estonia, Sweden, Germany, Belgium, Hungary, Italy, Spain and Cyprus<br><br>IDEFICS	Cross-sectional	N = unclear (only the total study sample is reported, but this includes other age groups that are not of interest)<br><br>Age range: 2 to < 6 yr<br>Toddlers and Pre-schoolers	**TV time** (hr/day; TV, video, DVD) and **screen time** (hr/day; TV, video, DVD, computer) assessed by parent-report questionnaire (unpublished).	**BMI** (kg/m^2) calculated from objectively measured height and weight.<br><br>**Waist-to-height ratio** (no units; ratio) calculated from objectively measured height and waist circumference (measured at the midpoint between the iliac crest and the lower costal border or 10th	**TV time** was unfavourably associated with having **overweight/obese weight status** (OR = 1.23, 95% CI: 1.11, 1.35, p < 0.01) and with having a **WHtR >5** (OR = 1.17, 95% CI: 1.07, 1.27, p < 0.01).<br><br>**Screen time** was unfavourably associated	Age, sex, parental education, intervention and study centre.

				rib).<br><br>**Weight status** (IOTF cut-points [14]; categories: normal weight or underweight, overweight or obese) determined from BMI (calculated from objectively measured height and weight).	with having **overweight/obese weight status** (OR = 1.19, 95% CI: 1.10-1.29, p < 0.001) and with having a **WHtR > 5** (OR = 1.13, 95% CI: 1.05, 1.21, p < 0.001).	
Kourlaba et al. 2009 [33]; Greece<br><br>The GENESIS Study	Cross-sectional	N = 2374<br><br>Age range: 1 to 2 yr (Toddlers) and 3-5 yr (Pre-schoolers)	**TV time** (hr/day) assessed by parent-report questionnaire (unpublished).	**Weight status** (CDC cut-points; categories: underweight/normal weight, overweight, obese) determined from BMI (calculated from “recorded” height and weight).	**TV time** was not different between those who were **normal weight** (1-2 yr, 0.73, SD = 0.88 hr/day; 3-5 yr, 1.52, SD = 1.09 hr/day), **overweight** (1-2 yr, 0.97, SD = 1.02; 3-5 yr, 1.48, SD = 1.06), and **obese** (1-2 yr, 0.85, SD = 0.99 hr/day; 3-5 yr, 1.66, SD = 1.16 hr/day) (p > 0.05).	
Burdette and Whitaker 2005 [34]; USA; Fragile Families and Child Wellbeing Study	Cross-sectional	N = 2291<br><br>Mean age: 39 mo (~3.25 yr)<br>Pre-schoolers	**TV time** (min/day) assessed by parent-report interview (unpublished format).	**BMI** (kg/m^2) calculated from objectively measured height and weight.	**TV time** was not correlated with **BMI** (*r* = -0.001, p = 0.952; values from author correspondence).	N/A
Dolinsky et al. 2011 [35]; USA; KAN-DO Study	Cross-sectional	N = 329<br><br>Mean age: 3.5 yr<br>Pre-schoolers	**Total sedentary time** hr/day; <12 counts/15 sec epoch) measured objectively by accelerometer (Actical; Mini Mitter Co, Inc.).	**Weight status** (cut-points from American Academy of Pediatrics 2007 [36]; categories: underweight, <5th percentile; healthy weight, ≥5th to <85th percentile; overweight, ≥85th to <95th percentile; obese, ≥95th percentile) determined from	**Total sedentary time** was not associated with **weight status** (Reference, underweight; Healthy weight: % difference = 0.9, 95% CI: -6.5, 8.9; Overweight: % difference = -2.4, 95% CI: -10.1, 5.9; Obese: % difference = 0.2, 95% CI: -8.1, 9.2; p =	Study site, child’s mean hours per day of accelerometry monitoring.

				objectively measured height and weight.	0.32).	
Minh Do et al. 2015 [37]; Vietnam	Cross-sectional	N = 2,677;<br>Urban, n = 1,364;<br>Rural, n = 1,313<br><br>Mean age:<br>Urban, 4.3 yr<br>Rural, 4.0 yr<br>Pre-schoolers	**Screen time** (hr/day; TV time and time spent playing computer games) assessed by parent-report questionnaire (unpublished).	**Weight status** (WHO cut-points; categories: overweight/obese, other) determined from BMI z-scores (calculated from objectively measured height and weight).	The **odds of overweight/obesity** were not different between those with **<2 or ≥ 2 hr/day screen time** in an **urban** (unadjusted: <2 hr/day, reference; ≥2hr/day, OR = 0.79, 95% CI: 0.56, 1.11, p = 0.16; adjusted: OR = 0.75, 95% CI: 0.50, 1.12) or **rural** (unadjusted: <2 hr/day, reference; ≥ 2 hr/day, OR = 0.38, 95% CI: 0.12, 1.23, p = 0.06; adjusted: OR = 0.74, 95% CI: 0.21, 2.57) sample of children.	Sex, age, amount of food, fatty food, fried food, irregular snack, eating speed, *outdoor activity*, *indoor activity*, mother's education, household size, watching food advertisements on TV by mother, watching food advertisements on TV by father, snack availability.
Hajian-Tilaki and Heidari 2013 [38]; Iran	Cross-sectional	N = 760<br><br>Age range: 2-5 yr<br>Mean age:<br>Boys, 4.23 yr;<br>Girls, 4.24 yr<br>Pre-schoolers	**TV time** (hr/day) and **time spent playing computer games** (hr/day) assessed by parent-report questionnaire (unpublished).	**Weight status** (CDC cut-points; categories: overweight/obese, ≥85th percentile; normal/underweight, <85th percentile) determined from BMI (calculated from objectively measured height and weight).	**TV time** of ≥ 2 hr/day did not elevate odds of **overweight/obesity** compared to <2 hr/day (OR = 1.31, 95% CI: 0.92, 1.82, p = 0.13).<br><br>**Playing computer games** for ≥ 1 hr/day did not elevate the odds of **overweight/obesity** compared to <1 hr/day (OR = 1.46, 95% CI: 0.97, 2.19, p = 0.06).	N/A
Mendoza et al. 2007 [39]; USA<br><br>NHANES; 1999-2002	Cross-sectional	N = 1340<br><br>Mean age: 3.5 yr<br>Pre-schoolers	**TV/video time** (hr/day), **computer time** (hr/day), and **screen time** (hr/day; TV/video and computer time) assessed by parent-report questionnaire	**Weight status** (WHO cut-points; categories: overweight/at risk for overweight, ≥85th percentile; normal weight, ≥5th and <85th percentile;	**TV time** (>2 hr/day vs ≤2 hr/day) was unfavourably associated with being **overweight or at risk for overweight** (PR = 1.34, 95% CI: 1.07, 1.66, p =	Age, gender, race/ethnicity, income.

			(unpublished).	underweight excluded) determined from BMI (calculated from objectively measured height and weight).<br><br>**Sum of skinfolds** (triceps and subscapular; mm) measured using standardized techniques and equipment.	0.01) and with **skinfold thickness** (β = 1.08, 95% CI: 0.19, 1.96, p = 0.02).<br><br>**Computer use** (>0 hr/day vs 0 hr/day) was not associated with **weight status** (PR = 1.16, 95% CI: 0.89, 1.51, p > 0.05) but was unfavourably associated with **skinfold thickness** (β = 0.56, 95% CI: 0.04, 1.07, p = 0.04).<br><br>**Screen time** (>2 hr/day vs ≤2 hr/day) was not associated with **weight status** (PR = 1.21, 95% CI: 0.96, 1.54, p > 0.05) or **skinfold thickness** (β = 0.85, 95% CI: -0.04, 1.75, p = 0.061).	
Sijtsma et al. 2015 [40]; The Netherlands; Groningen Expert Center for Kids with Obesity (GECKO) Drenthe birth cohort	Cross-sectional	N = 759<br><br>Age range: 3 to 4 yr<br>Pre-schoolers	**Screen time** (min/day; TV and computer) assessed by parent-report questionnaire (unpublished).	**BMI** (kg/m^2) calculated from objectively measured height and weight.	**Screen time** was unfavourably associated with **BMI** (*r* = 0.101, p = 0.006).<br><br>In path analysis, **screen time** was directly unfavourably associated with **BMI** (screen time → BMI: B = 0.0027, 95% CI: 0.0004, 0.0050, p = 0.024) and indirectly unfavourably associated with **BMI** via reduced sleep duration (screen time → sleep duration → BMI: B = 0.0004, 95%	Gender; *sleep duration* is a mediator.

					CI: 0.0001, 0.0010, $p < 0.05$). Therefore 1 hr (60 min) of screen time is directly associated with a higher BMI of 0.0027*60=0.16 kg/m^2, and 60*0.1153=7 min less sleep (i.e., screen time → sleep), which is associated with 7*0.0038=0.03 higher BMI (sleep → BMI), for a total effect of 0.16+0.03=0.19 kg/m^2.	
Proctor et al. 2003 [41]; USA; Framingham Children's Study	Cross-sectional	N = 103<br><br>Mean age: 4.0 yr<br>Pre-schoolers	**TV time** (hr/day) assessed by parent-report questionnaire (unpublished).	**BMI** (kg/m^2) calculated from objectively measured height and weight.<br><br>**Triceps skinfold thickness** (mm), and **sum of skinfolds** (mm; triceps, subscapular, suprailiac, abdominal, thigh), measured using Lange calipers and a standard protocol.	**TV time** was not associated with **BMI** (BMI by tertile of TV time: Lowest, 16.3 kg/m^2, SD = 1.1; Middle, 16.1 kg/m^2, SD = 1.2; Highest, 16.2 kg/m^2, SD = 1.1; $p > 0.05$), **triceps skinfold** (triceps skinfold by tertile of TV time: Lowest, 11.5 mm, SD = 2.5; Middle, 10.9 mm, SD = 2.4; Highest, 11.5 mm, SD = 2.1; $p > 0.05$) or **sum of skinfolds** (sum of skinfolds by tertile of TV time: Lowest, 41.6 mm, SD = 10.8; Middle, 41.5 mm, SD = 10.4; Highest, 43.0 mm, 11.0; $p > 0.05$).<br><br>**Tertiles of TV time**: Lowest, 1.1 hr/day, SD = 0.5; Middle, 1.6 hr/day, SD = 0.7; Highest, 2.4 hr/day, SD = 1.6.	N/A

Byun et al. 2013 [42] ; USA<br><br>CHAMPS<br><br>*and*<br><br>Environmental Determinants of Physical Activity in Preschool Children (EDPAPC) study	Cross-sectional	CHAMPS sample:<br>N = 263<br>Mean age, 4.2 yr<br>Pre-schoolers<br><br>EDPAPC sample:<br>N = 155<br>Mean age, 4.0 yr<br>Pre-schoolers	**Total sedentary time** (min/hr; measured throughout the whole day in the CHAMPS sample and the school day in the EDPAPC sample, over a 2-week period) measured objectively by accelerometer (Actigraph, 7164).<br><br>Different accelerometry cut-points were applied to determine whether the association was dependent on the cut-points used:<br>– <37.5 counts/15 sec; Pate et al. 2006 [43]<br>– <200 counts/15 sec; Pate et al. 2006 [43]<br>– <373 counts/15 sec van Cauwenberghe et al. 2011 [44]	**BMI z-score** (no units) calculated from objectively measured height and weight.	In both samples, there was no association between **sedentary time** and **BMI z-score**, regardless of the accelerometer cut-points used.<br><br>*CHAMPS sample:*<br>– <37.5 counts/15 sec: β = -0.019, 95% CI: -0.078, 0.039, p = 0.53<br>– <200 counts/15 sec: β = -0.076, 95% CI: -0.235, 0.083, p = 0.35<br>– <373 counts/15 sec: β = -0.333, 95% CI: -1.342, 0.676, p = 0.52<br><br>*EDPAPC sample:*<br>– <37.5 counts/15 sec: β = -0.041, 95% CI: -0.275, 0.192, p = 0.73<br>– <200 counts/15 sec: β = -0.247, 95% CI: -0.581, 0.086, p = 0.14<br>– <373 counts/15 sec: β = -0.417, 95% CI: -0.991, 0.156, p = 0.15.	Age, gender, race, parent education, preschool, *moderate-to-vigorous intensity physical activity*.
Brown et al. 2010 [45]; Australia; Longitudinal Study of Australian Children (LSAC)	Cross-sectional	N = 4965<br><br>Age range:<br>T1: 4 to 5 yr<br>Pre-schoolers	**TV time** (hr/day; TV, video, DVD, or movie) assessed by parent-report time-use diary (unpublished).	**Weight status** (IOTF cut-points; categories: non-overweight, overweight/obese) determined from BMI (calculated from objectively measured height and weight).	**TV time** was unfavourably associated with **overweight/obese weight status** (for every additional hour of TV, OR = 1.06, 95% CI: 1.01, 1.11; B = 0.056, p < 0.05).	N/A

Taverno Ross et al. 2013 [46]; USA<br><br>Study of Health and Activity in Preschool Environments (SHAPES)	Cross-sectional	N = 339<br><br>Mean age: 4.5 yr<br>Pre-schoolers	**TV time** (categories: **high TV** and **low TV**, based on combined scores for TV time at home and TV exposure at preschool) assessed by parent-report and preschool director-report questionnaires (unpublished).<br><br>At home: High TV, ≥2 hr/day; Low TV, <2 hr/day.<br><br>At preschool: High TV, above the mean questionnaire score; Low TV, below the mean questionnaire score<br><br>Combined: High TV, in the “high” group both at home and preschool; Low TV, all other TV exposure combinations.	**BMI** (kg/m^2 and z-score) calculated from objectively measured height and weight.<br><br>**Waist circumference** (cm) measured objectively (with the child in the standing position; landmarks not specified).	**BMI** was not different between the **low TV** and **high TV** groups (*unadjusted:* low TV vs high TV; absolute: 16.3, SD = 1.7 kg/m^2 vs 16.4, SD = 2.3 kg/m^2, p = 0.59; z-scores: 0.5, SD = 1.0 vs 0.5, SD = 1.2, p = 0.80; *adjusted:* low TV vs high TV; absolute: 16.4, SD = 0.2 kg/m^2 vs 16.2, SD = 0.3 kg/m^2, p = 0.65; z-scores: 0.6, SD = 0.1 vs 0.3 SD = 0.2, p = 0.14).<br><br>**Waist circumference** was not different between the **low TV** and **high TV** groups (*unadjusted:* 52.8, SD = 0.3 cm, vs 52.4, SD = 0.7, p = 0.68; *adjusted:* 53.0, SD = 0.4 cm, vs 52.7, SD = 0.7, p = 0.61).	Gender, race/ethnicity and socioeconomic status ((SES) (parental education)), length of school day.
Boling Turer et al. 2013 [47]; USA<br><br>Kids and Adults Now: Defeat Obesity! (KAN-DO)	Cross-sectional	N = 400<br><br>Mean age: 42 mo (3.5 yr)<br>Pre-schoolers	**Screen time** (hr/day; TV, videos, computer, computer games) assessed by parent-report questionnaire (unpublished).	**Weight status** (CDC cut-points; categories: not overweight; overweight, ≥85th percentile) determined from objectively measured weight and length.	**Screen time** was not associated with **weight status** (compared to ≤2 hr/day: *unadjusted:* OR = 1.0, 95% CI: 0.6, 1.6; *adjusted:* OR = 1.2, 95% CI: 0.7, 1.9).	Age (in months), gender, race/ethnicity, and maternal weight status.
Collings et al. 2013 [48]; England<br><br>Southampton	Cross-sectional	N = 398<br><br>Median age: 4.1 yr<br>Pre-schoolers	**Total sedentary time** min/day; <30 counts/60 sec epoch) measured objectively by	**Percentage body fat** (**%BF;** (%) measured objectively using DXA.	In *unadjusted analyses*, **sedentary time** was unfavourably associated with **%BF** (*r* = 0.08, p <	Age, sex, birth weight, maternal education, maternal BMI, smoking during pregnancy, *sleep*

Women's Survey			accelerometer (Actiheart).	**Fat mass index** (FMI; kg/m^2), **lean mass index** (LMI; kg/m$^{2.5}$) and **trunk fat mass index** (TFMI; kg/m^2) calculated from objectively measured height and fat mass, lean mass, and trunk fat mass respectively.	0.001), **FMI** (*r* = 0.058, p < 0.01), **LMI** (*r* = -0.084, p < 0.001) and **TFMI** (*r* = 0.062, p < 0.01).<br><br>In *adjusted analyses*, **sedentary time** was not associated with **% BF** (β = 0.013, 95% CI: -0.54, 0.57, p = 0.96), **FMI**, (β = -0.7, 95% CI: -3.30, 1.97, p = 0.60), **LMI** (β = -0.058, 95% CI: -0.16, 0.040, p = 0.25), or **TFMI** (β = -0.26, 95% CI: -3.67, 3.26, p = 0.88).<br>Note: β's are interpreted as the % change in outcome variable for a 120 min/day change in total sedentary time.	*duration, moderate-to-vigorous intensity physical activity.*
Espana-Romero et al. 2013 [49]; USA; Study of Health and Activity in Preschool Environments (SHAPES)	Cross-sectional	N = 357<br>Boys, n = 183<br>Girls, n = 174<br><br>Mean age:<br>Boys, 4.5 yr<br>Girls, 4.6 yr<br>Pre-schoolers	**Total sedentary time** (min/hr; ≤200 counts/15 sec epoch; Pate et al. 2006 [43]) measured objectively by accelerometer (ActiGraph models GT1M and GT3X).	**BMI z-score percentile** (no units) calculated from objectively measured height and weight.<br><br>**Waist circumference** (cm and percentile; measured to the nearest 0.1 cm midway between the inferior edge of the lowest rib and the superior border of the iliac crest).	**Total sedentary time** was not associated with **BMI z-score percentile** in *boys* (β = -0.050, SE = 0.028, p = 0.07) or *girls* (β = 0.014, SE = 0.023, p = 0.51).<br><br>**Total sedentary time** was not associated with **waist circumference** in *boys* (β = -0.152, SE = 0.113, p = 0.18) or *girls* (β = 0.154, SE = 0.117, p = 0.19).<br><br>Using quantile regression, **total sedentary behaviour** was not associated with **BMI z-**	**Total sedentary time** and **BMI z-score percentile** or **waist circumference:** Race/ethnicity, parental education, preschool.<br><br>**Total sedentary time** and **waist circumference percentile:** Race/ethnicity, parental education.

					**score percentile** in *boys* (10th: β = -0.017, SE = 0.045; 25th: β = -0.033, SE = 0.034; 50th: β = -0.054, SE = 0.034; 75th: β = -0.072, SE = 0.039; 90th: β = -0.092, SE = 0.074; all p > 0.05) or *girls* (10th: β = 0.007, SE = 0.036; 25th: β = -0.010, SE = 0.035; 50th: β = -0.003, SE = 0.024; 75th: β = -0.024, SE = 0.03; 90th: β = 0.038, SE = 0.039; all p > 0.05).<br><br>Using quantile regression, **total sedentary behaviour** was not associated with the 0th, 25th, 50th, 75th or 90th **waist circumference percentiles** in *boys* (10th: β = -0.022, SE = 0.156; 25th: β = -0.195, SE = 0.108; 50th: β = -0.039, SE = 0.143; 75th: β = -0.094, SE = 0.158; 90th: β = -0.247, SE = 0.314; all p > 0.05), but in *girls* there was an unfavourable association between **total sedentary time** and the 90th **waist circumference percentile** (10th: β = -0.060, SE = 0.146; 25th: β = -0.079, SE = 0.147; 50th: β = 0.071, SE = 0.102; 75th: β = 0.005, SE = 0.128; 90th: β = 0.441, SE	

					= 0.191; 90th percentile p < 0.05, all others p > 0.05).	
Lioret et al. 2007 [50]; France; French INCA food consumption survey	Cross-sectional	N = 234<br><br>Age range: 3 to 5 yr<br>Pre-schoolers	**Screen time** (hr/day and categories: "low", 20th percentile; "intermediate" 20-80th percentile; "high", 80th percentile; TV and video games) assessed by self-report questionnaire (unpublished).	**Weight status** (IOTF cut-points; categories: normal weight, overweight/obese) determined from BMI (calculated from self-reported height and weight).	**Screen time** was not associated with **overweight/obese weight status** (compared to "low" screen time: "intermediate", OR = 0.9, 95% CI: 0.3, 2.5; "high", OR = 2.1, 95% CI: 0.7, 6.6).	SES, sex, age, *leisure time physical activity*, total energy intake.
Koubaa et al. 2012 [51]; Tunesia	Cross-sectional	N = 121<br><br>Median age: 4.8 yr<br>Pre-schoolers	**Screen time** (hr/day; TV and playing video games) assessed by parent-report questionnaire (unpublished).	**Weight status** (Rolland Cachera reference curves; categories: healthy weight, ≤97th percentile; overweight, >97th percentile) determined from BMI calculated from objectively measured height and weight.	The prevalence of >1hr/day **screen time** was not different between the **healthy weight** (69%) and **overweight** (84%) groups (p = 0.09).	N/A
Bonvin et al. 2012 [52]; Switzerland<br><br>Youp'la bouge study	Cross-sectional	N = 251<br><br>Mean age: 3.4 yr<br>Pre-schoolers	**Total sedentary time** epochs/hr <37.5 counts; <37.5 counts/15 sec epoch) measured objectively by accelerometer (ActiGraph GT1M).	**Weight status** (IOTF cut-points; categories: healthy weight, excess weight) determined from BMI (calculated from objectively measured height and weight).	**Sedentary time** was not different between children classified as having **healthy weight** (130, SD = 22 epochs/hr <37.5 counts) or **excess weight** (132, SD = 24 epochs/hr <37.5 counts).<br><br>**Sedentary time** was not associated with **weight status** (β = 1.6, 95% CI: -5.4, 8.5, p ≥ 0.6).	Age, sex, child care center.
van Stralen et al. 2012 [53]; Germany and Greece	Cross-sectional	N = 3664<br>Germany, n = 2956;<br>Greece, n = 708	**TV time** (hr/day) and **screen time** (hr/day) assessed by parent-report questionnaire	**BMI** (kg/m^2) calculated from objectively measured height and weight.	*Germany:*<br>**Screen time** was unfavourably associated with **BMI** (B = 0.14, 95%	*Germany:*<br>Sex, age.<br><br>*Greece:*

TigerKids<br><br>*and*<br><br>GENESIS		Mean age:<br>Germany, 4.5 yr<br>Greece, 4.4 yr<br>Pre-schoolers	(unpublished).	**Waist circumference** (cm) measured objectively (landmarks not specified).	CI: 0.08, 0.12; β = 0.10, p < 0.05).<br><br>*Greece:*<br>**TV time** was not associated with **BMI** (B = 0.003, 95% CI: -0.003, 0.009, β = 0.039, R^2 = 0.006, p > 0.05) or **waist circumference** (B = 0.001, 95% CI: -0.003, 0.006, β = 0.026, R^2 = 0.015, p > 0.05).	Sex, age, maternal education.
Byun et al. 2011 [54]; USA<br><br>CHAMPS	Cross-sectional	N = 331<br><br>Mean age: 4.3 yr<br>Pre-schoolers	**Total sedentary time** (min/hr; <37.5 counts/15 sec epoch) measured objectively by accelerometer (ActiGraph 7164).	**BMI z-score** (no units; calculated as the deviation of each participant's value from the mean values reported in CDC growth charts) calculated from objectively measured height and weight.	*Unadjusted analysis:*<br>**Sedentary time** was favourably associated with **BMI z-scores** (*r* = -0.16, p ≤0.05).<br><br>*Adjusted analysis:*<br>**Sedentary time** was not associated with **BMI z-scores** (β = -0.30, SE = 0.1, p ≤0.10).	Gender, adult job, birth weight, , family support for physical activity, , perceived enough physical activity, *perceived level of physical activity*, athletic coordination, weekday TV/video game, physical activity equipment.
Tremblay and Rinaldi 2010 [55]; Canada<br><br>QLSCD	Cross-sectional	N = 1192<br>Girls, n = 581<br>Boys, n = 611<br><br>Approximate age: 4 yr<br>Pre-schoolers	**TV time** (hr/day) on weekdays and weekend days assessed by parent-report questionnaire (unpublished).	**BMI** (kg/m^2) calculated from objectively measured height and weight.<br><br>**Skinfold ratio** (no units; ratio of triceps skinfold thickness to subscapular skinfold thickness – i.e., limb to trunk) calculated from objectively measured skinfold thicknesses.	**TV time on weekdays** was not associated with **BMI** (*boys*: *r* = -0.005, p > 0.05; *girls: r* = 0.059, p > 0.05).<br><br>**TV time on weekdays** was not associated with **skinfold ratio** in *boys* (*r* = -0.072, p > 0.05), but was favourably associated with **skinfold ratio** in *girls* (*r* = -0.09, p < 0.05).<br><br>**TV time on weekend**	

					**days** was not associated with **BMI** (*boys: r* = -0.046, p > 0.05; *girls: r* = 0.001, p > 0.05) or **skinfold ratio** (*boys: r* = 0.013, p > 0.05; *girls: r* = -0.068, p > 0.05).	
Sasaki et al. 2010 [56]; Japan	Cross-sectional	N = 449<br><br>Mean age: 4.5 yr<br>Pre-schoolers	**Screen time** (hr/day; TV and computer games) assessed by parent-report questionnaire (unpublished).	**Weight status** (categories: normal, BMI <25 kg/m^2; obese, BMI ≥25 kg/m^2) determined from BMI (calculated from parent-report height and weight).	The prevalence of **obesity** was not associated with **screen time** (prevalence of obesity: <2hr/day screen time, 8.2%; ≥2 hr/day screen time, 8.3%; p = 0.56).<br><br>**Screen time** was not associated with **obese weight status** (*unadjusted:* compared to <2hr/day screen time, ≥2 hr/day: OR = 1.11, 95% CI: 0.50, 2.49, p > 0.05; *adjusted:* compared to <2hr/day screen time, ≥2 hr/day: OR = 1.08, 95% CI: 0.58, 2.44, p > 0.05).	*Sleep duration*, breastfeeding status, maternal obesity, maternal education, maternal smoking.
Anderson and Whitaker. 2010 [57]; USA<br><br>ECLS-B	Cross-sectional	N ≈ 8550<br><br>Mean age: 52.3 mo (~4.4 yr)<br>Pre-schoolers	**Screen time** (hr/weekday; TV, videos, DVDs) on weekdays assessed by parent-report questionnaire (unpublished).	**Weight status** (categories: normal, <95th percentile; obese, ≥95th percentile) determined from BMI (calculated from objectively measured height and weight).	**Screen time** was unfavourably associated with **obese weight status** in *unadjusted* analysis (compared to >2 hr/weekday, ≤2 hr/weekday: OR = 0.77, 95% CI: 0.64, 0.91), but was not associated after adjustments (compared to >2 hr/weekday, ≤2 hr/weekday: OR = 0.85,	*≥10.5 hr of sleep per weekday night*, eating dinner as a family >5 evenings/week, child age, gender, racial/ethnic group, household income-to-poverty ratio, single-parent household, maternal education, maternal BMI, maternal age.

					95% CI: 0.71, 1.03).<br><br>The **prevalence of obesity** was greater in those with >2 hr/day **screen time** (20.0%) than those with ≤2 hr/day **screen time** (16.1%) on weekdays ($p = 0.002$).	
Dubois et al. 2008 [58]; Canada<br><br>Longitudinal Study of Child Development in Quebec (LSCDQ; 2002)	Cross-sectional	N = 1540<br><br>Mean age: 49 mo (~4.1 yr)<br>Pre-schoolers	**TV time** (hr/day) assessed by parent-report interview (unpublished format).	**BMI** (kg/m^2) calculated from objectively measured height and weight.	**BMI** was not different between children with **<3 hr/day TV time** (15.7 kg/m^2) and **≥3 hr/day TV time** (15.7 kg/m^2) ($p > 0.05$).	N/A
Jouret et al. 2007 [59]; France	Cross-sectional	N = 593;<br>Boys, n = 298;<br>Girls, n = 295<br><br>Mean age: 4.6 yr<br>Pre-schoolers	**TV time** (hr/day) assessed by parent-report questionnaire (unpublished).	**Weight status** (French reference standards; ≥90th percentile or z-score ≥1.28 considered overweight/obese; categories: overweight/obese, non-overweight) determined from BMI (calculated from objectively measured height and weight).	In *unadjusted analysis*, **TV time** was unfavourably associated with **overweight status** in *boys* (compared to ≤1 hr/day, >1 hr/day: OR = 3.80, 95% CI: 1.54, 9.38, $p = 0.0042$) but not *girls* (compared to ≤1 hr/day, >1 hr/day: OR = 2.02, 95% CI: 0.89, 4.57, $p = 0.0911$).<br><br>In *adjusted analysis*, **TV time** was unfavourably associated with **overweight status** in *boys* (compared to ≤1 hr/day, >1 hr/day: OR = 3.76, 95% CI: 1.52, 9.31, $p = 0.004$) and *girls*	Participation in organized *physical activity*.

					(compared to ≤1 hr/day, >1 hr/day: OR = 2.43, 95% CI: 1.04, 5.66, p = 0.0401).	
Jiang et al. 2006 [60]; China	Cross-sectional	N = 930<br><br>Mean age: 4.6 yr<br>Pre-schoolers	**TV time** (hr/day) and **time playing computer games** (hr/day) assessed by parent-report questionnaire (unpublished).	**Weight status** (IOTF cut-points; categories: categories: overweight/obese, non-overweight) determined from BMI (calculated from objectively measured height and weight).	**TV time** was unfavourably associated with **overweight status** (*unadjusted:* no values reported; *adjusted:* compared to <2 hr/day, ≥2 hr/day, OR = 1.56, 95% CI: 1.17, 2.09, p = 0.012).<br><br>**Time playing computer games** was not associated with **weight status** (no values reported)	Sex, age, family income, kindergarten class.
Jago et al. 2005 [61]; USA<br><br>Studies of Child Activity and Nutrition (SCAN) Program (Texas site)	Cross-sectional	N = 149<br><br>Mean age: 4.4 yr<br>Pre-schoolers	**TV time** (min/hr) assessed by direct observation (each child was observed for ~6-12hr/day for a mean of 2.l5 days; observers noted whether the child was paying attention to the TV during each minute).<br><br>**Total sedentary time** (min/hr) assessed by direct observation using the Children's Activity Rating Scale (CARS).	**BMI** (kg/m^2) calculated from objectively measured height and weight.	**TV time** was not associated with **BMI** (r = -0.027, p > 0.05).<br><br>**Total sedentary time** was not associated with **BMI** (r = 0.121, p > 0.05).	N/A
Levin et al. 2004 [62]; USA	Cross-sectional	N = 148<br><br>Approximate age: 4 yr<br>Pre-schoolers	**TV time** (hr/week) assessed by parent-report questionnaire (unpublished).	**BMI** (kg/m^2) calculated from objectively measured height and weight.<br><br>**Weight status**	**TV time** was not significantly different across quartiles of **BMI** (25th percentile: 2.5 hr/day; 25-75th percentile:	N/A

				(categories: obese, BMI ≥95th percentile; non-obese) determined from BMI.	3.3 hr/day; 75th percentile: 3.6 hr/day; p-value not reported).<br><br>A greater proportion of children who had **non-obese weight status** (43.9%) had ≤2 hr/day TV time than those who had **obese weight status** (19.4%) (p = 0.01).<br><br>**TV time** was unfavourably associated with **obese weight status** (compared to <1 hr/day: >5 hr/day, OR = 5.0, 95% CI: 0.5, 49.3).	
DuRant et al. 1994 [63]; USA<br><br>Family Health Project (Texas site of the Studies of Child Activity and Nutrition program)	Cross-sectional	N = 110<br><br>Mean age: 4.23 yr<br>Pre-schoolers	**TV time** (%min/day) assessed by direct observation using the CARS. Children were observed up to 4 times over 3 years and observations were averaged.	**BMI** (kg/m^2) calculated from objectively measured height and weight.<br><br>**Sum of skinfolds** (mm; biceps, triceps, substcapular, suprailiac, abdomen, thigh, calf).<br><br>**Waist-to-hip ratio** (no units) calculated from objectively measured circumferences.	**TV time** was not associated with **BMI** (*r* = 0.07, p = 0.450)**, sum of skinfolds** (*r* = 0.13, p = 0.168)**,** or **waist-to-hip ratio** (*r* = 0.06, p = 0.560).	N/A
Cardon et al. 2016 [64]; Belgium, Bulgaria, Germany, Greece, Poland, Spain<br><br>ToyBox-study (baseline data;	Cross-sectional	N = 3301<br><br>Boys, n = 1716<br>Girls, n = 1585<br><br>Mean age: 4.7 yr<br>Pre-schoolers	**TV time** (min/day), **computer time** (min/day), and **screen time** (min/day; TV and computer time) assessed by parent-report questionnaire (Primary Caregivers' Questionnaire (PCQ); www.toybox-	**Weight status** (International Obesity Task Force (IOTF) cut-points; categories: normal weight, overweight/obese) determined from BMI (calculated from objectively measured	**TV time on weekdays** was not associated with **weight status** in *boys* (β=0.024, 95% CI: -0.001, 0.003, p = 0.52) or *girls* (β = -0.032, 95% CI: -0.003, 0.001, p = 0.421).	Country, age, educational level of the mother.

2012)			study.eu).<br><br>**Sedentary quiet play** (min/day; e.g., looking into books, playing with blocks, playing with dolls, drawing, construction) on weekdays and weekend days assessed by parent-report questionnaire (PCQ; www.toybox-study.eu).	height and weight).	**TV time on weekend days** was not associated with **weight status** in *boys* (β=0.038, 95% CI: 0.000, 0.002. p = 0.31) but was unfavourably associated with **weight status** in *girls* (β = 0.145, 95% CI: 0.001, 0.004; p < 0.001).<br><br>**Computer time on weekdays** or **weekend days** was not associated with **weight status** in *boys* (weekdays: β=0.025, 95% CI: -0.004, 0.004, p = 0.53; weekend days: β=0.018, 95% CI: -0.001, 0.003, p = 0.65) or *girls* (weekdays: β=0.032, 95% CI: -0.004, 0.008, p = 0.53; weekend days: β=-0.072, 95% CI: -0.006, 0.001, p = 0.65).<br><br>**Screen time** was not associated with **overweight/obese weight status** in *boys* (compared with <1 hr/day, ≥1 hr/day: weekdays: OR = 1.099, 95% CI: 0.754, 1.601, p = 0.63; weekend days: OR = 1.054, 95% CI: 0.636, 1.748, p = 0.84) or *girls* (compared with <1 hr/day, ≥1 hr/day: weekdays: OR = 1.152, 95% CI: 0.806, 1.647, p = 0.44; weekend	

					days: OR = 1.024, 95% CI: 0.639, 1.640, p = 0.92).<br><br>**Sedentary quiet play** was not associated with **overweight/obese weight status** in *boys* (weekday: β=-0.001, 95% CI: -0.001, 0.001, p = 0.99; weekend: β=-0.040, 95% CI: -0.002, 0.000, p = 0.26) or *girls* (weekday: β=-0.007, 95% CI: -0.002, 0.001, p = 0.99; weekend: β=0.014, 95% CI: -0.001, 0.001, p = 0.26).<br><br>Compared with *boys* who had **<90 min sedentary quiet play per weekday** or **weekend day**, boys with **≥90 min/day** were not more likely to have **overweight/obese weight status** (weekday: OR = 1.073, 95% CI: 0.727, 1.584, p = 0.72; weekend day: OR = 0.993, 95% CI: 0.638, 1.547, p = 0.98).<br><br>*Girls* who engaged in **sedentary quiet play** for **≥90 min/day on weekend days** (but not **weekdays**) were more likely to have **overweight/obese weight status** than those who engaged in <90 min/day	

					(weekday: OR = 0.790, 95% CI: 0.557, 1.122, p = 0.19; weekend day: OR = 1.715, 95% CI: 1.047, 2.810, p = 0.03).	
Harrison et al. 2012 [65]; USA	Cross-sectional	N = 354<br><br>Mean age: 37.37 mo (~3.1 yr)<br>Pre-schoolers	**TV time** (hr/day), **time watching videos/DVDs** (hr/day), **time playing video games** (hr/day), **time using the internet** (hr/day), and **time using books** (hr/day) assessed by parent-report questionnaire (unpublished).	**BMI percentile** (percentile) calculated from objectively measured height and weight.	**TV time** was not associated with **BMI percentile** (r = 0.09, p > 0.05; β = -0.01, p > 0.05).<br><br>**Time watching videos/DVDs** was not associated with **BMI percentile** (r = 0.11, p > 0.05; β = 0.03, p > 0.05).<br><br>**Time playing video games** was unfavourably associated with **BMI percentile** (r = 0.12, p < 0.05; β = 0.10, p < 0.05).<br><br>**Time using the internet** was not associated with **BMI percentile** (r = -0.03, p > 0.05; β = -0.01, p > 0.05).<br><br>**Time using books** was not associated with **BMI percentile** (r = 0.07, p > 0.05; β = 0.08, p > 0.05).	Child race/ethnicity, parent BMI, parent education, household income, child gender, TV time, time watching videos/DVDs, time playing video games, time using the internet, and time using books (where applicable).
Vandebosch and van Cleemput 2007 [66]; Belgium	Cross-sectional	N = 608<br><br>Mean age: 56.3 mo (~4.7 yr)<br>Pre-schoolers	**TV time** (min/week) assessed by parent-report questionnaire (unpublished).	**BMI z-score** (no units) calculated from parent-reported height and weight.<br><br>**Weight status** (Flemish growth charts [67];	In the *full sample*, in *unadjusted analysis*, **TV time** was not associated with **BMI z-score** (r = 0.063, p = 0.174).<br><br>In *adjusted analyses*, **TV**	Age father, age mother, educational level father, educational level mother, percentage employment of the family, BMI father, BMI mother, parent's amount of TV viewing,

				categories: underweight/normal weight; overweight, *boys*, BMI-SD ≥ 1.214, *girls*, BMI-SD ≥ 1.012; obese, *boys*, BMI-SD > 2.13, *girls*, BMI-SD >1.90) determined from parent-reported height and weight.	**time** was favourably associated with **BMI z-score** in *boys* (β = -0.434, p < 0.01) but not *girls* (β = -0.030, p > 0.05).<br><br>Children with **obese weight status** had greater **TV time** (918.9, SD = 390.5 min/week) than those with **underweight/normal weight status** (608.1, SD = 391.2 min/week) (p = 0.001), but those with **overweight weight status** did not (683.5, SD = 388.8 min/week).	media as a cause of childhood obesity, parental mediation of physical activity, parental mediation of food intake, restrictive parental TV mediation, watching TV because of parent time limitations, watching TV because of parent tiredness, age child, TV dependency, child eating meal while watching TV, child snacking while watching TV, TV in child's bedroom.

ALSPAC, Avon Longitudinal Study of Parents and Children; B, unstandardized beta; β, standardized beta; BMI, body mass index; CARS, Children's Activity Rating Scale; CD, compact disk; CDC, Centers for Disease Control and Prevention; CHAMPS, Children's Activity and Movement in Preschool Study; CI, confidence interval; DXA, Duel-energy X-ray absorptiometry; Early STOPP, Early Stockholm Obesity Prevention Project; ECLS-B, Early Childhood Longitudinal Study-Birth Cohort; EDPAPC, Environmental Determinants of Physical Activity in Preschool Children; FMI, fat mass index; GECKO, Groningen Expert Center for Kids with Obesity; GENESIS, Growth, Exercise and Nutrition Epidemiological Study in preSchoolers; HCSF, Healthy Children Strong Families; IDEFICS, Identification and prevention of dietary and lifestyle-induced health effects in children and infants; IOTF, International Obesity Task Force; KAN-DO, Kids and Adults Now: Defeat Obesity!; LMI, lean mass index; LSAC, Longitudinal Study of Australian Children; LSCDQ, Longitudinal Study of Child Development in Quebec; MET, metabolic equivalent of task; NHANES, National Health and Nutrition Examination Survey; NLSY, National Longitudinal Survey for Children and Youth study; NW, normal weight; OB, obese; OR, odds ratio; OW, overweight; PCQ, Primary Caregivers' Questionnaire; PR, prevalence ratio; QLSCD, Quebec Longitudinal Study of Child Development; RRR, relative risk ratio; SCAN; Studies of Child Activity and Nutrition; SE, standard error; SES, socioeconomic status; SD, standard deviation; SHAPES, Study of Health and Activity in Preschool Environments; T1-T4, Time 1 to Time 4; TFMI, trunk fat mass index; TV, television; WHO, World Health Organization; WHtR, Waist-to-height ratio; WIC, Supplemental Nutrition Program for Women, Infants, and Children; %BF, percentage body fat.

Table S2. Summary of findings for motor development outcomes

Reference; Country; Larger Study/Cohort Name	Study Design	Sample	Exposure	Outcome	Main Findings	Covariates included in model (if applicable)
Study Design: Longitudinal						
Hesketh et al. 2015 [68]; Australia; Melbourne Infant Feeding, Activity and Nutrition Trial Program (INFANT)	Longitudinal [~16 mo (1.3 yr) follow-up]	N = 542<br><br>T1, 3.9 mo (0.3 yr) Infants<br><br>T2, 9.1 mo (0.76 yr) Infants<br><br>T3, 19.9 mo (1.7 yr) Toddlers	**TV time** (hr/day), **time spent in a stroller/pram** (hr/day), **time spent in a car seat/capsule** (hr/day), **time spent in a high chair or other chair** (hr/day), **time spent in a playpen** (hr/day), **time spent in a baby carrier/sling** (hr/day) assessed by parent-report questionnaire (unpublished).	**Age at first sitting** (months), **age at first crawling** (months), and **age at first walking** (months), assessed by parent-report questionnaire (unpublished).	**TV time** at age ~4 mo was not associated with **age at first sitting** (β = -0.02, p = 0.629), **age at first crawling** (β = 0.02, p = 0.645), or **age at first walking** (β = -0.02, p = 0.661).<br><br>**Time spent in a stroller/pram** at age ~4 mo was not associated with **age at first sitting** (β = 0.02, p = 0.670), **age at first crawling** (β = 0.03, p = 0.631), or **age at first walking** (β = 0.00, p = 0.941).<br><br>**Time spent in a car seat/capsule** at age ~4 mo was not associated with **age at first sitting** (β = -0.01, p = 0.788), **age at first crawling** (β = -0.07, p = 0.185), or **age at first walking** (β = -0.06, p = 0.096).<br><br>**Time spent in a high chair or other chair** at age ~4 mo was not	Exact age, intervention arm.

					associated with **age at first sitting** (β = -0.02, p = 0.775), **age at first crawling** (β = 0.01, p = 0.753), or **age at first walking** (β = -0.05, p = 0.373).<br><br>**Time spent in a playpen** at age ~4 mo was not associated with **age at first sitting** (β = -0.06, p = 0.203), **age at first crawling** (β = -0.05, p = 0.187), or **age at first walking** (β = -0.06, p = 0.106).<br><br>**Time spent in a baby carrier/sling** at age ~4 mo was not associated with **age at first sitting** (β = -0.07, p = 0.179), **age at first crawling** (β = -0.07, p = 0.194), or **age at first walking** (β = -0.06, p = 0.243).<br><br>**TV time** at age ~9 mo was not associated with **age at first sitting** (β = 0.00, p = 0.957), **age at first crawling** (β = 0.03, p = 0.443), or **age at first walking** (β = -0.02, p = 0.692).<br><br>**Time spent in a stroller/pram** at age ~9	

					mo was not associated with **age at first sitting** (β = -0.09, p = 0.079), **age at first crawling** (β = -0.07, p = 0.243), or **age at first walking** (β = 0.00, p = 0.995).<br><br>**Time spent in a car seat/capsule** at age ~9 mo was favourably associated with **age at first sitting** (β = -0.11, p = 0.017), and **age at first crawling** (β = -0.11, p = 0.012), but was not associated with **age at first walking** (β = 0.04, p = 0.472).<br><br>**Time spent in a high chair or other chair** at age ~9 mo was not associated with **age at first sitting** (β = 0.02, p = 0.751), **age at first crawling** (β = -0.04, p = 0.322), or **age at first walking** (β = -0.00, p = 0.963).<br><br>**Time spent in a playpen** at age ~9 mo was not associated with **age at first sitting** (β = 0.04, p = 0.730), **age at first crawling** (β = -0.02, p = 0.783), or **age at first walking** (β = 0.04, p = 0.540).	

					**Time spent in a baby carrier/sling** at age ~9 mo was not associated with **age at first sitting** (β = -0.02, p = 0.615), **age at first crawling** (β = -0.06, p = 0.226), or **age at first walking** (β = -0.02, p = 0.672).<br><br>**TV time** at age ~20 mo was not associated with **age at first sitting** (β = -0.01, p = 0.857), **age at first crawling** (β = 0.03, p = 0.538), or **age at first walking** (β = 0.04, p = 0.352).<br><br>**Time spent in a stroller/pram** at age ~20 mo was not associated with **age at first sitting** (β = 0.10, p = 0.070), **age at first crawling** (β = 0.04, p = 0.433), or **age at first walking** (β = 0.05, p = 0.281).<br><br>**Time spent in a car seat/capsule** at age ~20 mo was not associated with **age at first sitting** (β = 0.01, p = 0.815), **age at first crawling** (β = -0.02, p = 0.632), or **age at first walking** (β = 0.06, p = 0.212).	

					**Time spent in a high chair or other chair** at age ~20 mo was not associated with **age at first sitting** (β = 0.05, p = 0.435), **age at first crawling** (β = 0.03, p = 0.596), or **age at first walking** (β = 0.04, p = 0.379).<br><br>**Time spent in a playpen** at age ~20 mo was not associated with **age at first sitting** (β = 0.10, p = 0.225), **age at first crawling** (β = -0.01, p = 0.848), or **age at first walking** (β = 0.09, p = 0.164).	
Schmidt et al. 2009 [6]; USA; Project Viva	Longitudinal (2.5 yr follow-up)	N = 872<br><br>T1, Age Range: 6 mo to 2 yr Toddlers to Pre-schoolers<br><br>T2, ~Age: 3 yr Pre-schoolers	**TV time** (hr/day) assessed by parent-report questionnaire (unpublished) at ages 6 mo, 1 yr and 2 yr, and as the weighted average of TV exposure from birth and 2 yr [adapted from questionnaire used in the National Longitudinal Survey of Children and Youth study (NLSY)].	**Visual-motor abilities** (standardized to a mean and SD of 15) assessed by trained research assistants using the Wide-Range Assessment of Visual Motor Ability (WRAVMA) test.	**TV time** from birth to 2 yr was not related to **visual-motor abilities** at age 3 yr (mean WRAVMA score by TV time: 0 to 0.5 hr/day, mean = 103.4, SD = 11.5; 0.5 to < 1 hr/day, mean = 103.1, SD = 11.1; 1 to <2 hr/day, mean = 102.1, SD = 10.8; ≥2 hr/day, mean = 102.2, SD = 11.9; p = 0.57).<br><br>**TV time** from birth to 2 yr was not associated with **visual-motor abilities** at age 3 yr (B = -0.24, 95%	**TV time from age birth to 2 yr:** Age, gender, maternal age, education, marital status, parity, Peabody Picture Vocabulary Test III (PPVT-III) score, household income, child birth weight for gestational age z score, breastfeeding duration, race/ethnicity, English language, *average daily sleep duration from 6 mo to 2 yr*.<br><br>**TV time at ages 6 mo, 1**

					CI: -1.15, 0.66, p > 0.05).<br><br>**TV time** at 6 mo, 1 yr and 2 yr of age was not associated with **visual-motor abilities** at age 3 yr (6 mo: B = 0.01, 95% CI: -0.65, 0.66; 1 yr: B = -0.02, 95% CI: -0.55, 0.52; 2yr: B = -0.29, 95% CI: -1.06, 0.48).	**yr and 2 yr:** Maternal age, education, marital status, parity, PPVT-III score, household income, child birth weight for gestational age z score, breastfeeding duration, race/ethnicity, English language, *average daily sleep duration*.
Pagani et al. 2013 [69]; Canada; QLSCD (Quebec Longitudinal Study of Child Development)	Longitudinal (3 yr follow-up)	N = 1999<br><br>Approximate Age: T1, 29 mo (~2.4 yr) Toddlers<br><br>T2, 65 mo (~5.4 yr) School-Age	**TV time** (hr/day) at age 29 mo assessed by parent-report questionnaire (unpublished).	**Locomotion skills** (no units; higher scores are better) and **object control** (no units; higher scores are better) assessed by the "test of gross motor development".	**TV time** at age ~29 mo was unfavourably associated with **locomotion skills** (B = -0.024, 95% CI: -0.034, -0.014, p < 0.000), but was not associated with **object control** (values not reported; p > 0.05), at age ~65 mo.	Maternal education, early stimulation of literacy, early childhood temperament, family functioning.
Study Design:<br>Cross-sectional						
De Kegel et al. 2012 [70]; Belgium	Cross-sectional	N = 210<br><br>Age range: 4 to 18 mo (~0.33 to 1.5 yr) Infants to Toddlers	**Amount of play time in the supine position** (categories: often to frequently, >30 min/day; sometimes to never, <30 min/day) assessed by parent-report questionnaire (unpublished).	**Gross motor performance** (no units; age-corrected score; greater scores indicate better gross motor performance) assessed using the Alberta Infant Motor Scale (AIMS) tool.	Infants who **played in the supine position often or frequently** *before the age of 6 mo* did not have different **gross motor performance** compared to those who **played in the supine position sometimes to never** (motor performance, means ± SD: often or frequently, -0.38 ± 5.63; sometimes to never, 0.94 ± 8.11; p = 0.355; Cohen's	N/A

					d = -0.19).<br><br>Infants/toddlers who **played in the supine position often or frequently** *after the age of 6 mo* had significantly lower **gross motor performance** compared to those who **played in the supine position sometimes to never** (motor performance, means ± SD: often or frequently, -2.28 ± 6.10; sometimes to never, 1.84 ± 7.48; p = 0.001; Cohen's *d* = -0.60).	
Lin et al. 2015 [71]; Taiwan	Cross-sectional	N = 150<br>TV exposure, n = 75; Control, n = 75<br><br>Mean age: 24.8 mo (~2.1 yr)<br>Toddlers	**TV time** (min/day) assessed by parent-report interview.<br><br>Children were divided into categories based on their average TV time: **frequently exposed** (>0 hr/day TV for children <2 yr, and >2 hr/day TV for children ≥2 yr), or **infrequently exposed** (no TV for children <2 yr, and ≤2 hr/day for children ≥2 yr).	**Motor skill development** [gross and fine; categorized as "**typical**" (>15th percentile) or "**delayed**" (≤15th percentile)] assessed by the Peabody Developmental Motor Scales-second edition (PDMS-2; 6 subtests: reflexes, stationary, locomotion, object manipulation, grasping, and visual-motor integration).	Children with **delayed motor skill development** spent more **time watching TV** compared to children with **typical motor skill development** (116.9 vs 64.4 min/day; t = 2.3, p < 0.05).<br><br>Children who were **frequently exposed** to TV were more likely to have **delayed motor skill development** than those who were **infrequently exposed** (OR = 3.7, 95% CI: 1.5, 9.3).<br>% of sample with **typical** and **delayed motor skill development** in those	Analyses were multivariate, but covariates were not specified.

					**frequently vs infrequently exposed** respectively: typical (69.3 vs 84.0%), delayed (30.7 vs 16.0%) (X^2 = 4.5, p < 0.05).	
Johansson et al. 2015 [21]; Sweden; Early Stockholm Obesity Prevention Project (Early STOPP)	Cross-sectional	N = 123<br><br>Mean age: 2.03 yr<br>Toddlers	**Total sedentary time** min/day; ≤89 counts/5 sec epoch) measured objectively by accelerometer (Actigraph, GT3X+ worn on the wrist).<br><br>**Number of 30 min bouts of sedentary behaviour** and **total time in 30 min bouts of sedentary behaviour** (number and min/day; 30 min of ≤89 counts/5 sec with a 1 min interruption of >89 counts/5 sec allowed).	**Motor skills** [“neurological optimality score”; scores range from 0 to 58; higher scores are better; categories: “low” (scores of <53) and “normal” (scores of ≥53)].	**Total sedentary time** was not different between children with **low** (416, SD = 48 min/day) and **normal** (434, SD = 47 min/day) **motor skills** (p = 0.09).<br><br>The **number of 30 min bouts of sedentary behaviour** was not different between children with **low** (2.4, SD = 1.3 bouts) and **normal** (2.5, SD = 1.2 bouts) **motor skills** (p = 0.67), nor was the **total time in 30 min bouts of sedentary behaviour** (low: 143, SE = 67 min; normal: 157, SD = 83 min; p = 0.44).	N/A
Williams et al. 2008 [23]; USA; Children’s Activity and Movement in Preschool Study (CHAMPS)	Cross-sectional	N = 198<br><br>Approximate age: 3 yr and 4 yr<br>Pre-schoolers	**Total sedentary time** hr/day and % time; <37.5 counts/15 sec epoch) measured objectively by accelerometer (Actigraph, 7164).	**Locomotor skills** (score out of 73) and **object control skills** (score out of 80), and **total motor skills** (score out of 153) assessed by observers using the CHAMPS Motor Skill Protocol (CMSP).	**Total sedentary time** was not associated with **locomotor skills** (*r* = -0.10**)**, **object control skills** (*r* = -0.09), or **total motor skills** (*r* = -0.11) (all p > 0.05).<br><br>**Total % sedentary time** did not vary by tertile of	Sex, BMI, race, and parent education, and preschool center (as a random variable).

					**total motor skill** performance (tertiles: low, 56.1, SE = 0.9; intermediate, 54.5, SE = 0.9; high, 53.7, SE = 0.9; p > 0.05) or **object control skills** (tertiles: low, 55.7, SE = 0.9; intermediate, 54.5, SE = 0.9; high, 53.9, SE = 1.0; p > 0.05).<br><br>Children in the highest tertile of **locomotor skills score** had significantly less **total % sedentary time** than children in the low and intermediate tertiles (tertiles: low, 55.7, SE = 09; intermediate, 55.6, SE = 0.9; high, 53.1 SE = 0.9; p < 0.05).	

B, unstandardized beta; β, standardized beta; CHAMPS, Children's Activity and Movement in Preschool Study; CI, confidence interval; CMSP, CHAMPS Motor Skill Protocol; Early STOPP, Early Stockholm Obesity Prevention Project; INFANT, Melbourne Infant Feeding, Activity and Nutrition Trial Program; NLSY, National Longitudinal Survey for Children and Youth study; PDMS-2, Peabody Developmental Motor Scales-second edition; PPVT-III, Peabody Picture Vocabulary Test III; QLSCD, Quebec Longitudinal Study of Child Development; SD, standard deviation; T1-T4, Time 1 to Time 4; TV, television; WRAVMA, Wide-Range Assessment of Visual Motor Ability.

Table S3. Summary of findings for psychosocial health outcomes

Reference; Country; Larger Study/Cohort Name	Study Design	Sample	Exposure	Outcome	Main Findings	Covariates included in model (if applicable)
Study Design: RCT						
Yilmaz et al. 2014 [1]; Turkey	Randomized Controlled Trial (RCT)	N = 412<br>Intervention,<br>n = 211;<br>Control,<br>n = 201<br><br>Mean age:<br>Intervention, 3.52 yr<br>Control, 3.49 yr<br>Pre-schoolers	**Intervention:** 3 printed materials and interactive CDs and one counselling call, intending to decrease screen time.<br>**Control:** Usual care; unaware of counselling interventions.<br><br>**Screen time** (min/day) was assessed by parent-report 1-week time-record diary (unpublished).<br><br>The intervention was 8 weeks in duration, with follow-up at 2, 6 and 9 months post-intervention.	**Aggressive behavior** (units not indicated) and **delinquent behavior** (units not indicated) assessed by parent-report using the Child Behavior Checklist (CBCL).	**Screen time** was significantly lower in the **intervention** vs **control** group at 2, 6 and 9 month follow-up post-intervention (mean ± SD: 2 month: 39.48 ± 16.36 vs 86.64 ± 21.63 min/day; 6 month: 24.72 ± 4.45 vs 84.95 ± 14.77 min/day; 9 month: 21.15 ± 6.12 vs 93.96 ± 18.84 min/day; all $p < 0.001$).<br><br>**Aggressive behavior** was not different between groups at baseline (mean ± SD: intervention, 6.94 ± 1.66; control, 7.17 ± 1.52; $p = 0.283$), but significantly lower in the **intervention** vs **control** group at 9-months post-intervention (mean ± SD: intervention, 3.35 ± 1.46; control, 3.85 ± 1.38; $p = 0.001$).<br><br>**Delinquent behavior** was not different between groups at baseline (mean ± SD: intervention, 3.02 ± 1.23; control, 3.02 ± 1.24;	N/A

					p=0.858), but significantly lower in the intervention vs control group at 9-months post-intervention (mean ± SD: intervention, 3.45 ± 1.56; control, 3.83 ± 0.95; p=0.006).	
Study Design:<br>Longitudinal						
Cheng et al. 2010 [72]; Japan<br><br>Japan Children's Study	Longitudinal (~1 yr follow-up)	N = 302<br><br>Approximate age:<br>T1, 18 mo (~1.5 yr)<br>T2, 30 mo (~2.5 yr)<br>Toddlers	**TV time** (hr/day) at ages ~18 and 30 mo assessed by parent-report questionnaire (unpublished).<br><br>**TV time patterns** from age ~18 mo to age ~30 mo [categories: **high-high**, ≥4 hr/day at 18 and 30 mo; **high-low**, ≥4 hr/day at 18 mo and <4 hr/day at 30 mo; **low-high**, <4 hr/day at 18 mo and ≥4 hr/day at 30 mo; **low-low** <4 hr/day at 18 and 30 mo] assessed by parent-report questionnaire (unpublished).	**Emotional symptoms**, **conduct problems**, **peer-problems,** and **prosocial behaviour** (all measured on a scale from 0 to 10; higher numbers are unfavourable for all except prosocial behaviour) assessed by parent-report using the Japanese version of the Strengths and Difficulties Questionnaire (SDQ).	**Emotional symptoms** at age ~30 mo were not different between categories of **TV time** at age ~18 mo (mean scores by TV time categories: *unadjusted*, <1hr/day, 1.83; 95% CI: 1.4, 2.3; ≥1 to <3 hr/day, 2.03, 95% CI: 1.7, 2.4; ≥3 to <4 hr/day, 2.11, 95% CI: 1.7, 2.6; ≥4 hr/day, 1.79, 95% CI: 1.4, 2.2; p-value for mean differences, p = 0.599).<br><br>**Conduct problems** at age ~30 mo were not different between categories of **TV time** at age ~18 mo (mean scores by TV time categories: *unadjusted*, <1hr/day, 3.07; 95% CI: 2.5, 3.7; ≥1 to <3 hr/day, 3.17, 95% CI: 2.8, 3.5; ≥3 to <4 hr/day, 3.11, 95% CI: 2.6, 3.6; ≥4 hr/day, 3.22, 95% CI: 2.8, 3.7; p-value for mean differences, p = 0.973).	Child sex, birth weight, gestational age, birth order, maternal education and family income and maternal stimulation.

					**Peer-problems** at age ~30 mo were not different between categories of **TV time** at age ~18 mo (mean scores by TV time categories: *unadjusted*, <1hr/day, 2.14; 95% CI: 1.6, 2.7; ≥1 to <3 hr/day, 2.17, 95% CI: 1.9, 2.5; ≥3 to <4 hr/day, 2.08, 95% CI: 1.7, 2.5; ≥4 hr/day, 2.28, 95% CI: 1.9, 2.7; p-value for mean differences, p = 0.844).<br><br>There was an unfavourable dose-response relationship between **TV time** at age ~18 mo and **prosocial behaviour** at age ~30 mo in *unadjusted* but not *adjusted* analyses (mean scores by TV time categories: *unadjusted*, <1hr/day, 5.98; 95% CI: 5.2, 6.7; ≥1 to <3 hr/day, 5.42, 95% CI: 5.0, 5.9; ≥3 to <4 hr/day, 5.26, 95% CI: 4.7, 5.8; ≥4 hr/day, 4.65, 95% CI: 4.1, 5.2; p-value for mean differences, p = 0.020; p-value for linear trend for means, p = 0.004; *adjusted*, <1hr/day, 5.80; 95% CI: 4.9. 6.7; ≥1 to <3 hr/day, 5.50, 95% CI: 4.9,	

					6.0; ≥3 to <4 hr/day, 5.21, 95% CI: 4.5, 5.9; ≥4 hr/day, 4.73, 95% CI: 4.2, 5.3; p-value for mean differences, p = 0.132; p-value for linear trend, p = 0.039).<br><br>**Prosocial behaviour** was not different across **TV time patterns** (high-high, 5.00, 95% CI: 4.3, 5.7; high-low, 4.32, 95% CI: 3.4, 5.2; low-high, 5.31, 95% CI: 4.2, 6.4; low-low, 5.48, 95% CI: 5.1, 5.8; p-value for mean differences, p = 0.088).	
Verlinden et al. 2012 [73]; Netherlands<br><br>The Generation R Study	Longitudinal (~1 yr follow-up)	N = 3309<br><br>Approximate age:<br>T1, 24 mo (~2 yr)<br>T2, 36 mo (~3 yr)<br>Toddlers	**TV time** (hr/day) at age ~24 mo and **TV time pattern** (categories: never, continued low, continued moderate, continued high, increased exposure) from age ~24 to ~36 mo, assessed by parent-report questionnaire (unpublished).<br><br>Categories of "change in TV time pattern" were defined as follows:<br>– never: never or less than 0.5 hr/day at both time points;<br>– continued low:0.5-1 hr/day;	**Externalizing problems** (categories: externalizing problems, score ≥18; no externalizing problems, score <18) assessed by parent-report using the CBCL.	**TV time** at age ~24 mo was not associated with the incidence of **externalizing problems** at age ~36 mo (*unadjusted:* OR = 2.24, 95% CI : 0.97, 5.18; *adjusted:* OR = 1.53, 95% CI: 0.62, 3.81).<br><br>**Continued high TV time** and **increased TV time** between ~24 to ~36 mo of age were associated with an increased incidence of **externalizing problems** at age ~36 mo (compared to "never": *unadjusted:* continued low, OR = 1.13, 95% CI: 0.58, 2.23, p = 0.72; continued moderate,	Externalizing symptoms at age 18 mo, and child's age, national origin, sex, day care attendance, maternal and paternal age, maternal and paternal educational level, marital status, monthly income, maternal symptoms of psychiatric disorders, parenting stress, parity.

			– continued moderate:1 hr/day;<br>– high: both continued high (≥0.5 hr/day at 24 mo and ≥1 hr/ day at 36 mo) and increased (<0.5 hr/day at 24 mo and ≥1 hr/ day at 36 mo);<br>– continued high: ≥0.5 hr/day at 24 mo and ≥1 hr/ day at 36 mo;<br>– increased: <0.5 hr/day at 24 mo and ≥1 hr/ day at 36 mo		OR = 1.47, 95% CI: 0.73, 2.93, p = 0.28; high, OR = 2.62, 95% CI: 1.48, 4.66, p = 0.001; continued high, OR = 2.66, 95% CI: 1.47, 4.79, p = 0.001; increased, OR = 2.50, 95% CI: 1.15, 5.41, p = 0.02; *adjusted:* continued low, OR = 1.01, 95% CI: 0.55, 2.57, p = 0.98; continued moderate, OR = 1.20, 95% CI: 0.57, 2.50, p = 0.63; high, OR = 2.00, 95% CI: 1.07, 3.75, p = 0.03; continued high, OR = 2.09, 95% CI: 1.08, 4.01, p = 0.03; increased, OR = 1.90, 95% CI: 1.90, 4.21, p = 0.17).	
Pagani et al. 2010 [9]; Canada<br><br>QLSCD (Quebec Longitudinal Study of Child Development)	Longitudinal (~8 yr follow-up)	N = 1314<br><br>T1, ~Age: 29 mo (~2.4 yr) Toddlers<br><br>T2, ~Age: 53 mo (4.4 yr) Pre-schoolers<br><br>T3, Grade 4 (in Grade 4 in Quebec, children are aged 9 to 10; School-age)	**TV time** (hr/week) at age 29 mo assessed by parent-report questionnaire (unpublished).<br><br>**Change in TV time** (hr/week) from age 29 mo to 53 mo.	**Victimization** (no units) assessed by teacher-report using the Social Behaviour Questionnaire (SBQ).	**TV time** at age 29 months was unfavourably associated with **victimization** in Grade 4 (age ~9-10 yr; β = 0.10, SE = 0.01; B = 0.03, 95% CI: 0.01, 0.05; p < 0.0001).<br><br>The **change in TV time** from age 29 mo to 53 mo was not associated with **victimization** in Grade 4 (age ~9-10 yr; β = 0.06, SE = 0.01; B = 0.01, 95% CI: -0.01, 0.03; p ≤ 0.05).	**TV time:** Change in TV time, concurrent TV time, sex, temperament, cognitive ability, impulsivity, emotional distress, physical aggression, *hours of sleep*, maternal education, family makeup, family functioning.<br><br>**Change in TV time:** Same as above, except inclusion of "baseline TV time" instead of "change in TV time".
Pagani et al. 2013 [69]; Canada	Longitudinal (3 yr follow-up)	N = 1999<br><br>Approximate Age:	**TV time** (hr/day) at age 29 mo assessed by parent-report questionnaire	**Anxiety, physical aggression, prosocial behaviour,** and	**TV time** at age ~29 mo was unfavourably associated **victimization**	Maternal education, early stimulation of literacy, early childhood

QLSCD		T1, 29 mo (~2.4 yr)<br>Toddlers<br><br>T2, 65 mo (~5.4 yr)<br>School-age	(unpublished).	**victimization** (scores ranging from 1 to 10; higher scores indicate a greater degree of the factor) a assessed by teachers using the SBQ.	**by classmates** (B = 0.008, 95% CI: 0.002, 0.014, p = 0.005) at age ~65 mo.<br><br>**TV time** at age ~29 mo was not associated with **anxiety, physical aggression,** or **prosocial behaviour** at age ~65 mo (values not reported).	temperament, family functioning.
Watt et al. 2015 [74]; Canada; Quebec Longitudinal Study of Child Development (QLSCD)	Longitudinal (~9.5 yr follow-up)	N = 1314<br><br>Approximate age:<br>T1, 29 mo (~2.4 yr)<br>Toddlers<br><br>T2, Grade 6 (~12 yr)<br>School-age	**TV time** (hr/day; categories: ≤2 hr/day, >2 hr/day) assessed by parent-report questionnaire (unpublished).	**Victimization** (scores from 0 to 10; higher values indicate more victimization) assessed by self-report with the Social Behavior Questionnaire.<br><br>Children were categorized as being in the **high victimization group** if their scores were beyond 71.2% of the sample (cutoff selected using the frequency distribution).	**TV time** at age ~2.4 yr was unfavourably associated with **victimization** at age ~12 yr (B = 0.031, SE = 0.01, 95% CI: 0.014, 0.042, p = 0.001; β = 0.11).<br><br>Children who had **>2 hr/day TV time** were more likely than children who had **≤2 hr/day TV time** to show a 0.086 SD unit increase in victimization (B = 0.428, 95% CI: 0.159, 0.696, p = 0.002), and were 54% more likely to be in the **high victimization group**.	Gender, factors at age ~2.4 yr (externalizing behaviour, cognitive ability, emotional distress), factors at age ~12 yr (concurrent TV time, family configuration, family functioning, family income, maternal education).
Mistry et al. 2007 [75]; USA; Healthy Steps for Young Children	Longitudinal (~3 yr follow-up)	N = 2707<br><br>Approximate age:<br>T1: 30 to 33 mo (2.5 to 2.8 yr)<br>Toddlers<br><br>T2: 5.5 yr	**TV time** (hr/day, and categories: >2 hr/day, ≤2 hr/day) assessed by parent-report interview (unpublished format).	The following were assessed by parent-report using the Child Behavior Checklist (CBCL):<br>**emotionally reactive** (scale from 0 to 18),<br>**anxious or depressed** (scale from 0 to 16),,	**TV time** at age ~2.5 yr was favourably associated with **emotionally reactivity** scores (*unadjusted:* β = -0.33, 95% CI: -0.59, -0.07, p ≤ 0.05; *adjusted:* -β = -0.43, 95% CI: -0.69, -0.17, p ≤	Maternal demographic characteristics (age at child's birth, race, ethnicity, marital status, employment, education), child's gender, parity, household income, child's health status, maternal

		School-age		**aggressive behavior** (scale from 0 to 38), and **externalizing scale** (scale from 0 to 48; sum of attention problems and aggressive behavior scores). Higher scores indicate more problems.<br><br>The following were assessed by parent-report using the Social Skills Rating System: **cooperation, assertion, responsibility, self-control,** and **total social skills** (a composite of the other items). All items rated on a scale from 0 to 20; higher scores indicate greater social skills.	0.01), but was not associated with **anxious or depressed** scores (*unadjusted:* β = 0.01, 95% CI: -0.25, 0.25; *adjusted:* β = -0.22, 95% CI: -0.47, 0.04; both p > 0.05), , **aggressive behavior** (*unadjusted:* β = 0.11, 95% CI: -0.57, 0.80; *adjusted:* β = -0.30, 95% CI: -0.98, 0.39; both p > 0.05), or **externalizing** (*unadjusted:* β = 0.25, 95% CI: -0.56, 1.06; *adjusted:* β = -0.37, 95% CI: -1.18, 0.43; both p > 0.05) at age ~5.5 yr.<br><br>**TV time** at age ~2.5 yr was unfavourably associated with **cooperation** and **self-control** at age ~5.5 yr in *unadjusted* (cooperation: β = -0.39, 95% CI: -0.73, -0.05; self-control: β = -0.46, 95% CI: -0.82, -0.10; both p ≤ 0.05) but not *adjusted* (cooperation: β = -0.19, 95% CI: -0.52, 0.15; self-control: β = -0.16, 95% CI: -0.52, 0.20; both p > 0.05) analyses.<br>**TV time** at age ~2.5 yr was not associated with **assertion** (*unadjusted:* β = -0.07, 95% CI: -0.41, 0.26; *adjusted:* β = 0.16,	depressive symptoms, parental involvement in child's activities, intervention status (note: the intervention is designed to enhance the delivery of developmental services for families).

					95% CI: -0.18, 0.50; both p > 0.05), **responsibility** (*unadjusted:* β = 0.08, 95% CI: -0.43, 0.27; *adjusted:* β = 0.02, 95% CI: -0.33, 0.36; both p > 0.05) or **total social skills** (*unadjusted:* β = -0.97, 95% CI: -2.11, 0.16; *adjusted:* β = -0.16, 95% CI: -1.26, 0.94; both p > 0.05) at age ~5.5 yr.	
Verlinden et al. 2014 [76]; Netherlands<br><br>Generation R Study	Longitudinal (~4 yr follow-up)	N = 3423<br><br>Approximate age at baseline:<br>T1, assessed at least twice between ages 2 to 5 yr<br>Pre-schoolers<br><br>Mean age at follow-up:<br>T2: Teacher report, 6.8 yr; Self/proxy-report, 7.6 yr<br>School-age	**TV time** (hr/day) at age 2 yr, age 3 yr, age 4 yr, and at least twice between ages 2-5 (combined for analysis) assessed by parent-report questionnaire (unpublished).<br><br>TV time was divided into four categories for analysis for 2 year olds: never, <0.5 hr/day, 0.5-1 hr/day, >1 hr/day; and five categories for 3, 4 and 5 year olds: never, <0.5 hr/day, 0.5-1 hr/day, 1-2 hr/day, >2 hr/day. TV time measures were also combined into a latent variable (categories: **low, mid-low, mid-high, high**) to reflect **TV time patterns** throughout ages 2-5 years.	**Risk of being a bully, victim, bully-victim** (categories: bully, victim, bully-victim, uninvolved in bullying) assessed by teacher-report questionnaire (unpublished) and by self- and proxy-report (peer nomination; unpublished questionnaire) in Grades 1-2.	*Unadjusted and Adjusted Analyses:*<br><br>**High TV time at ages 2-5 yr** was unfavourably associated with **teacher-report of being a bully** at age ~7 yr compared to **low TV time** in *unadjusted* but not *adjusted analyses*, but there was no difference for **mid-low** or **mid-high TV time** in either *unadjusted* or *adjusted* analyses (compared to low TV time: *unadjusted:* mid-low, OR = 1.07, 95% CI: 0.79, 1.44, p = 0.66; mid-high, OR = 1.35, 95% CI: 0.98, 1.84, p = 0.07; high, OR = 1.74, 95% CI: 1.22, 2.50, p = 0.002; *adjusted:* mid-low, OR = 1.0, 95% CI: 0.74, 1.35, p = 0.99; mid-high, OR = 1.15, 95% CI: 0.83, 1.59, p = 0.42; high, OR = 1.27, 95% CI:	Child gender, age, national origin, internalizing and externalizing problems and day-care attendance, and maternal age, parity, education, income, marital status, maternal symptoms of depression, parenting stress.

					0.86, 1.86, p = 0.23).<br><br>**TV time patterns at ages 2-5 yr** were not associated with **self- or proxy-report of being a bully** at age ~7 yr (compared to low TV time: *unadjusted:* mid-low, OR = 0.85, 95% CI: 0.51, 1.43, p = 0.54; mid-high, OR = 1.28, 95% CI: 0.75, 2.18, p = 0.37; high, OR = 1.33, 95% CI: 0.66, 2.65, p = 0.43; *adjusted:* mid-low, OR = 0.68, 95% CI: 0.39, 1.18, p = 0.17; mid-high, OR = 0.86, 95% CI: 0.47, 1.55, p = 0.61; high, OR = 0.71, 95% CI: 0.33, 1.54, p = 0.39).<br><br>**High TV time at ages 2-5 yr** was unfavourably associated with **teacher-report of being a victim** at age ~7 yr compared to **low TV time,** but there was no difference for **mid-low** or **mid-high TV time** (compared to low TV time: *unadjusted:* mid-low, OR = 1.17, 95% CI: 0.71, 1.92, p = 0.54; mid-high, OR = 1.11, 95% CI: 0.64, 1.95, p = 0.71; high, OR = 2.38, 95% CI: 1.33, 4.28, p = 0.004; *adjusted:* mid-low, OR = 1.16, 95% CI: 0.70, 1.91, p = 0.57;	

					mid-high, OR = 1.02, 95% CI: 0.57, 1.82, p = 0.96; high, OR = 1.80, 95% CI: 0.94, 3.41, p = 0.07).<br><br>**TV time patterns at ages 2-5 yr** were not associated with **self- or proxy-report of being a victim** at age ~7 yr (compared to low TV time: *unadjusted:* mid-low, OR = 0.91, 95% CI: 0.63, 1.32, p = 0.64; mid-high, OR = 0.98, 95% CI: 0.61, 1.56, p = 0.93; high, OR = 1.10, 95% CI: 0.57, 2.13, p = 0.77; *adjusted:* mid-low, OR = 0.88, 95% CI: 0.61, 1.28, p = 0.51; mid-high, OR = 0.83, 95% CI: 0.50, 1.37, p = 0.47; high, OR = 0.85, 95% CI: 0.43, 1.68, p = 0.63).<br><br>**High TV time** and **mid-high TV time at ages 2-5 yr** were unfavourably associated with **teacher-report of being a bully-victim** at age ~7 yr compared to **low TV time** in *unadjusted* but not *adjusted analyses*, and there was no difference for **mid-low TV time** in *unadjusted* or *adjusted analyses* (compared to low TV time: *unadjusted:* mid-low, OR = 1.21, 95% CI:	

					0.88, 1.65, p = 0.24; mid-high, OR = 1.73, 95% CI: 1.25, 2.40, p = 0.001; high, OR = 2.11, 95% CI: 1.42, 3.13, p < 0.001; *adjusted:* mid-low, OR = 1.08, 95% CI: 0.79, 1.48, p = 0.64; mid-high, OR = 1.31, 95% CI: 0.93, 1.85, p = 0.13; high, OR = 1.35, 95% CI: 0.88, 2.08, p = 0.17).<br><br>**High TV time at ages 2-5 yr** was unfavourably associated with **self- or proxy-report of being a bully-victim** at age ~7 yr compared to **low TV time** in *unadjusted* but not *adjusted analyses*, and there was no difference for **mid-low** or **mid-high TV time** in *unadjusted* or *adjusted analyses* (compared to low TV time: *unadjusted:* mid-low, OR = 1.71, 95% CI: 0.88, 3.32, p = 0.11; mid-high, OR = 1.95, 95% CI: 0.99, 3.83, p = 0.05; high, OR = 3.68, 95% CI: 1.75, 7.74, p = 0.001; *adjusted:* mid-low, OR = 1.36, 95% CI: 0.70, 2.65, p = 0.37; mid-high, OR = 1.21, 95% CI: 0.59, 2.46, p = 0.60; high, OR = 1.60, 95% CI: 0.72, 3.55, p = 0.25).	

					Adjusted analyses only:<br><br>**TV time at age 2 yr** was not associated with **being a bully** at age ~7 yr (compared to "never": *teacher-report:* <0.5 hr/day, OR = 1.29, 95% CI: 0.82, 2.09, p = 0.29; 0.5-1 hr, OR = 1.12, 95% CI: 0.69, 1.82, p = 0.65; >1 hr, OR = 1.34, 95% CI: 0.80, 2.27, p = 0.27; *self- or proxy-report:* <0.5 hr, OR = 1.18, 95% CI: 0.54, 2.58, p = 0.63; 0.5-1 hr, OR = 0.98, 95% CI: 0.42, 2.27, p = 0.20; >1 hr, OR = 1.22, 95% CI: 0.48, 3.09, p = 0.84).<br><br>**TV time at age 2 yr** was not associated with **being a victim** at age ~7 yr (compared to "never": *teacher-report:* <0.5 hr/day, OR = 2.69, 95% CI:0.81, 8.97, p = 0.113; 0.5-1 hr, OR = 1.97, 95% CI: 0.58, 6.64, p = 0.28; >1 hr, OR = 3.38, 95% CI: 0.99, 11.56, p = 0.05; *self- or proxy-report:* <0.5 hr, OR = 1.06, 95% CI: 0.54, 2.07, p = 0.86; 0.5-1 hr, OR = 0.92, 95% CI: 0.46, 1.86, p = 0.82; >1 hr, OR = 0.89, 95% CI: 0.39,	

					2.06, p = 0.79).<br><br>**TV time at age 2 yr** was not associated with **being a bully-victim** at age ~7 yr (compared to “never”: *teacher-report:* <0.5 hr/day, OR = 0.64, 95% CI: 0.39, 1.04, p = 0.07; 0.5-1 hr, OR = 0.82, 95% CI: 0.50, 1.34, p = 0.43; >1 hr, OR = 0.85, 95% CI: 0.50, 1.42, p = 0.53; *self- or proxy-report:* <0.5 hr, OR = 1.72, 95% CI: 0.52, 5.75, p = 0.38; 0.5-1 hr, OR = 1.82, 95% CI: 0.55, 6.02, p = 0.33; >1 hr, OR = 1.40, 95% CI: 0.39, 5.02, p = 0.60).<br><br>**TV time at age 3 yr** was not associated with **being a bully** at age ~7 yr (compared to “never” and <0.5 hr/day: *teacher-report:* 0.5-1 hr, OR = 0.95, 95% CI: 0.71, 1.26, p = 0.71; 1-2 hr, OR = 1.09, 95% CI: 0.80, 1.50, p = 0.57; >2 hr, OR = 1.00, 95% CI: 0.65, 1.53, p = 0.98; *self- or proxy-report:* 0.5-1 hr, OR = 0.66, 95% CI: 0.39, 1.12, p = 0.13; 1-2 hr, OR = 0.85, 95% CI: 0.48, 1.51, p = 0.57; >2 hr, OR = 0.72, 95% CI: 0.32, 1.59,	

					p = 0.42).<br><br>**TV time at age 3 yr** was not associated with **being a victim** at age ~7 yr (compared to "never" and <0.5 hr/day: *teacher-report:* 0.5-1 hr, OR = 1.51, 95% CI: 0.89, 2.56, p = 0.12; 1-2 hr, OR = 1.36, 95% CI: 0.75, 2.45, p = 0.31; >2 hr, OR = 1.80, 95% CI: 0.86, 3.75, p = 0.12; *self- or proxy-report:* 0.5-1 hr, OR = 0.80, 95% CI: 0.53, 1.21, p = 0.29; 1-2 hr, OR = 0.87, 95% CI: 0.51, 1.48, p = 0.61; >2 hr, OR = 0.61, 95% CI: 0.24, 1.55, p = 0.30).<br><br>**TV time at age 3 yr** was not associated with **being a bully-victim** at age ~7 yr (compared to "never" and <0.5 hr/day: *teacher-report:* 0.5-1 hr, OR = 0.97, 95% CI: 0.71, 1.31, p = 0.83; 1-2 hr, OR = 1.30, 95% CI: 0.93, 1.82, p = 0.12; >2 hr, OR = 1.17, 95% CI: 0.74, 1.87, p = 0.50; *self- or proxy-report:* 0.5-1 hr, OR = 1.40, 95% CI: 0.77, 2.55, p = 0.27; 1-2 hr, OR = 1.32, 95% CI: 0.65, 2.69, p = 0.45; >2 hr, OR =	

					2.06, 95% CI: 0.81, 5.25, p = 0.13).<br><br>**TV time at age 4 yr** was not associated with **being a bully** at age ~7 yr (compared to <0.5 hr/day: *teacher-report:* 0.5-1 hr, OR = 0.80, 95% CI: 0.54, 1.18, p = 0.27; 1-2 hr, OR = 0.80, 95% CI: 0.53, 1.21, p = 0.30; >2 hr, OR = 0.99, 95% CI: 0.61, 1.63, p = 0.98; *self- or proxy-report:* 0.5-1 hr, OR = 0.84, 95% CI: 0.84, 1.56, p = 0.58; 1-2 hr, OR = 0.69, 95% CI: 0.58, 2.31, p = 0.69; >2 hr, OR = 0.97, 95% CI: 0.41, 2.29, p = 0.95).<br><br>**TV time at age 4 yr** was not associated with **being a victim** at age ~7 yr (compared to <0.5 hr/day: *teacher-report:* 0.5-1 hr, OR = 0.70, 95% CI: 0.40, 1.22, p = 0.23; 1-2 hr, OR = 0.63, 95% CI: 0.33, 1.20, p = 0.56; >2 hr, OR = 1.34, 95% CI: 0.67, 2.69, p = 0.40; *self- or proxy-report:* 0.5-1 hr, OR = 2.03, 95% CI: 1.02, 4.07, p = 0.04; 1-2 hr, OR = 1.46, 95% CI: 0.70, 2.98, p = 0.29; >2 hr, OR = 2.12, 95% CI: 0.86,	

					5.22, p = 0.10).<br><br>**TV time at age 4 yr** was not associated with **being a bully-victim** at age ~7 yr (compared to <0.5 hr/day: *teacher-report:* 0.5-1 hr, OR = 1.03, 95% CI: 0.66, 1.60, p = 0.90; 1-2 hr, OR = 1.10, 95% CI: 0.69, 1.75, p = 0.68; >2 hr, OR = 1.28, 95% CI: 0.73, 2.24, p = 0.39; *self- or proxy-report:* 0.5-1 hr, OR = 0.59, 95% CI: 0.33, 1.06, p = 0.08; 1-2 hr, OR = 0.61, 95% CI: 0.32, 1.16, p = 0.13; >2 hr, OR = 0.85, 95% CI: 0.38, 1.93, p = 0.70).	
Zimmerman et al. 2005 [77]; USA<br><br>National Longitudinal Survey of Youth 1979 Children and Youth Adults (NLSY-Child)	Longitudinal (~5 yr follow-up)	N = 1266<br><br>Approximate age: T1, "survey year occurring closest to the 4-year birthday"<br>Pre-schoolers<br><br>Mean age: T2, 9.19 yr<br>School-age	**TV time** (hr/day) assessed by parent-report questionnaire (unpublished).	**Bullying** (categories: bullies, nonbullies) assessed by parent-report questionnaire (unpublished).	**TV time** at age ~4 yr was unfavourably associated with **being a bully** at age ~9 yr (for each hr/day TV time: OR = 1.06, 95% CI: 1.02, 1.11).	Age, sex, race or ethnicity, parents' income and education.
Hinkley et al. 2014 [78]; Belgium, Cyprus, Estonia, Germany, Hungary, Italy, Spain, Sweden	Longitudinal (~2 yr follow-up)	N = 3604<br><br>T1, 4.3 yr<br>Pre-schoolers<br><br>T2, 6.3 yr<br>School-age	**TV time** (approximate min/hr) on weekdays and weekend days, and **time spent e-gaming or on a computer** (approximate min/hr) on weekdays and weekend days, assessed	**Emotional problems** (e.g., often worried, unhappy, depressed), and **peer problems** (e.g., rather solitary, picked on/bullied), assessed by parent-report using the	**TV time** at age 4.3 yr was not associated with being **at-risk for emotional problems** at age 6.3 yr (weekdays: *boys:* OR = 1.2, 95% CI: 0.9, 1.5; *girls:* OR = 1.3, 95% CI:	Region, age, socioeconomic position, body mass index, clustering by centre of recruitment, baseline levels of SDQ or KINDL outcome variable under

Identification and Prevention of Dietary-and Lifestyle-Induced Health Effects in Children and Infants (IDEFICS)			by parent-report questionnaire (adapted from the "Generation M-study" [79]).	SDQ. Items are scored as "healthy" (normal) or "at-risk" (borderline and abnormal).<br><br>**Self-esteem** (e.g., proud of self peased with self), **emotional well-being** (e.g., had fun, was scared), **family functioning** (e.g., felt fine at home, got on well with parents), and **social networks** (e.g., liked by other children, got on well with friends) assessed by parent-report using the KINDL^{R} (Questionnaire for Measuring Health-Related Quality of Life in Children and Adolescents-Revised Version). Each item receives a total scaled score of 100 possible points (higher scores are more favourable); scores ≤25th percentile considered "at risk", and scores >25th percentile considered "not at risk".	1.0, 1.7; weekend days: *boys*: OR = 1.0, 95% CI: 0.8, 1.3; *girls:* OR = 1.3, 95% CI: 1.0, 1.6; all p > 0.05).<br><br>**TV time** at age 4.3 yr was not associated with being **at-risk for peer problems** at age 6.3 yr (weekdays: *boys:* OR = 1.0, 95% CI: 0.8, 1.3; *girls:* OR = 1.1, 95% CI: 0.9, 1.5; weekend days: *boys:* OR = 1.0, 95% CI: 0.9, 1.2; *girls:* OR = 1.1, 95% CI: 1.0, 1.5; all p > 0.05).<br><br>**TV time** at age 4.3 yr was not associated with being **at-risk for self-esteem problems** (weekdays: *boys:* OR = 1.1, 95% CI: 0.9, 1.3; *girls:* OR = 1.0, 95% CI: 0.8, 1.3; weekend days: *boys:* OR = 1.1, 95% CI: 0.9, 1.3; *girls:* OR = 1.0, 95% CI: 0.8, 1.1; all p > 0.05).<br><br>**TV time** at age 4.3 yr was not associated with being **at-risk for emotional well-being problems** at age 6.3 yr (weekdays: *boys:* OR = 1.2, 95% CI: 1.0, 1.4; *girls:* OR = 1.1, 95% CI: 0.9, 1.4; weekend days: *boys:* OR = 1.1, 95%	investigation

					CI: 1.0, 1.3; *girls:* OR = 1.1, 95% CI: 0.9, 1.3; all $p > 0.05$).<br><br>**TV time on weekdays** at age 4.3 yr was unfavourably associated with being **at-risk for family functioning problems** at age 6.3 yr (*boys:* OR = 1.2, 95% CI: 1.0, 1.5; *girls:* OR = 1.3, 95% CI: 1.0, 1.6; both $p > 0.05$). **TV time on weekend days** at age 4.3 yr was unfavourably associated with being **at risk for family functioning problems** at age 6.3 yr in *girls* (OR = 1.3, 95% CI: 1.0, 1.5, $p < 0.05$) but not *boys* (OR = 1.1, 95% CI: 0.9, 1.3, $p > 0.05$).<br><br>**TV time** at age 4.3 yr was not associated with being **at-risk for social functioning problems** at age 6.3 yr (weekdays: *boys:* OR = 1.2, 95% CI: 1.0, 1.4; *girls:* OR = 0.9, 95% CI: 0.7, 1.1; weekend days: *boys:* OR = 1.0, 95% CI: 0.9, 1.2; *girls:* OR = 1.1, 95% CI: 0.9, 1.3; all $p > 0.05$).<br><br>**Time spent e-gaming or**	

					**on a computer on weekdays** at age 4.3 yr was unfavourably associated with being **at-risk for emotional problems** at age 6.3 yr in *girls* (OR = 2.0, 95% CI: 1.0, 4.0, $p < 0.05$) but not *boys* (OR = 1.3, 95% CI: 0.8, 2.1, $p > 0.05$). **Time spent e-gaming or on a computer on weekend days** at age 4.3 yr was not associated with being **at-risk for emotional problems** at age 6.3 yr (*boys:* OR = 1.0, 95% CI: 0.7, 1.4; *girls:* OR = 1.1, 95% CI: 0.7 1.8; both $p > 0.05$).<br><br>**Time spent e-gaming or on a computer** at age 4.3 yr was not associated with being **at-risk for peer problems** at age 6.3 yr (weekdays: *boys:* OR = 0.8, 95% CI: 0.5, 1.3; *girls:* OR = 1.9, 95% CI: 1.0, 3.8; weekend days: *boys:* OR = 0.9, 95% CI: 0.6, 1.2; *girls:* OR = 1.2, 95% CI: 0.8, 1.8; all $p > 0.05$).<br><br>**Time spent e-gaming or on a computer** at age 4.3 yr was not associated with being **at-risk for self-**	

					**esteem problems** at age 6.3 yr (weekdays: *boys:* OR = 1.0, 95% CI: 0.7, 1.4; *girls:* OR = 1.0, 95% CI: 0.6, 1.9; weekend days: *boys:* OR = 1.1, 95% CI: 0.8, 1.4; *girls:* OR = 1.0, 95% CI: 0.7, 1.4; all p > 0.05).<br><br>**Time spent e-gaming or on a computer** at age 4.3 yr was not associated with being **at-risk for social well-being problems** at age 6.3 yr (weekdays: *boys:* OR = 1.1, 95% CI: 0.8, 1.5; *girls:* OR = 1.2, 95% CI: 0.7, 2.0; weekend days: *boys:* OR = 1.0, 95% CI: 0.8, 1.3; *girls:* OR = 0.9, 95% CI: 0.7, 1.3; all p > 0.05).<br><br>**Time spent e-gaming or on a computer** at age 4.3 yr was not associated with being **at-risk for family functioning problems** at age 6.3 yr (weekdays: *boys:* OR = 1.0, 95% CI: 0.6, 1.4; *girls:* OR = 0.9, 95% CI: 0.5, 1.7; weekend days: *boys:* OR = 0.8, 95% CI: 0.6, 1.0; *girls:* OR = 1.0, 95% CI: 0.7, 1.5; all p > 0.05).<br><br>**Time spent e-gaming or**	

					**on a computer** at age 4.3 yr was not associated with being **at-risk for social functioning problems** at age 6.3 yr (weekdays: *boys:* OR = 0.7, 95% CI: 0.5, 1.1; *girls:* OR = 0.8, 95% CI: 0.5, 1.5; weekend days: *boys:* OR = 0.9, 95% CI: 0.7, 1.1; *girls:* OR = 0.8, 95% CI: 0.6, 1.2; all p > 0.05).	
Study Design:<br>Cross-sectional						
Intusoma et al. 2013 [80]; Thailand<br><br>Prospective Cohort Study of Thai Children (PCTC) cohort study	Cross-sectional	N = 3802<br><br>Approximate age:<br>T1, 1 yr<br>T2, 3 yr<br>Toddlers	**TV time** (hr/day) at ages 1 yr and 3 yr (combined for analysis), assessed by parent-report questionnaire (unpublished).	**Social-emotional competence** (categories: low, not low) at ages 1 yr and 3 yr (combined for analysis) assessed by parent-report interview using the Modified Infant-Toddler Social and Emotional Assessment (MIT-SEA) instrument.	**TV time** was favourably associated with **social-emotional competence**, but the beneficial effect diminished or disappeared when TV duration exceeded 120 min/day (compared to no TV time: 1-30 min/day, OR = 0.86, 95% CI: 0.70, 1.06, p > 0.05; 31-60 min/day, OR = 0.64, 95% CI: 0.46, 0.87, p < 0.01; 61-90 min/day, OR = 0.57, 95% CI: 0.26, 1.28, p > 0.05; 91-120 min/day, OR = 0.53, 95% CI: 0.34, 0.82, p < 0.01; 121-150 min/day, OR = 0.37, 95% CI: 0.05, 2.77, p > 0.05; 151-180 min/day, OR = 0.55, 95% CI: 0.31, 0.98, p < 0.05; >180 min/day, OR = 0.66, 95% CI: 0.35, 1.23, p > 0.05).	Temperament, mother's education, recreational places visited, positive reinforcement, gender, main caregiver, family income, father's education, number of siblings, interactive play, negative reinforcement.

Cheng et al. 2010 [72]; Japan<br><br>Japan Children's Study	Cross-sectional	N = 302<br><br>Approximate age: 30 mo (~2.5 yr)<br>Toddlers	**TV time** (hr/day) assessed by parent-report questionnaire (unpublished).	**Emotional symptoms**, **conduct problems**, **peer-problems,** and **prosocial behaviour** (all measured on a scale from 0 to 10; higher numbers are unfavourable) assessed by parent-report using the SDQ.	**Emotional symptoms** were not different between categories of **TV time** (scores by TV time categories: *unadjusted,* <1hr/day, 2.03; 95% CI: 1.4, 2.6; ≥1 to <3 hr/day, 2.04, 95% CI: 1.8, 2.3; ≥3 to <4 hr/day, 1.81, 95% CI: 1.3, 2.3; ≥4 hr/day, 1.82, 95% CI: 1.4, 2.3; p-value for group mean differences, p = 0.739).<br><br>**Conduct problems** were not different between categories of **TV time** (scores by TV time categories: *unadjusted,* <1hr/day, 2.86; 95% CI: 2.1, 3.6; ≥1 to <3 hr/day, 3.19, 95% CI: 2.9, 3.5; ≥3 to <4 hr/day, 3.44, 95% CI: 2.8, 4.1; ≥4 hr/day, 3.00, 95% CI: 2.6, 3.4; p-value for group mean differences, p = 0.449).<br><br>**Peer-problems** were not different between categories of **TV time** (scores by TV time categories: *unadjusted,* <1hr/day, 2.31; 95% CI: 1.7, 2.9; ≥1 to <3 hr/day, 2.09, 95% CI: 1.8, 2.4; ≥3 to <4 hr/day, 2.50, 95% CI: 1.9, 3.1; ≥4 hr/day, 2.08, 95% CI: 1.7, 2.4; p-	Child sex, birth weight, gestational age, birth order, maternal education and family income and maternal stimulation.

					value for group mean differences, p = 0.359).<br><br>**Prosocial behavior** was not different between categories of **TV time** (scores by TV time categories: *unadjusted*, <1hr/day, 5.71; 95% CI: 4.7, 6.6; ≥1 to <3 hr/day, 5.39, 95% CI: 5.0, 5.8; ≥3 to <4 hr/day, 4.88, 95% CI: 4.2, 5.4; ≥4 hr/day, 5.08, 95% CI: 4.6, 5.7; p = 0.361; *adjusted*, <1hr/day, 5.56; 95% CI: 4.3, 6.5; ≥1 to <3 hr/day, 5.36, 95% CI: 4.9, 5.8; ≥3 to <4 hr/day, 4.96, 95% CI: 4.0, 5.6; ≥4 hr/day, 5.09, 95% CI: 4.6, 5.8; p-value for group mean differences p = 0.718).	
Manganello and Taylor 2009 [81]; USA<br><br>The Fragile Families and Child Wellbeing Study	Cross-sectional	N = 3023<br><br>Mean age: 36 mo (~3 yr)<br>Pre-schoolers	**TV time** (hr/day) assessed by parent-report questionnaire (unpublished).	**Aggression** (score from 0 to 30; higher scores indicate greater aggression) assessed by parent-report using the CBCL/2-3.	**TV time** was unfavourably associated with **aggression** (*unadjusted:* *r* = 0.15, p < 0.001; β = 0.39, p < 0.001; *adjusted:* β = 0.16, 95% CI: 0.08, 0.25, p < 0.001).	Child sex; maternal characteristics: age, nativity, race, education, work hr/wk, relationship status; paternal characteristics: age, education; Household risk characteristics: married at child's birth, income, used food stamps in the past year, additional adults in home, additional children in home.
Teramoto et al. 2005 [82]; Japan	Cross-sectional	N = 670	**TV time** (hr/day) assessed by parent-report	**Total behaviour problems, externalizing**	**TV time** was unfavourably associated	Coffee drinking during pregnancy, number of

		Age range: 36 to 47 mo (~3 to 4 yr)<br>Pre-schoolers	questionnaire (unpublished).	**behaviour problems** (oppositional, aggressive/destructive and attention domains)**,** and **internalizing behaviour problems** (withdrawn behaviour, separation anxiety and anxious/neurotic domains) (units for all were *T* scores; abnormal range *T* ≥64) measured using the Japanese CBCL/2-3.	with **total behaviour problems** (OR = 1.23, 95% CI: 1.06, 1.43, p = 0.011), and **externalizing behaviour problems** (OR = 1.26, 95% CI: 1.08, 1.48, p = 0.004), but not **internalizing behaviour problems** (values not reported).	older brothers/sister, family income.
Miller et al. 2012 [83]; USA	Cross-sectional	N = 150<br><br>Mean age: 3.64 yr<br>Pre-schoolers	**TV time** (categories: 0-1 hr/day, 2-5 hr/day, 6-9 hr/day, 10 or more hr/day) assessed by parent-report interview (unpublished format).	**Aggression toward a sibling** (scale from 1 to 4; higher scores indicate more aggression) assessed by parent-report using the Aggressive Sibling Social Behavior Scale.	**TV time** was not associated with **aggression toward a sibling** (r = 0.14, p > 0.01; β = 0.11, p > 0.05).	Child sex, income, maternal depression, father-child physical aggression, intimate partner violence, hours of violent TV, community violence.
Irwin et al. 2015 [84]; Canada<br><br>Learning Environments' Activity Potential for Preschoolers (LEAPP) study	Cross-sectional	N = 216<br><br>Age range: 2.5 to 5 yr<br>Pre-schoolers	**Total sedentary time** (min/day; collected during the preschool day only; <50 counts/15 sec epoch) measured by accelerometer (Actical).	**Soothability** (unit-weighted scale scores), **sociability** (units not indicated), and **emotionality** (units not indicated) assessed by parent-report using the Child Temperament Questionnaire (CTQ).	**Total sedentary time** was not associated with **soothability** (r = 0.07, 95%CI: -0.06, 0.21), **sociability** (r = -0.01, 95%CI: -0.15, 0.12), or **emotionality** (r = -0.09, 95%CI: -0.22, 0.04).	N/A
Zimmerman et al. 2005 [77]; USA<br><br>NLSY-Child	Cross-sectional	N = 1266<br><br>Approximate age: "survey year occurring closest to the 4-year birthday"<br>Pre-schoolers	**TV time** (hr/day) assessed by parent-report questionnaire (unpublished).	**Bullying** (categories: bullies, nonbullies) assessed by parent-report questionnaire (unpublished).	**Bullies** had significantly greater **TV time** than **non-bullies** at age 4 yr (Bullies, 5.03 hr/day, SD = 3.92; nonbullies, 3.23 hr/day, SD = 3.92; p = 0.004).	N/A

AFQT, Armed Forces Qualification Test; B, unstandardized beta; β, standardized beta; BASC-2, Behavior Assessment System for Children, second edition; BPI, Behavior Problems Index; CD, compact disk; CBCL, Child Behavior Checklist; CI, confidence interval; CTQ, Child Temperament Questionnaire; IDEFICS, Identification and Prevention of Dietary-and Lifestyle-Induced Health Effects in Children and Infants; KINDL, Questionnaire for Measuring Health-Related Quality of Life in Children and Adolescents-Revised Version; LEAPP, Learning Environments' Activity Potential for Preschoolers; MIT-SEA, Modified Infant-Toddler Social and Emotional Assessment; NLSY-Child, National Longitudinal Survey of Youth 1979 Children and Youth Adults; NLSY79, National Longitudinal Survey of Youth; OR, odds ratio; PSID, Panel Study of Income Dynamics; PCTC, Prospective Cohort Study in Thai Children; QLSCD, Quebec Longitudinal Study of Child Development; RCT, Randomized Controlled Trial; SBQ, Social Behavior Questionnaire; SD, standard deviation; SDQ, Strengths and Difficulties Questionnaire; SE, standard error, T1-T4, Time 1 to Time 4; TV, television.

Table S4. Summary of findings for cognitive development outcomes

Reference; Country; Larger Study or Cohort Name	Study Design	Sample	Exposure	Outcome	Main Findings	Covariates included in model (if applicable)
Study Design: Longitudinal						
Tomopoulos et al. 2010 [85]; USA<br><br>Bellevue Project for Early Language, Literacy, and Education Success (BELLE)	Longitudinal (~8 mo follow-up)	N = 259<br><br>Approximate age: T1, 6 mo (~0.5 yr)<br><br>T2, 14 mo (~1.2 yr) Infants	**Electronic media exposure** (min/day) assessed by maternal-report interview (24-hour recall diary).	**Cognitive development** assessed using Bayley Scales of Infant Development-third edition (BSID-III).<br><br>**Language development** (total) and two subscales, **auditory comprehension** and **expressive communication**, assessed using the Preschool Language Scale-4 (PLS-4).	**Electronic media exposure** at age 6 mo was unfavourably associated with **cognitive development** (r = -0.07, p = 0.008; B = -1.5, 95% CI: -2.7, -0.3; β = -0.15, p = 0.02), **language development** (r = -0.16, p = 0.009; B = -1.2, 95% CI: -2.0, -0.4; β = -0.16, p = 0.005), and **auditory comprehension** (r = -0.16, p = 0.01; B = -1.1, 95% CI: -2.0, -0.2; β = -0.14, p = 0.02) at age 14 mo.<br><br>**Electronic media exposure** at age 6 mo was not associated with **expressive communication** at age 14 mo in unadjusted analysis (r = -0.12, p = 0.06) but was unfavourably associated after adjustments (B = -1.0, 95% CI: -1.9, -0.1; β = -0.13, p = 0.02).	Sex, position in birth order, maternal factors (age, educational level, country of origin, primary language, marital status, depressive symptoms, cognitive home environment), intervention status.

Reference; Country; Larger Study or Cohort Name	Study Design	Sample	Exposure	Outcome	Main Findings	Covariates included in model (if applicable)
McKean et al. 2015 [86]; Australia<br><br>Early Language in Victoria Study (ELVS)	Longitudinal (~5 yr follow-up)	N = 763<br><br>Approximate Ages:<br>T1, 8 mo (Infants)<br>T2, 1 yr (Infants)<br>T3, 2 yr (Toddlers)<br>T4, 3 yr (Toddlers)<br>T5, 4 yr (Pre-schoolers)<br>T6, 7 yr	**TV time** (hr/day) at age ~4 yr assessed by parent-report questionnaire (unpublished).<br><br>Average **frequency of parents reading** [scored as: not very often (1), sometimes (2), or often (3)] to their child; assessed at 8 mo, 1, 2, 3 and 4 yr.	**Language development** (SD) assessed by: (1) the Australian adaptation of the Clinical Evaluation of Language Fundamentals-Preschool, Second Edition (CELF-P2) at age 4 yr, (2) the Clinical Evaluation of Language Fundamentals, Fourth Edition (CELF-4) Australian Standardisation at ages 5 and 7 yr.<br><br>**Rate of change in language development** (SD change per year) between ages 4 yr and 7 yr.	There was a dose-response unfavourable relationship between **TV time** at age 4 and the **rate of change in language development** between ages 5-7, such that those with more TV time had a slower rate of change (improvement) in language development; compared to the lowest Quartile:<br>Q2: Coefficient = -0.3, 95% CI: -0.07, 0.01, $p < 0.10$<br>Q3: Coefficient = -0.04, 95% CI: -0.08, 0.10, $p < 0.10$<br>Q4: Coefficient = -0.07, 95% CI: -0.13, -0.03, $p < 0.001$.<br><br>There was a dose-response favourable relationship between **frequency of reading to child from ages 8 mo to 4 yr** and **language development at age 4 yr,** such that children in the Q3 and Q4 of **frequency of parents reading to child from ages 8 mo to 4 yr**, but not those in Q2, had lower **language development**	Gender, birth weight, non-verbal IQ, family history, developmental disorder, shy/approach-withdrawal, language background, social disadvantage index, low income, maternal age, birth position, maternal education, family literacy, conduct score, peer score, pro-social score, emotional score, hyperactivity/inattention, speech development, frequency of reading to child, number of children's books in the home.

Reference; Country; Larger Study or Cohort Name	Study Design	Sample	Exposure	Outcome	Main Findings	Covariates included in model (if applicable)
					than children in Q1 at age 4 yr; compared to Q1 (score 3, "often)):<br>Q2 (score 2.6-2.8): Coefficient = -0.15, 95% CI: -0.30, 0.01, $p < 0.10$<br>Q3 (score >2.2 and <2.6): Coefficient = -0.21, 95% CI: -0.35, -0.06, $p < 0.01$<br>Q4 (score ≤2.2, "sometimes or not very often"): Coefficient = -0.38, 95% CI: -0.56, -0.21, $p < 0.001$<br><br>There was a dose-response favourable relationship between **frequency of reading to child between ages 8 mo to 4 yr** and the **rate of change in language development** between ages 5-7, such that those with a greater frequency of reading to child had a faster rate of change (improvement) in language development; compared to the highest Quartile (Q1):<br>Q2: Coefficient = 0.05, 95% CI: 0.01, 0.10, $p < 0.05$<br>Q3: Coefficient = 0.05, 95% CI: 0.01, 0.10, p <	

Reference; Country; Larger Study or Cohort Name	Study Design	Sample	Exposure	Outcome	Main Findings	Covariates included in model (if applicable)
					0.01<br>Q4: Coefficient = 0.02, 95% CI: -0.03, 0.07, p > 0.05.	
Cheng et al. 2010 [72]; Japan<br><br>Japan Children's Study	Longitudinal (~1 yr follow-up)	N = 302<br><br>Approximate age: T1, 18 mo (~1.5 yr) T2, 30 mo (~2.5 yr)<br>Toddlers	**TV time** (hr/day) at ages ~18 and 30 mo assessed by parent-report questionnaire (unpublished).<br><br>**TV time patterns** from age ~18 mo to age ~30 mo [categories: **high-high**, ≥4 hr/day at 18 and 30 mo; **high-low**, ≥4 hr/day at 18 mo and <4 hr/day at 30 mo; **low-high**, <4 hr/day at 18 mo and ≥4 hr/day at 30 mo; **low-low** <4 hr/day at 18 and 30 mo] assessed by parent-report questionnaire (unpublished).	**Hyperactivity-inattention** (measured on a scale from 0 to 10; higher numbers are unfavourable) assessed by parent-report using the Japanese version of the Strengths and Difficulties Questionnaire (SDQ).	There was an unfavourable dose-response relationship between **TV time** at age ~18 mo and **hyperactivity-inattention** at age ~30 mo (mean scores by TV time categories: *unadjusted*, <1hr/day, 3.26; 95% CI: 2.7, 3.8; ≥1 to <3 hr/day, 3.83, 95% CI: 3.5, 4.2; ≥3 to <4 hr/day, 4.45, 95% CI: 3.9, 4.9; ≥4 hr/day, 4.61, 95% CI: 4.3, 5.0; p-value for mean differences, p < 0.0001; p-value for linear trend for means, p < 0.0001; *adjusted*, <1hr/day, 3.41; 95% CI: 2.7, 4.1; ≥1 to <3 hr/day, 3.81, 95% CI: 3.4, 4.2; ≥3 to <4 hr/day, 4.26, 95% CI: 3.7, 4.8; ≥4 hr/day, 4.59, 95% CI: 4.1, 5.0; p-value for mean differences, p = 0.012; p-value for linear trend, p = 0.002).	Child sex, birth weight, gestational age, birth order, maternal education and family income and maternal stimulation.

Reference; Country; Larger Study or Cohort Name	Study Design	Sample	Exposure	Outcome	Main Findings	Covariates included in model (if applicable)
					Children with **high-high** and **high-low TV time patterns** had more unfavourable **hyperactivity-inattention** scores compared to children with **low-low TV time patterns** (high-high, 4.49, 95% CI: 3.9, 5.1; high-low, 4.70, 95% CI: 4.0, 5.4; low-high, 4.40, 95% CI: 3.5, 5.3; low-low, 3.79, 95% CI: 3.5, 4.1; p-value for mean differences, p = 0.029; p-values for post hoc tests for specific group differences, not reported).	
Mistry et al. 2007 [75]; USA; Healthy Steps for Young Children	Longitudinal (~3 yr follow-up)	N = 2707<br><br>Approximate age:<br>T1: 30 to 33 mo (2.5 to 2.8 yr)<br>Toddlers<br><br>T2: 5.5 yr<br>School-age	**TV time** (hr/day, and categories: >2 hr/day, ≤2 hr/day) assessed by parent-report interview (unpublished format).	**Attention problems** (scale from 0 to 10; higher scores indicate more problems) assessed by parent-report using the Child Behavior Checklist (CBCL).	**TV time** at age ~2.5 yr was not associated with **attention problems** (*unadjusted:* β = 0.14, 95% CI: -0.05, 0.33; *adjusted:* β = -0.07, 95% CI: -0.25, 0.12; both p > 0.05) at age ~5.5 yr.	Maternal demographic characteristics (age at child's birth, race, ethnicity, marital status, employment, education), child's gender, parity, household income, child's health status, maternal depressive symptoms, parental involvement in child's activities, intervention status (note: the intervention is designed to enhance the delivery of developmental services for families).

Reference; Country; Larger Study or Cohort Name	Study Design	Sample	Exposure	Outcome	Main Findings	Covariates included in model (if applicable)
Foster and Watkins 2010 [87]; USA<br><br>National Longitudinal Survey of Youth (NLSY79)<br><br>*NOTE:* This study is a reanalysis of data in Christakis et al. 2004 [88].	Longitudinal (~6 yr follow-up)	N = 946<br><br>Approximate age:<br>T1, 1 yr<br>T2, 3 yr<br>Toddlers<br><br>T3, 7 yr<br>School-age	**TV time** (hr/day) at ages 1 and 3 yr assessed by parent-report interview (unpublished format).	**Attentional problems** (categories: no attentional problems, score <120; attentional problems, score ≥120) assessed by parent-report using the hyperactivity subscale of the Behavior Problems Index (BPI).	**TV time** at age ~1 yr was not associated with **attentional problems** at age ~7 yr (OR = 1.06, SE = 0.05, p > 0.05; compared to 0 hr/day: 0-1 hr/day, OR= 0.80, SE = 0.50; 1-2 hr/day, OR=1.25, SE = 0.52; 2-3 hr/day, OR=1.04, SE = 0.44; 3-4 hr/day, OR=1.82, SE = 0.82; 4-5 hr/day, OR=1.99, SE = 1.03; 5-6 hr/day, OR=0.74, SE = 0.60; 6-7 hr/day, OR= 1.24, SE = 1.18; >7 hr/day, OR=1.58, SE = 0.90; all p > 0.05).<br><br>**TV time** at age ~3 yr was not associated with **attentional problems** at age ~7 yr (OR = 1.05, SE = 0.05, p > 0.05; compared to 0-1 hr/day: 1-2 hr/day, OR=0.39, SE = 0.25; 2-3 hr/day, OR=0.77, SE = 0.43; 3-4 hr/day, OR=0.82, SE = 0.46; 4-5 hr/day, OR=0.49, SE = 0.30; 5-6 hr/day, OR=0.83, SE = 0.57; 6-7 hr/day, OR=3.29, SE = 2.53; >7 hr/day, OR=1.41, SE =	Gender, age, race, gestational age of child, alcohol and tobacco consumption during pregnancy, number of children in the household, presence of two parents, cognitive stimulation and emotional support scores, mother's age, urbanicity, year fixed effects, maternal depression, maternal self-esteem, mother's Armed Forces Qualification Test (AFQT) score, family poverty status.

Reference; Country; Larger Study or Cohort Name	Study Design	Sample	Exposure	Outcome	Main Findings	Covariates included in model (if applicable)
					0.92; all p > 0.05).	
Pagani et al. 2013 [69]; Canada<br><br>Quebec Longitudinal Study of Child Development (QLSCD)	Longitudinal (~3 yr follow-up)	N = 1999<br><br>Approximate Age:<br>T1, 29 mo (~2.4 yr) Toddlers<br><br>T2, 65 mo (~5.4 yr) School-age	**TV time** (hr/day) at age 29 mo assessed by parent-report questionnaire (unpublished).	**Receptive vocabulary** (SD; higher scores are better) assessed by trained research assistants using the Peabody Picture Vocabulary Test (PPVT) score (French adaptation).<br><br>**Number knowledge (units)** assessed from the Number Knowledge Test (NKT) (abridged version).<br><br>**Classroom engagement** (scores ranging from 1 to 5; higher scores are better) assessed by teacher-report questionnaire [89].	**TV time** at age ~29 mo was unfavourably associated with **receptive vocabulary** (B = -0.22, 95% CI: -0.291, -0.149, p < 0.000) **number knowledge** (B = -0.029, 95% CI: -0.043, -0.015, p < 0.000), and **classroom engagement** (B = -0.002, 95% CI: -0.004, -0.000, p = 0.015) at age ~65 mo.	**Receptive vocabulary:** Not reported.<br><br>**Number knowledge:** Maternal education, early stimulation of literacy, early childhood temperament, family functioning.
Pagani et al. 2010 [9]; Canada<br><br>QLSCD	Longitudinal (~8 yr follow-up)	N = 1314<br><br>Approximate age:<br>T1, 29 mo (~2.4 yr) Toddlers<br><br>T2, 53 mo (~4.4 yr) Pre-schoolers<br><br>T3, Grade 4 (in Grade 4 in Quebec, children are aged 9 to 10; School-age)	**TV time** (hr/week) at age 29 mo assessed by parent-report questionnaire (unpublished).<br><br>**Change in TV time** (hr/week) from age 29 mo to 53 mo.	**Cognitive ability** (no units; higher scores are better) assessed using the Imitation Sorting Task.<br><br>**Classroom engagement** assessed by teacher-report using a Classroom Engagement Scale (11 items representing task orientation, compliance, and persistence, rated on a 3-point Likert scale with higher values indicating a higher degree of the	**TV time** at age 29 months was unfavourably associated with **classroom engagement** in Grade 4 (β = -0.07, SE = 0.003; B = -0.01, 95% CI: -0.02, -0.004; p ≤ 0.05).<br><br>**TV time** at age 29 mo was unfavourably associated with **mathematical success** in Grade 4 (age ~9-10 yr) (standardized regression: β = -0.06, SE = 0.01, p ≤ 0.05) or not	**TV time:** Change in TV time, concurrent TV time, sex, temperament, cognitive ability, impulsivity, emotional distress, physical aggression, *hours of sleep*, maternal education, family makeup, family functioning.<br><br>**Change in TV time:** Same as above, except inclusion of "baseline TV time" instead of "change

Reference; Country; Larger Study or Cohort Name	Study Design	Sample	Exposure	Outcome	Main Findings	Covariates included in model (if applicable)
				factor).<br><br>**Mathematical success** rated as relative to the distribution in the class (scale ranging from -2 to 2, with -2 being "bottom of the class").	(unstandardized regression: B = -0.01, 95% CI: -0.03, 0.01, p>0.05).<br><br>The **change in TV time** from age 29 mo to 53 mo was not associated with **classroom engagement** in Grade 4 (age ~9-10 yr; β = 0.03, SE = 0.002, p > 0.05).<br><br>The **change in TV time** from age 29 mo to 53 mo was not associated with **mathematical success** in Grade 4 (age ~9-10 yr; β = 0.04, SE = 0.004, p > 0.05).	in TV time".
Schmidt et al. 2009 [6]; USA<br><br>Project Viva	Longitudinal (~2.5 yr follow-up)	N = 872<br><br>Approximate age:<br>T1, 6 mo (~0.5 yr) to 2 yr<br>Toddlers to Pre-schoolers<br><br>T2, 3 yr<br>Pre-schoolers	**TV time** (hr/day) assessed by parent-report questionnaire [adapted from questionnaire used in the National Longitudinal Survey of Children and Youth study (NLSY)] at ages 6 mo, 1 yr and 2 yr, and as the weighted average of TV exposure from birth and 2 yr.	**Receptive vocabulary** (units not indicated; higher scores are better) assessed by trained research assistants using the Peabody Picture Vocabulary Test III score (PPVT-III).	**Receptive vocabulary** scores at age 3 differed by TV time from birth to 2 yr of age (mean PPVT-III score by TV time: 0 to 0.5 hr/day, mean = 106.2, SD = 14.1; 0.5 to < 1 hr/day, mean = 103.1, SD = 14.1; 1 to <2 hr/day, mean = 105.7, SD = 14.0; ≥2 hr/day, mean = 102.6, SD = 14.4; p = 0.03; post hoc tests not performed to identify specific	**TV time from age birth to 2 yr:** Age, gender, maternal age, education, marital status, parity, household income, child birth weight for gestational age z score, breastfeeding duration, race/ethnicity, English language, *average daily sleep duration from 6 mo to 2 yr*.<br><br>**TV time at ages 6 mo, 1**

Reference; Country; Larger Study or Cohort Name	Study Design	Sample	Exposure	Outcome	Main Findings	Covariates included in model (if applicable)
					differences).<br><br>**TV time** from birth to 2 yr was not associated with **receptive vocabulary** at age 3 yr (B = 0.58, 95% CI: -0.45, 1.61).<br><br>**TV time** at 6 mo, 1 yr and 2 yr of age was not associated with **receptive vocabulary** at age 3 yr (6 mo: B = 0.43, 95% CI: -0.32, 1.18; 1 yr: B = 0.24, 95% CI: -0.37, 0.85; 2yr: B = 0.59, 95% CI: -0.28, 1.46).	**yr and 2 yr:** Maternal age, education, marital status, parity, household income, child birth weight for gestational age z score, breastfeeding duration, race/ethnicity, English language, *average daily sleep duration*.
Zimmerman and Christakis 2005 [90]; USA<br><br>National Longitudinal Survey of Youth 1979 Children and Young Adults (NLSY-Child)	Longitudinal (~4 yr follow-up)	N = 1797<br><br>Age at baseline: “younger than 3 yr” (Toddlers) and “age 3 to 5 yr” (Pre-schoolers)	**TV time** (hr/day) assessed by parent-report questionnaire (unpublished) before age 3 yr and between ages 3 to 5 yr.<br><br>Children were divided into **TV time categories** according to their viewing histories at baseline and follow-up:<br>(1) "**low-low TV**": <3 hr/day before age 3 yr and at age 3-5 yr;<br>(2) "**low-high TV**": <3 hr/day before age 3 yr and > 3hr/day at age 3-5 yr;	**Mathematics**, **reading recognition**, and **reading comprehension** Performance (all age-standardized scores with no units) measured by the Peabody Individual Achievement Test (PIAT).<br><br>**Short-term memory** (age-standardized score; no units) measured by the Memory for Digit Span assessment from the Wechsler Intelligence Scale for Children (WISC).	**TV time** at <3 yr (B = -0.17, 95% CI: -0.50, 0.16) and 3-5 yr (B = -0.01, 95% CI: -0.41, 0.39) was not associated with **Mathematics** at age 6 yr.<br><br>Compared to **high-high TV** (reference group), **Mathematics** at age 6 yr was more favourable in the **low-high TV** (B = 2.74, 95% CI: 0.89, 4.60) and **low-low TV** (B = 2.03, 95% CI: 0.24, 3.83) groups, but was not different in the **high-low TV** group (B = 1.99, 95%	TV time at age 6 yr; parental cognitive stimulation at ages 0 to 3 yr, 3 to 5 yr and at 6 yr; non-English native language; race/ethnicity; mother's education and intelligent quotient (IQ).

Reference; Country; Larger Study or Cohort Name	Study Design	Sample	Exposure	Outcome	Main Findings	Covariates included in model (if applicable)
			(3) "**high-low TV**": >3 hr/day before age <3 yr and <3hr/day at age 3-5 yr;<br>(4) "**high-high TV**": >3hr/day at age <3 yr and at age 3-5 yr.		CI: -0.97, 4.96).<br>**Mathematics** in the **low-low TV** and **low-high TV** groups were not different from each other.<br><br>**TV time** at <3 yr was unfavourably associated with **reading recognition** at age 6 yr (B = -0.31, 95% CI: -0.61, -0.01).<br>**TV time** at 3-5 yr was favourably associated with **reading recognition** at age 6 yr (B = 0.51, 95% CI: 0.17, 0.85).<br><br>Compared to **high-high TV** (reference group), **reading recognition** at age 6 yr was more favourable in the **low-high TV** (B = 1.85, 95% CI: 0.15, 3.55), but not the **low-low TV** (B = 0.01, 95% CI: -1.74, 1.75) or **high-low TV** groups (B = -0.91, 95% CI: -3.56, 1.74).<br><br>**TV time** at <3 yr was unfavourably associated with **reading comprehension** at age 6 yr (B = -0.58, 95% CI: -	

Reference; Country; Larger Study or Cohort Name	Study Design	Sample	Exposure	Outcome	Main Findings	Covariates included in model (if applicable)
					0.94, -0.21), but **TV time** at ages 3-5 yr was not (B = 0.28, 95% CI: -0.12, 0.68).<br><br>Compared to **high-high TV** (reference group), **reading comprehension** at age 6 yr was more favourable in the **low-high TV** (B = 3.92, 95% CI: 1.89, 5.95) and **low-low TV** (B = 2.32, 95% CI: 0.43, 4.22) but not the **high-low TV** (B = 1.66, 95% CI: -1.82, 5.13) groups.<br><br>**TV time** at <3 yr was not associated with **short-term memory** at age 7 yr (B = -0.10, 95% CI: -0.20, 0). **TV time** at ages 3-5 yr was not associated with **short-term memory** at age 7 yr (B = 0.09, 95% CI: -0.04, 0.22).<br><br>Compared to **high-high TV** (reference group), **short-term memory** at age 7 yr was more favourable in the **low-low TV** group (B = 0.59, 95% CI: 0.03, 1.15), but not the	

Reference; Country; Larger Study or Cohort Name	Study Design	Sample	Exposure	Outcome	Main Findings	Covariates included in model (if applicable)
					**low-high TV** (B = 0.52, 95% CI: -0.07, 1.12) or **high-low TV** (B = 0.30, 95% CI: -0.67, 1.27) groups.	
Christakis et al. 2004 [88]; USA<br><br>NLSY-Child (1986-2000)	Longitudinal (~6 yr follow-up)	N = 1345<br><br>Mean age:<br>T1, 1.8 yr<br>Toddlers<br><br>T2, 3.8 yr<br>Pre-schoolers<br><br>T3, ~7 yr<br>School-age	**TV time** (hr/day) assessed by parent-report questionnaire (unpublished).	**Attentional problems** ("present" if score ≥1.2 SDs above the mean age-specific standardized score) assessed using the hyperactivity subscale of the Behavioral Problems Index (BPI).	**TV time** at age ~1 yr was unfavourably associated with **attentional problems** at age ~7 yr (OR = 1.09, 95% CI: 1.03, 1.15). A 1 SD increase in **TV hr/day** at age ~1 yr was associated with a 28% increase in the probability of **attentional problems** at age ~7 yr.<br><br>**TV time** at age ~3 yr was unfavourably associated with **attentional problems** at age ~7 yr (OR = 1.09, 95% CI: 1.02, 1.16).	Gender; race/ethnicity; child age at index interview; gestational age at birth; factors at or near ages 1 and 3: measures of cognitive stimulation and emotional support in the home environment, number of children in the household, presence of 2 parents in the household ; maternal factors: use of alcohol or tobacco during pregnancy, self-esteem as of 1987, age at index, l education at index; and calendar year at index.
Blankson et al. 2015 [91]; USA	Longitudinal (~0.5 and 1.5 yr follow-up)	N = 228<br><br>Approximate Ages:<br>T1, 3.5 yr<br>T2, 4 yr<br>Pre-schoolers<br><br>T3, 5 yr<br>School-age	**TV time** (hr/week) at ages ~3.5 and ~4 yr assessed by parent-report questionnaire ["Watching Television, Reading, and Computers at Home measure" (TVRC); adapted from items by [92].	**Vocabulary** (scores ranging from 0 to 204; higher scores are better) assessed by trained examiner using the PPVT-III.<br><br>**Cognitive inhibitory control** (scores ranging from 0-16; higher scores	In unadjusted analyses, **TV time** at age ~3.5 was unfavourably associated with **vocabulary** (*r* = -0.28, p < 0.01), **working memory capacity** (*r* = -0.18, p<0.01), and **executive function** (*r* = -0.19, p < 0.01), but not with **cognitive inhibitory**	Socioeconomic status (SES), non-European American, home learning environment, parental scaffolding.

Reference; Country; Larger Study or Cohort Name	Study Design	Sample	Exposure	Outcome	Main Findings	Covariates included in model (if applicable)
				indicate stronger inhibitory control) assessed using the Animal Stroop Task.<br><br>**Working Memory Capacity** (no units indicated; higher scores are better) assessed using the Animal Stroop Task (STROOP) and the Kaufman Assessment Battery for Children (K-ABC; [93]) number recall test.<br><br>**Executive function** (standardized scores from two sub-tests; higher scores are better) assessed as a composite of **cognitive inhibitory control** and **working memory capacity**.	**control** (*r* = -0.12, p > 0.05) at age ~5 yr.<br><br>In unadjusted analyses, **TV time** at age ~4 yr was unfavourably associated with **vocabulary** (*r* = -0.20, p < 0.01) and **executive function** (*r* = -0.13, p < 0.05), but not with its components, **cognitive inhibitory control** (*r* = -0.11, p > 0.05) and **working memory capacity** (*r* = -0.10, p > 0.05) at age ~5 yr.<br><br>After adjusting for covariates, **TV time** at age ~3.5 yr was not associated with **vocabulary** (β = -0.13; B = -0.25, SE = 0.13), **cognitive inhibitory control** (β = -0.08; B = -0.04, SE = 0.04), **working memory capacity** (β = -0.11; B = -0.03, SE = 0.02), or **executive function** (β = -0.12; B = -0.03, SE = 0.02) (all p > 0.05).<br><br>After adjusting for covariates, **TV time** at age	

Reference; Country; Larger Study or Cohort Name	Study Design	Sample	Exposure	Outcome	Main Findings	Covariates included in model (if applicable)
					~4 yr was not associated with **vocabulary** (β = -0.03; B = -0.06, SE = 0.12), **cognitive inhibitory control** (β = -0.07; B = -0.03, SE = 0.04), **working memory capacity** (β = -0.01; B = -0.004, SE = 0.02), or **executive function** (β = -0.01; B = -0.05, SE = 0.01) (all p > 0.05).	
Study Design:<br>Case-Control						
Chonchaiya et al. 2008 [94];<br>Thailand	Case-Control	N = 166;<br>Cases, n = 56;<br>Controls, n = 110<br><br>Age range: 15-48 mo<br>Mean age:<br>Cases, 2.11 yr<br>Controls, 2.23 yr<br>Toddlers	**TV time** (hr/day) assessed by parent-report interview (interview protocol unpublished).	**Delayed language development** (cases) and **normal language development** (controls).<br><br>**Delayed language development** determined by medical diagnosis, and developmental assessment using Denver-II test by trained developmental pediatricians. **Language developmental level** (years) was determined by the age at 75th percentile of language development milestones, which the child could attain in Denver-II.	Children with **language delay** (cases) had significantly greater **TV time** than those **normal language development** (mean TV time: cases, 3.05 hr/day, SD = 1.90; controls, 1.85 hr/day, SD = 1.18; mean difference between groups = 1.2 hr/day, 95% CI: 0.726, 1.6737; t = 5.0016; df =1 64; SE of difference = 0.240).<br>Compared with ≤2 hr/day **TV time**, children with >2 hr/day **TV time** had increased odds of language delay (OR = 3.94, 95% CI: 2.00, 7.76, p < 0.001).	N/A

Reference; Country; Larger Study or Cohort Name	Study Design	Sample	Exposure	Outcome	Main Findings	Covariates included in model (if applicable)
Study Design: Cross-sectional						
Zimmerman et al. 2007 [95]; USA	Cross-sectional	N = 729; Infants, n = 384; Toddlers, n = 345<br><br>Age range:<br>Infants, 8-16 mo (~0.7 to 1.3 yr)<br>Toddlers, 17-24 mo (~1.4 to 2 yr)	**Reading** (≥ once per day, <once per day) and **storytelling** (≥ once per day, <once per day) assessed by parent-report interview (interview protocol unpublished).	**Language development** (percentile norms) assessed by the short-form Communicative Development Inventory (CDI).	**Reading** with parents at least once per day, compared with less than once per day, was associated with more favourable **language development** scores in infants (B = 7.07, 95% CI: 0.53, 13.60, $p < 0.05$) and toddlers (B = 11.72, 95% CI: 1.86, 21.59, $p < 0.05$).<br><br>**Storytelling** with parents at least once per day, compared with less than once per day, was associated with more favourable **language development** scores in infants (B = 6.47, 95% CI: 0.23, 12.71, $p < 0.05$), but was not associated **with language development scores** in toddlers (B = 7.13, 95% CI: -0.11, 14.37, $p < 0.10$) .	Sex, age, number of siblings, premature birth, premature birth by age interaction, hours per week in daycare, whether both parents are present, maternal and paternal education, parental income, child race/ethnicity, state of birth (Minnesota or Washington).
Ferguson and Donnellan 2014 [96]; USA<br><br>*NOTE:* This study used the same data set as Zimmerman	Cross-sectional	N = 750<br>6 to 16 mo, n = 392<br>17 to 27 mo, n = 358<br><br>Approximate age:<br>6 to 16 mo (0.5 to 1.3 yr)	**Total media exposure** (min/day, and categories: no exposure, any exposure; baby DVDs or movies, adult TV, children's educational TV, children's educational	**Receptive language development** (understanding words; scale from 0 to 89 for those aged 6 to 16 mo, and 0 to 100 for those aged 17 to 27 mo), **expressive**	**No media exposure** was unfavourably associated with **receptive language development** (r = -0.22, $p < 0.01$; β = -0.13, p = 0.001) and **expressive language development** (r	**No media exposure:** Age, gender.<br><br>**Reading** and **storytelling:** age, gender, listening to music with parents, income, maternal

Reference; Country; Larger Study or Cohort Name	Study Design	Sample	Exposure	Outcome	Main Findings	Covariates included in model (if applicable)
et al. 2007 [95].		Infants to Toddlers<br><br>17 to 27 mo (1.4 to 2.3 yr)<br>Toddlers	DVDs or videos, children's noneducational TV, and children's DVDs or videos of movies) assessed by parent-report questionnaire (unpublished).<br><br>**Reading** and **storytelling with parents** (categories: never, once a month, a few times a month, once a week, a few times a week, once a day, more than once a day) assessed by parent-report interview (interview protocol unpublished).	**language development** (using words; scale from 0 to 100), and **total language development** (receptive and expressive language development; higher scores indicate greater language development) assessed using the Communicative Development Inventory (CDI).	= -0.19, p < 0.01; β = -0.12, p = 0.007) in children aged 6 to 16 mo, but was not associated with **total language development** (*r* = -0.08, p > 0.05; β = -0.03, p > 0.05) in children aged 17 to 27 mo.<br><br>**Reading with parents** was favourably associated with **receptive language development** (β = 0.11, p < 0.01) but not **expressive language development** (β = 0.05, p > 0.05) at age 6 to 16 mo, or **total language development** at age 17 to 27 mo (β = 0.08, p > 0.05).<br><br>**Storytelling with parents** was favourably associated with **receptive language development** (β = 0.12, p < 0.01) but not **expressive language development** (β = 0.03, p > 0.05) at age 6 to 16 mo, and was favourably associated with **total language development** at age 17 to 27 mo (β = 0.18, p < 0.01).	education, baby DVDs/video, adult TV, child educational TV, child noneducational TV, race (Caucasian vs ethnic minority).

Reference; Country; Larger Study or Cohort Name	Study Design	Sample	Exposure	Outcome	Main Findings	Covariates included in model (if applicable)
Byeon and Hong 2015 [97]; Korea<br><br>Panel Study on Korean Children (2010)	Cross-sectional	N = 1778<br><br>Age range: 24 to 30 mo (~2 to 2.5 yr)<br>Toddlers	**TV time** (hr/day) assessed by parent-report questionnaire (unpublished).	**Language development** (categorized as "**normal**" or "**delayed**") assessed by the Korean-Ages and Stages Questionnaire (K-ASQ). "Delayed" language development was defined as a score <2 SD below the mean on the communication domain of the K-ASQ.	There was a dose-response relationship; the risk of **delayed language development** increased proportionately with the increase in toddlers' **TV watching time** (all compared to <1hr/day; 1-2 hr/day: RR = 1.43, 95% CI: 0.59, 3.45, p > 0.05; 2-3 hr/day: RR = 2.74, 95% CI: 1.13, 6.65, p < 0.05; >3 hr/day: RR = 3.03, 95% CI: 1.12, 8.21, p < 0.05; p-value for trend, p=0004).	Environmental factors (main fosterer, household income, size of home city), maternal factors (level of education, economic activities, level of satisfaction with marriage, communication pattern with children), paternal factors (level of education, occupation, communication pattern with children), child factors (gender, sociability, hospitalization experience within the past year due to disease or accident).
Lin et al. 2015 [71]; Taiwan	Cross-sectional	N = 150;<br>TV exposure, n = 75;<br>Control, n = 75<br><br>Mean age: 24.8 mo (~2.1 yr)<br>Toddlers	**TV time** (min/day) assessed by parent-report interview.<br><br>Children were divided into categories based on their average TV time: **frequently exposed** (>0 hr/day TV for children <2 yr, and >2 hr/day TV for children ≥2 yr), or **infrequently exposed** (no TV for children <2 yr, and ≤2 hr/day for children ≥2 yr).	**Overall cognitive development** and **language development** [categorized as "**typical**" (score ≥ 85) or "**delayed**" (score <85)] assessed using Bayley Scales of Infant Development-second edition (BSID-II).	Children with **delayed overall cognitive development** spent more **time watching TV** compared to children with **typical overall cognitive development** (129.3 vs 60.7 min/day; t = 3.1, p < 0.01).<br><br>Children who were **frequently exposed** to TV were more likely to have **delayed cognitive development** than those who were **infrequently exposed** (OR = 3.9, 95%	Analyses were multivariate, but covariates were not specified.

Reference; Country; Larger Study or Cohort Name	Study Design	Sample	Exposure	Outcome	Main Findings	Covariates included in model (if applicable)
					CI: 1.4, 5.9).<br>% of sample with **typical** and **delayed cognitive development** in those **frequently vs infrequently exposed** respectively: typical (66.7 vs 86.7%), delayed (33.3 vs 13.3%) (X^2 = 8.4, p < 0.01).<br><br>Children with **delayed language development** spent more **time watching TV** than children with **typical language development** (117.3 vs 53.2 min/day; t = 3.8, p<0.001).<br><br>Children who were **frequently exposed** to TV were more likely to have **delayed language development** than those who were **infrequently exposed** (OR = 3.3, 95% CI: 1.5, 7.3).<br>% of sample with **typical** and **delayed language development** in those **frequently vs infrequently exposed** respectively: typical (50.7 vs 76.0%), delayed (49.3	

Reference; Country; Larger Study or Cohort Name	Study Design	Sample	Exposure	Outcome	Main Findings	Covariates included in model (if applicable)
					vs 24.0%) (X^2 = 10.4, p < 0.01).	
Pagani et al. 2010 [9]; Canada<br><br>QLSCD	Cross-sectional	N = 1314<br><br>Approximate age: 29 mo (~2.4 yr)<br>Toddlers	**TV time** (hr/week) assessed by parent-report questionnaire (unpublished).	**Cognitive ability** (no units; higher scores are better) assessed using the Imitation Sorting Task.	**TV time** at age 29 mo was not associated with **cognitive ability** at age 29 mo (β = -0.02, SE = 0.2, p > 0.05).	N/A
Cheng et al. 2010 [72]; Japan<br><br>Japan Children's Study	Cross-sectional	N = 302<br><br>Approximate age: 30 mo (~2.5 yr)<br>Toddlers	**TV time** (hr/day) assessed by parent-report questionnaire (unpublished).	**Hyperactivity-inattention** (measured on a scale from 0 to 10; higher numbers are unfavourable) assessed by parent-report using the SDQ.	There was an unfavourable dose-response relationship between **TV time** at age and **hyperactivity-inattention** in *unadjusted* but not *adjusted* analyses (scores by TV time categories: *unadjusted*, <1hr/day, 3.31; 95% CI: 2.6, 4.1; ≥1 to <3 hr/day, 3.99, 95% CI: 3.7, 4.3; ≥3 to <4 hr/day, 4.28, 95% CI: 3.8, 4.8; ≥4 hr/day, 4.53, 95% CI: 4.1, 4.9; p-value for group mean differences, p = 0.031; p-value for linear trend for means, p = 0.004; *adjusted*, <1hr/day, 3.64; 95% CI: 2.8, 4.4; ≥1 to <3 hr/day, 3.94, 95% CI: 3.6, 4.3; ≥3 to <4 hr/day, 4.18, 95% CI: 3.5, 4.7; ≥4 hr/day, 4.48, 95% CI: 4.0, 5.0; p-value for group	Child sex, birth weight, gestational age, birth order, maternal education and family income and maternal stimulation.

Reference; Country; Larger Study or Cohort Name	Study Design	Sample	Exposure	Outcome	Main Findings	Covariates included in model (if applicable)
					mean differences, p = 0.224; p-value for linear trend for means, p = 0.071).	
Duch et al. 2013 [98]; USA	Cross-sectional	N = 119<br><br>Mean age: 21.09 mo (~1.8 yr)<br>Toddlers	**Screen time** (hr/day; TV, cellphones, DVDs, computers) assessed by parent-report 24-hour recall questionnaire (unpublished).	**Communication development** (scores range from 25 to 60; scores >36.5 indicate typical development) assessed by the Ages and Stages Questionnaire: A Parent-Completed Child Monitoring System, Third Edition (ASQ3).	**>2 hr/day screen time** was unfavourably associated with **communication development** (unadjusted: β = -1.71, p = 0.03; adjusted: β = -1.65, p = 0.04).	Child's gender, parent education.
Zimmerman et al. 2009 [99]; USA<br><br>The Language Environment Analysis (LENA) Natural Language Study	Cross-sectional	N = 275<br><br>Mean age: 21.2 mo (~1.8 yr)<br>Toddlers	**TV time** (hr/day) measured objectively using LENA (a vest-worn voice recorder that can differentiate foreground TV, adult voices and child voices; worn for 12 hr/day, 1 day/mo, for 6 mo).	**Language capacity** (units not indicated) assessed by a speech language pathologist using the PLS-4.	**TV time** was not associated with **language capacity** (B = -1.4, 95% CI: -3.97, 1.14, p>0.05).	Child's age, gender, race/ethnicity, mother's and father's education, household income, and number of LENA recording sessions, adult word count, adult-child conversational turns.
Ruangdaraganon et al. 2009 [100]; Thailand<br><br>Prospective Cohort Study of Thai Children	Cross-sectional	N = 203<br><br>Approximate age: 2 yr<br>Toddlers	**TV time** (hr/day) assessed by parent-report questionnaire (unpublished).	**Language development** [categorized as "**pass**" or "**delayed**" (if the child failed all items)] assessed using a modified (translated to Thai language) Clinical Linguistic Auditory Milestone Scale (CLAMS).	**TV time** was not associated with **delayed language development** (compared to <2 hr/day, ≥2 hr/day OR = 0.5, 95% CI: 0.2, 1.6, p > 0.05).	Gender, maternal education, monthly family income, number of children in family, number of televisions in households, television in child's bedroom.

Reference; Country; Larger Study or Cohort Name	Study Design	Sample	Exposure	Outcome	Main Findings	Covariates included in model (if applicable)
Rajchanovska and Ivanovska 2015 [101]; Macedonia	Cross-sectional	N = 1607<br><br>Age range: 3 to 5 yr<br>Toddlers to Pre-schoolers	**Computer use** (yes, no) and **mobile phone use** (yes, no) assessed by parent-report questionnaire (unclear what questionnaire).	**Speech disorders** (categories: yes, no) assessed via questionnaires for children's behaviour (Chuturik test and Child Behavior Checklist by Achenbach), conversation with parents, and clinical examination.	The prevalence of **speech disorders** was not different between those who **used a computer** and those who did not [used a computer: speech disorders, n = 239 (35.41%), no speech disorders, n = 436 (64.59%); did not use a computer: speech disorders, n = 366 (39.27%), no speech disorders, n = 566 (60.73%), p = 0.11].<br><br>The prevalence of **speech disorders** was greater in those who **used a mobile phone** compared to those who did not [used a mobile phone: speech disorders, n = 291 (42.05%), no speech disorders, n = 401 (57.95%); did not use a mobile phone: speech disorders, n = 314 (34.32%), no speech disorders, n = 601 (65.68%), p = 0.001].<br><br>**Computer use** was not associated with **speech disorders** (compared with	N/A

Reference; Country; Larger Study or Cohort Name	Study Design	Sample	Exposure	Outcome	Main Findings	Covariates included in model (if applicable)
					no computer use: OR = 0.848, 95% CI: 0.690, 1.041, p = 0.115).<br><br>**Mobile phone use** was unfavourably associated **speech disorders** (compared with no mobile phone use: OR = 1.389, 95% CI: 1.133, 1.702, p = 0.002).	
McKean et al. 2015 [86]; Australia<br><br>Early Language in Victoria Study (ELVS)	Cross-sectional	N = 763<br><br>Approximate age: 4 yr<br>Pre-schoolers	**TV time** (hr/day) assessed by parent-report questionnaire (unpublished).	**Language development** (SD) assessed by the Australian adaptation of the Clinical Evaluation of Language Fundamentals-Preschool, Second Edition (CELF-P2).	**TV time** at age 4 yr was not associated with **language development** scores at age 4 yr; compared to the lowest Quartile (Q1; <2.71 hr/day):<br>Q2 (>2.71 to < 3 hr/day): Coefficient = -0.08, 95% CI: -0.22. 0.06<br>Q3 (>3 to <3.71 hr/day): Coefficient = -0.09, 95% CI: -0.26, 0.06<br>Q4 (>3.71 hr/day): Coefficient = -0.11, 95% CI: -0.29, 0.07	Gender, birth weight, non-verbal IQ, family history, developmental disorder, shy/approach-withdrawal, language background, social disadvantage index, low income, maternal age, birth position, maternal education, family literacy, conduct score, peer score, pro-social score, emotional score, hyperactivity/inattention, speech development, frequency of reading to child, number of children's books in the home.
Irwin et al. 2015 [84]; Canada<br><br>Learning Environments' Activity Potential for Preschoolers	Cross-sectional	N = 216<br><br>Age range: 2.5-5 yr<br>Pre-schoolers	**Total sedentary time** (min/day; collected during the preschool day only; <50 counts/15 sec epoch) measured by accelerometer (Actical).	**Attention span** (units not indicated) assessed by parent-report using the Child Temperament Questionnaire (CTQ).	**Total sedentary time** was not associated with **attention span** (*r* = -0.14, 95% CI: -0.27, 0.00).	N/A

Reference; Country; Larger Study or Cohort Name	Study Design	Sample	Exposure	Outcome	Main Findings	Covariates included in model (if applicable)
(LEAPP) study						
Linebarger et al. 2014 [102]; USA	Cross-sectional	N = 788<br><br>Mean age: 47.22 mo (~3.9 yr)<br>Pre-schoolers	**Reading time** (hr/day; time that the child read or was read to) assessed by 24-hour parent-report time diary (School Readiness Survey of the National Household Education Surveys Program, 2007).	**Executive function** (T-scores; higher scores indicate poorer executive function) assessed using the Behavior Assessment System for Children (BASC-2; parent-report).	**Reading time** was not associated with **executive function** (values not provided).	Birth order, in-home care, center care, language, cumulative risk status (high or low based on these factors: child minority, maternal age at birth, single parent, maternal education, income-to-needs, more than 3 siblings), parenting style, background TV and music, foreground educational and non-educational TV.
Linebarger 2015 [103]; USA	Cross-sectional	N = 788<br><br>Mean age: 47.22 mo (~3.9 yr)<br>Pre-schoolers	**Time playing video games** (hr/day) assessed by parent-report 24-hour recall diary (adapted from the Panel Study of Income Dynamics (PSID) format).	**Hyperactivity** (*T* scores; higher scores are unfavourable) and **attention problems** (*T* scores; higher scores are unfavourable) assessed by parent-report using the Behaviour Assessment System for Children (BASC-2).	**Time playing video games** was not associated with **hyperactivity** (β = 0.06, CI: -0.09, 0.60; B = 1.38; p <0.10) or **attention problems** (β = -0.03, 95% CI: -5.06, 0.31; B = -0.62; p > 0.10).	Birth order, child grade, literacy, demographic risk, responsive parenting, inconsistent parenting.
Miller et al. 2007 [104]; USA	Cross-sectional	N = 170<br><br>Mean age: 4.31 yr<br>Pre-schoolers	**TV time** (hr/day) assessed by parent-report questionnaire in a semi-structured interview (method published by Christakis et al. 2004 [88]).	Parent- and teacher-reported **attention-deficit/hyperactivity disorder (ADHD) symptoms** (over the last 6 months; scale from 0 to 50; lower scores indicate fewer ADHD symptoms)	**TV time** was unfavourably associated with **teacher-reported** (β=0.235, p = 0.002) but not **parent-reported** (β=0.146, p = 0.06) **ADHD symptoms**.	Sex, age, SES.

Reference; Country; Larger Study or Cohort Name	Study Design	Sample	Exposure	Outcome	Main Findings	Covariates included in model (if applicable)
				measured by checklists based on the Diagnostic and Statistical Manual of Mental Disorders-4 (DSM-IV).		
Nathanson and Fries 2014 [105]; USA	Cross-sectional	N = 107<br><br>Mean age: 53.37 mo (~4.4 yr)<br>Pre-schoolers	**TV time** (min/day) assessed by parent-report questionnaire (unpublished).	**Executive function** (no units; standardized composite score) assessed by four tasks (grass/snow, whisper, backward digit span, tower) taken from [106].	**TV time** was unfavourably associated with **executive function** (partial $r = -0.26$, $p < 0.01$).	Child's age, household income, parent education.

ADHD, attention-deficit/hyperactivity disorder; ASQ3, Ages and Stages Questionnaire: A Parent-Completed Child Monitoring System, third edition; B, unstandardized beta; β, standardized beta; BASC-2, Behavior Assessment System for Children, second edition; BELLE, Bellevue Project for Early Language, Literacy, and Education Success; BPI, Behavioral Problems Index; BSID-II and BSID-III, Bayley Scales of Infant Development-second and third edition; CDI, Communicative Development Inventory; CELF-4, Clinical Evaluation of Language Fundamentals, fourth edition; CELF-P2, Clinical Evaluation of Language Fundamentals-Preschool, second edition; CI, confidence interval; CLAMS, Clinical Linguistic Auditory Milestone Scale; CTQ, Child Temperament Questionnaire; DSM-IV, Diagnostic and Statistical Manual of Mental Disorders-4; ELVS, Early Language in Victoria Study; IQ, intelligent quotient; K-ABC, Kaufman Assessment Battery for Children; K-ASQ, Korean-Ages and Stages Questionnaire; LEAPP, Learning Environments' Activity Potential for Preschoolers study; LENA, Language Environment Analysis; NKT, Number Knowledge Test; NLSY, National Longitudinal Survey of Children and Youth study; NLSY-Child, National Longitudinal Survey of Youth 1979 Children and Youth Adults; OR, odds ratio; PIAT, Peabody Individual Achievement Test; PLS-4, Preschool Language Scale-4; PPVT, Peabody Picture Vocabulary Test; PPVT-III, Peabody Picture Vocabulary Test, third edition; Q1-Q4, Quartile 1 to Quartile 4; QLSCD, Quebec Longitudinal Study of Child Development; RR, relative risk; SD, standard deviation; SE, standard error; SES, socioeconomic status; STROOP, Animal Stroop Task; T1-T4, Time 1 to Time 4; TV, television; TVRC, Watching Television, Reading, and Computers at Home measure; WISC, Wechsler Intelligence Scale for Children.

Table S5. Summary of findings for bone and skeletal health outcomes

Reference; Country; Larger Study/Cohort Name	Study Design	Sample	Exposure	Outcome	Main Findings	Covariates included in model (if applicable)
Study Design: Cross-sectional						
Herrmann et al. 2015 [107]; Sweden, Germany, Hungary, Italy, Cyprus, Spain, Belgium, Estonia<br><br>Identification and prevention of dietary- and lifestyle-induced health effects in children and infants (IDEFICS)	Cross-sectional	N = 1512<br><br>Mean age: 4.4 yr<br>Pre-schoolers	**Total sedentary time** (hr/day; ≤100 cpm, 60 sec epochs) measured by accelerometer (Actigraph uniaxial, ActiTrainer or GT1M).<br><br>**Screen time** (hr/week; TV, videos, DVDs, computer, game console) assessed by parent-report questionnaire (unpublished).	**Bone stiffness index** (no units) measured on the left and right calcaneus using quantitative ultrasound (Achilles Lunar Insight™ GE Healthcare)	After adjusting for moderate-to-vigorous-intensity physical activity (MVPA), accelerometer-derived **sedentary time** was not significantly associated with **bone stiffness index** (β = -0.37; R^2 (%) = 19; p = 0.28).<br><br>There was no association between **screen time** and **bone stiffness index** (β = -0.04; R^2 (%) = 18.4; p = 0.50).	**Total sedentary time:** age, sex, country, FFM, consumption of milk and dairy products, daylight duration, *MVPA*<br><br>**Screen time:** age, sex, country, FFM, consumption of milk and dairy products, daylight duration, leisure time PA

β, standardized beta; FFM, fat free mass; IDEFICS, Identification and prevention of dietary- and lifestyle-induced health effects in children and infants; MVPA, moderate-to-vigorous-intensity physical activity; PA, physical activity; R^2, correlation coefficient; TV, television.

Table S6. Summary of findings for cardiometabolic health outcomes

Reference; Country; Larger Study/Cohort Name	Study Design	Sample	Exposure	Outcome	Main Findings	Covariates included in model (if applicable)
Crispim et al. 2014 [108]; Brazil	Cross-sectional	N = 276<br><br>Mean age: 3.1 yr<br>Pre-schoolers	**TV time** (hr/day) assessed by parent-report questionnaire (unpublished)	**Blood pressure** (categorized based on National High Blood Pressure Education Program in Pediatrics (2004) criteria as "**normal**": SBP and/or DBP values <90th percentile; or "**high**" SBP and/or DBP values ≥ 95th percentile) measured using the second of two automatic blood pressure monitor measurements in sitting position (OMRON-HEM 705 CP).	Watching **TV** for ≥2 hr/day was not associated with **high blood pressure** (compared to <2 hr/day; PR = 0.9, 95% CI = 0.5, 1.4, p = 0.568).	N/A

CI, confidence interval; DBP, diastolic blood pressure; PR, prevalence ratio; SBP, systolic blood pressure; TV, television.

Table S7. Summary of findings for fitness outcomes

Reference; Country; Larger Study/Cohort Name	Study Design	Sample	Exposure	Outcome	Main Findings	Covariates included in model (if applicable)
Study Design: Longitudinal						
Fitzpatrick et al. 2012 [8]; Canada<br><br>Quebec Longitudinal Study of Child Development (QLSCD)	Longitudinal (6 yr follow-up)	N = 1314<br><br>Approximate age:<br>T1, 29 mo (~2.4 yr)<br>Toddlers<br><br>T2, 53 mo (~4.4 yr)<br>Pre-schoolers<br><br>Mean age:<br>T3, 97.8 mo (~8.2 yr)<br>School-age	**TV time** (hr/day) at age ~29 mo assessed by parent-report questionnaire (unpublished).<br><br>**Change in TV time** (hr/week) from age ~29 mo to ~53 mo.	**Lower body explosive strength** (cm) assessed via standing long jump.	**Higher TV time** at age ~29 mo was unfavourably associated with **standing long jump performance** at age 97.8 mo (B = -0.361; 95% CI: -0.576, -0.145; p < 0.001).<br><br>A greater increase in **TV time** between age ~29 and ~53 mo was unfavourably associated with **standing long jump performance** at age 97.8 months (B = -0.285; 95% CI: -0.436,-0.134; p < 0.01).	Sex, family income, weight for gestational age, overeating and *weekly participation in physical activity* at 29 mo (T1), age in mo at T3, and weight status at T3.
Pagani et al. 2010 [9]; Canada<br><br>QLSCD	Longitudinal (8 yr follow-up)	N = 1314<br><br>Approximate age:<br>T1, 29 mo (~2.4 yr)<br>Toddlers<br><br>T2, 53 mo (~4.4 yr)<br>Pre-schoolers<br><br>T3, Grade 4 (in Grade 4 in Quebec, children are aged 9 to 10	**TV time** (hr/week) at age ~29 mo assessed by parent-report questionnaire (unpublished)<br><br>**Change in TV time** (hr/week) from age ~29 mo to ~53 mo.	**Physical fitness levels** [scale ranged from -2 (much less) to 2 (much more) relative to other children] assessed via parent-report questionnaire.	**Higher TV time** at age ~29 mo was unfavourably associated with **physical fitness** in Grade 4 (β = -0.09, SE = 0.0004; B = -0.01, 95% CI: -0.002, -0.02; p < 0.01).<br><br>A greater increase in **TV time** between ~29 mo and ~53 mo of age was unfavourably associated with **physical fitness** in the Grade 4 (β = -0.10, SE = 0.0003, p < 0.01).	**TV time***:* Change in TV time, concurrent TV time, sex, temperament, cognitive ability, impulsivity, emotional distress, physical aggression, *hours of sleep*, maternal education, family makeup, family functioning, BMI<br><br>**Change in TV time***:* Same as above, except inclusion of “baseline TV time” instead of “change in TV time”.

		yr)<br>School-age				

CI, confidence interval; B, unstandardized beta; β, standardized beta; BMI, Body Mass Index; QLSCD, Quebec Longitudinal Study of Child Development; SE, standard error; T1-T4, Time 1 to Time 4; TV, television.

References

1 Yilmaz G, Demirli CN, Karacan CD. An intervention to preschool children for reducing screen time: a randomized controlled trial. Child Care Health Dev 2015;41:443-449. 24571538.

2 Sijtsma A, Sauer PJ, Stolk RP, et al. Infant movement opportunities are related to early growth--GECKO Drenthe cohort. Early Hum Dev 2013;89:457-461. 23631950.

3 Gooze RA, Anderson SE, Whitaker RC. Prolonged bottle use and obesity at 5.5 years of age in US children. J Pediatr 2011;159:431-436. 21543085.

4 Kuczmarski RJ, Ogden CL, Grummer-Strawn LM, et al. CDC growth charts: United States. Adv Data 2000;1-27. PM:11183293.

5 Griffiths LJ, Hawkins SS, Cole TJ, et al. Risk factors for rapid weight gain in preschool children: Findings from a UK-wide prospective study. Int J Obes (Lond) 2010;34:624-632.

6 Schmidt ME, Rich M, Rifas-Shiman SL, et al. Television viewing in infancy and child cognition at 3 years of age in a US cohort. Pediatrics 2009;123:e370-e375. 19254972.

7 Fuller-Tyszkiewicz M, Skouteris H, Hardy LL, et al. The associations between TV viewing, food intake, and BMI. A prospective analysis of data from the Longitudinal Study of Australian Children. Appetite 2012;59:945-948. 23000277.

8 Fitzpatrick C, Pagani LS, Barnett TA. Early childhood television viewing predicts explosive leg strength and waist circumference by middle childhood. Int 2012;9:87. 22793018.

9 Pagani LS, Fitzpatrick C, Barnett TA, et al. Prospective associations between early childhood television exposure and academic, psychosocial, and physical well-being by middle childhood. Arch Pediatr Adolesc Med 2010;164:425-431. 20439793.

10 Reilly JJ, Armstrong J, Dorosty AR, et al. Early life risk factors for obesity in childhood: cohort study. BMJ 2005;330:1357. 15908441.

11 Leary SD, Lawlor DA, Davey SG, et al. Behavioural early-life exposures and body composition at age 15 years. Nutr Diabetes 2015;5:e150. 25664839.

12 Flores G, Lin H. Factors predicting overweight in US kindergartners. Am J Clin Nutr 2013;97:1178-1187. 23553169.

13 Olafsdottir S, Berg C, Eiben G, et al. Young children's screen activities, sweet drink consumption and anthropometry: results from a prospective European study. Eur J Clin Nutr 2014;68:223-228. 24253759.

14 Cole TJ, Bellizzi MC, Flegal KM, et al. Establishing a standard definition for child overweight and obesity worldwide: international survey. BMJ 2000;320:1240. http://www.bmj.com/content/320/7244/1240.abstract.

15 Wheaton N, Millar L, Allender S, et al. The stability of weight status through the early to middle childhood years in Australia: a longitudinal study. BMJ Open 2015;5:e006963. 25922101.

16 De C, V, De B, I, Verbestel V, et al. Risk factors for childhood overweight: a 30-month longitudinal study of 3- to 6-year-old children. Public Health Nutr 2014;17:1993-2000. 24172063.

17 Koleilat M, Harrison GG, Whaley S, et al. Preschool enrollment is associated with lower odds of childhood obesity among WIC participants in LA County. Matern Child Health J 2012;16:706-712. 21431308.

18 Kain J, Andrade M. Characteristics of the diet and patterns of physical activity in obese Chilean preschoolers. Nutrition Research 1999;19:203-215.

19 Wen LM, Baur LA, Rissel C, et al. Correlates of body mass index and overweight and obesity of children aged 2 years: findings from the healthy beginnings trial. Obesity (Silver Spring) 2014;22:1723-1730. 24415528.

20 Li MW, Van der Ploeg HP, Kite J, et al. A Validation Study of Assessing Physical Activity and Sedentary Behavior in Children Aged 3 to 5 Years. Pediatr Exerc Sci 2010;22:408-420. http://search.ebscohost.com/login.aspx?direct=true&db=s3h&AN=54565173&site=ehost-live.

21 Johansson E, Hagstromer M, Svensson V, et al. Objectively measured physical activity in two-year-old children-Levels, patterns and correlates. [References]. The International Journal of Behavioral Nutrition and Physical Activity 2015;12 Jan 2015, ArtID 3.

22 Wijtzes AI, Kooijman MN, Kiefte-de Jong JC, et al. Correlates of physical activity in 2-year-old toddlers: the generation R study. J Pediatr 2013;163:791-799. 23523279.

23 Williams HG, Pfeiffer KA, O'Neill JR, et al. Motor skill performance and physical activity in preschool children. Obesity (Silver Spring) 2008;16:1421-1426. 18388895.

24 Chiasson M. Predictors of obesity in a cohort of children enrolled in WIC as infants and retained to 3 years of age. [References]. Journal of Community Health: The Publication for Health Promotion and Disease Prevention 2016;41:127-133.

25 Dennison BA, Erb TA, Jenkins PL. Television viewing and television in bedroom associated with overweight risk among low-income preschool children. Pediatrics 2002;109:1028-1035. 12042539.

26 Twarog JP, Politis MD, Woods EL, et al. Daily television viewing time and associated risk of obesity among U.S. preschool aged children: An analysis of NHANES 2009-2012. Obesity Research and Clinical Practice 2015;9:636-638.

27 Manios Y, Kourlaba G, Kondaki K, et al. Obesity and television watching in preschoolers in Greece: the GENESIS study. Obesity (Silver Spring) 2009;17:2047-2053. 19282823.

28 Asplund KM, Kair LR, Arain YH, et al. Early Childhood Screen Time and Parental Attitudes Toward Child Television Viewing in a Low-Income Latino Population Attending the Special Supplemental Nutrition Program for Women, Infants, and Children. Child 2015;11:590-599. 26390321.

29 Nelson JA, Carpenter K, Chiasson MA. Diet, activity, and overweight among preschool-age children enrolled in the Special Supplemental Nutrition Program for Women, Infants, and Children (WIC). Prev Chronic Dis 2006;3:A49. 16539790.

30 LaRowe TL, Adams AK, Jobe JB, et al. Dietary intakes and physical activity among preschool-aged children living in rural American Indian communities before a family-based healthy lifestyle intervention. J Am Diet Assoc 2010;110:1049-1057. 20630162.

31 Fulton JE, Wang X, Yore MM, et al. Television viewing, computer use, and BMI among U.S. children and adolescents. J Phys Act Health 2009;6 Suppl 1:S28-S35. 19998847.

32 Chen LP, Ziegenfuss JY, Jenkins SM, et al. Pediatric obesity and self-reported health behavior information. Clin Pediatr (Phila) 2011;50:872-875. 21357199.

33 Kourlaba G, Kondaki K, Liarigkovinos T, et al. Factors associated with television viewing time in toddlers and preschoolers in Greece: the GENESIS study. J Public Health (Oxf) 2009;31:222-230. 19224946.

34 Burdette HL, Whitaker RC. A national study of neighborhood safety, outdoor play, television viewing, and obesity in preschool children. Pediatrics 2005;116:657-662. 16140705.

35 Dolinsky DH, Brouwer RJ, Evenson KR, et al. Correlates of sedentary time and physical activity among preschool-aged children. Prev Chronic Dis 2011;8:A131. 22005624.

36 Barlow SE. Expert committee recommendations regarding the prevention, assessment, and treatment of child and adolescent overweight and obesity: summary report. Pediatrics 2007;120 Suppl 4:S164-S192. PM:18055651.

37 Minh Do LM, Tran TK, Eriksson B, et al. Preschool overweight and obesity in urban and rural Vietnam: differences in prevalence and associated factors. Glob Health Action 2015;8:28615. 26452338.

38 Hajian-Tilaki K, Heidari B. Childhood Obesity, Overweight, Socio-Demographic and Life Style Determinants among Preschool Children in Babol, Northern Iran. Iran J Public Health 2013;42:1283-1291. 26171341.

39 Mendoza JA, Zimmerman FJ, Christakis DA. Television viewing, computer use, obesity, and adiposity in US preschool children. Int 2007;4:44. 17894878.

40 Sijtsma A, Koller M, Sauer PJ, et al. Television, sleep, outdoor play and BMI in young children: the GECKO Drenthe cohort. Eur J Pediatr 2015;174:631-639. 25367053.

41 Proctor MH, Moore LL, Gao D, et al. Television viewing and change in body fat from preschool to early adolescence: The Framingham Children's Study. Int J Obes Relat Metab Disord 2003;27:827-833. 12821969.

42 Byun W, Liu J, Pate RR. Association between objectively measured sedentary behavior and body mass index in preschool children. Int J Obes (Lond) 2013;37:961-965. 23318716.

43 Pate RR, Almeida MJ, McIver KL, et al. Validation and Calibration of an Accelerometer in Preschool Children. Obesity (Silver Spring) 2006;14:2000-2006. http://dx.doi.org/10.1038/oby.2006.234.

44 VAN CAUWENBERGHE EVEL, LABARQUE VALE, Trost SG, et al. Calibration and comparison of accelerometer cut points in preschool children. Int J Pediatr Obes 2011;6:e582-e589. http://dx.doi.org/10.3109/17477166.2010.526223.

45 Brown JE, Broom DH, Nicholson JM, et al. Do working mothers raise couch potato kids? Maternal employment and children's lifestyle behaviours and weight in early childhood. Soc Sci Med 2010;70:1816-1824. 20299142.

46 Taverno RS, Dowda M, Saunders R, et al. Double dose: the cumulative effect of TV viewing at home and in preschool on children's activity patterns and weight status.[Erratum appears in Pediatr Exerc Sci. 2014 Feb;26(1):120]. Pediatr Exerc Sci 2013;25:262-272. 23502043.

47 Turer CB, Stroo M, Brouwer RJ, et al. Do high-risk preschoolers or overweight mothers meet AAP-recommended behavioral goals for reducing obesity? Acad Pediatr 2013;13:243-250. 23491583.

48 Collings PJ, Brage S, Ridgway CL, et al. Physical activity intensity, sedentary time, and body composition in preschoolers. Am J Clin Nutr 2013;97:1020-1028. 23553158.

49 Espana-Romero V, Mitchell JA, Dowda M, et al. Objectively measured sedentary time, physical activity and markers of body fat in preschool children. Pediatr Exerc Sci 2013;25:154-163. 23406703.

50 Lioret S, Maire B, Volatier JL, et al. Child overweight in France and its relationship with physical activity, sedentary behaviour and socioeconomic status. Eur J Clin Nutr 2007;61:509-516. 16988644.

51 Koubaa AA, Younes K, Gabsi Z, et al. [Risk factors of children overweight and obesity]. [French]. Tunis Med 2012;90:387-393. 22585646.

52 Bonvin A, Barral J, Kakebeeke TH, et al. Weight status and gender-related differences in motor skills and in child care - based physical activity in young children. BMC Pediatr 2012;12:23. 22405468.

53 van Stralen MM, te Velde SJ, van NF, et al. Weight status of European preschool children and associations with family demographics and energy balance-related behaviours: a pooled analysis of six European studies. [Review]. Obes Rev 2012;13 Suppl 1:29-41. 22309063.

54 Byun W, Dowda M, Pate RR. Correlates of objectively measured sedentary behavior in US preschool children. Pediatrics 2011;128:937-945. 22007010.

55 Tremblay L, Rinaldi CM. The prediction of preschool children's weight from family environment factors: gender-linked differences. Eat 2010;11:266-275. 20850062.

56 Sasaki A, Yorifuji T, Iwase T, et al. Is There Any Association between TV Viewing and Obesity in Preschool Children in Japan? Acta Med Okayama 2010;64:137-142. 20424669.

57 Anderson SE, Whitaker RC. Household routines and obesity in US preschool-aged children. Pediatrics 2010;125:420-428. 20142280.

58 Dubois L, Farmer A, Girard M, et al. Social factors and television use during meals and snacks is associated with higher BMI among pre-school children. Public Health Nutr 2008;11:1267-1279. 18547454.

59 Jouret B, Ahluwalia N, Cristini C, et al. Factors associated with overweight in preschool-age children in southwestern France. Am J Clin Nutr 2007;85:1643-1649. 17556704.

60 Jiang J, Rosenqvist U, Wang H, et al. Risk factors for overweight in 2- to 6-year-old children in Beijing, China. Int J Pediatr Obes 2006;1:103-108. 17907322.

61 Jago R, Baranowski T, Baranowski JC, et al. BMI from 3-6 y of age is predicted by TV viewing and physical activity, not diet. Int J Obes (Lond) 2005;29:557-564. 15889113.

62 Levin S, Martin MW, Riner WF. TV viewing habits and body mass index among South Carolina Head Start children. Ethn Dis 2004;14:336-339. 15328934.

63 DuRant RH, Baranowski T, Johnson M, et al. The relationship among television watching, physical activity, and body composition of young children. Pediatrics 1994;94:449-455. 7936851.

64 Cardon G, De B, I, Iotova V, et al. Health related behaviours in normal weight and overweight preschoolers of a large pan-european sample: The toybox-study. PLoS ONE 2016;11.

65 Harrison K, Liechty JM. Us preschoolers' media exposure and dietary habits: The primacy of television and the limits of parental mediation. [References]. Journal of Children and Media 2012;6:18-36.

66 Vandebosch H, Cleemput KV. Television viewing and obesity among pre-school children: The role of parents. Communications 2007;32:417-446.

67 Roelants M, Hauspie R. Groeicurven Vlaanderen 2004 [Flemish Growth Curves]. 2006. Available: http://www.vub.ac.be/groeicurven/.

68 Hesketh KD, Crawford DA, Abbott G, et al. Prevalence and stability of active play, restricted movement and television viewing in infants. [References]. Early Child Development and Care 2015;185:883-894.

69 Pagani LS, Fitzpatrick C, Barnett TA. Early childhood television viewing and kindergarten entry readiness. Pediatr Res 2013;74:350-355. 23788060.

70 De KA, Peersman W, Onderbeke K, et al. New reference values must be established for the Alberta Infant Motor Scales for accurate identification of infants at risk for motor developmental delay in Flanders. Child Care Health Dev 2013;39:260-267. 22676145.

71 Lin LY, Cherng RJ, Chen YJ, et al. Effects of television exposure on developmental skills among young children. Infant behav 2015;38:20-26. 25544743.

72 Cheng S, Maeda T, Yoichi S, et al. Early television exposure and children's behavioral and social outcomes at age 30 months. J Epidemiol 2010;20 Suppl 2:S482-S489. 20179364.

73 Verlinden M, Tiemeier H, Hudziak JJ, et al. Television viewing and externalizing problems in preschool children: the Generation R Study. Arch Pediatr Adolesc Med 2012;166:919-925. 22869354.

74 Watt E, Fitzpatrick C, Derevensky JL, et al. Too Much Television? Prospective Associations Between Early Childhood Televiewing and Later Self-reports of Victimization by Sixth Grade Classmates. J Dev Behav Pediatr 2015;36:426-433. 26075581.

75 Mistry KB, Minkovitz CS, Strobino DM, et al. Children's television exposure and behavioral and social outcomes at 5.5 years: does timing of exposure matter? Pediatrics 2007;120:762-769. 17908763.

76 Verlinden M, Tiemeier H, Veenstra R, et al. Television viewing through ages 2-5 years and bullying involvement in early elementary school. BMC Public Health 2014;14:157. 24520886.

77 Zimmerman FJ, Glew GM, Christakis DA, et al. Early cognitive stimulation, emotional support, and television watching as predictors of subsequent bullying among grade-school children. Arch Pediatr Adolesc Med 2005;159:384-388. 15809395.

78 Hinkley T, Verbestel V, Ahrens W, et al. Early childhood electronic media use as a predictor of poorer well-being: a prospective cohort study. Jama, Pediatr 2014;168:485-492. 24639016.

79 Rideout V, Roberts DF, Foehr UG. GenerationM: media in the lives of 8-18 year olds. Kaiser Family Foundation; 2005. Available: http://www.outdoorfoundation.org/pdf/ExecutiveSummaryGenerationM (accessed 2013 Sep 25).

80 Intusoma U, Mo-Suwan L, Ruangdaraganon N, et al. Effect of television viewing on social-emotional competence of young Thai children. Infant behav 2013;36:679-685. 23948636.

81 Manganello JA, Taylor CA. Television exposure as a risk factor for aggressive behavior among 3-year-old children. Arch Pediatr Adolesc Med 2009;163:1037-1045. 19884595.

82 Teramoto S, Soeda A, Hayashi Y, et al. Problematic behaviours of 3-year-old children in Japan: Relationship with socioeconomic and family backgrounds. Early Hum Dev 2005;81:563-569.

83 Miller LE, Grabell A, Thomas A, et al. The associations between community violence, television violence, intimate partner violence, parent-child aggression, and aggression in sibling relationships of a sample of preschoolers. [References]. Psychology of Violence 2012;2:165-178.

84 Irwin JD, Johnson AM, Vanderloo LM, et al. Temperament and Objectively Measured Physical Activity and Sedentary Time among Canadian Preschoolers. Prev Med Rep 2015;2:598-601. 26844125.

85 Tomopoulos S, Dreyer BP, Berkule S, et al. Infant media exposure and toddler development. Arch Pediatr Adolesc Med 2010;164:1105-1111. 21135338.

86 McKean C, Mensah FK, Eadie P, et al. Levers for language growth: Characteristics and predictors of language trajectories between 4 and 7 years. PLoS ONE 2015;10.

87 Foster EM, Watkins S. The value of reanalysis: TV viewing and attention problems. Child Dev 2010;81:368-375. 20331673.

88 Christakis DA, Zimmerman FJ, DiGiuseppe DL, et al. Early television exposure and subsequent attentional problems in children. Pediatrics 2004;113:708-713. 15060216.

89 Pagani LS, Fitzpatrick C, Archambault I, et al. School readiness and later achievement: a French Canadian replication and extension. Dev Psychol 2010;46:984-994. PM:20822217.

90 Zimmerman FJ, Christakis DA. Children's television viewing and cognitive outcomes: a longitudinal analysis of national data. Arch Pediatr Adolesc Med 2005;159:619-625. 15996993.

91 Blankson A. Do hours spent viewing television at ages 3 and 4 predict vocabulary and executive functioning at age 5? [References]. Merrill-Palmer Quarterly 2015;61:264-289.

92 Griffin E, Morrison FJ. The unique contribution of home literacy environment to differences in early literacy skills. Early Child Development and Care 1997;127-128:233-243.

93 Kaufman A.S., Kaufman NL. K-ABC: Kaufman assessment battery for children. Circle Pines, MN: American Guidance Service; 1983.

94 Chonchaiya W, Pruksananonda C. Television viewing associates with delayed language development. Acta Paediatr 2008;97:977-982. 18460044.

95 Zimmerman FJ, Christakis DA, Meltzoff AN. Associations between media viewing and language development in children under age 2 years. J Pediatr 2007;151:364-368. 17889070.

96 Ferguson CJ, Donnellan M. Is the association between children's baby video viewing and poor language development robust? A reanalysis of Zimmerman, Christakis, and Meltzoff (2007). [References]. Dev Psychol 2014;50:129-137.

97 Byeon H, Hong S. Relationship between television viewing and language delay in toddlers: evidence from a Korea national cross-sectional survey. PLoS ONE 2015;10:e0120663. 25785449.

98 Duch H, Fisher EM, Ensari I, et al. Association of screen time use and language development in Hispanic toddlers: a cross-sectional and longitudinal study. Clin Pediatr (Phila) 2013;52:857-865. 23820003.

99 Zimmerman FJ, Gilkerson J, Richards JA, et al. Teaching by listening: the importance of adult-child conversations to language development. Pediatrics 2009;124:342-349. 19564318.

100 Ruangdaraganon N, Chuthapisith J, Mo-Suwan L, et al. Television viewing in Thai infants and toddlers: impacts to language development and parental perceptions. BMC Pediatr 2009;9:34. 19460170.

101 Rajchanovska D, Ivanovska BZ. The impact of demographic and socio-economic conditions on the prevalence of speech disorders in preschool children in Bitola. Srp Arh Celok Lek 2015;143:169-173. 26012126.

102 Linebarger DL, Barr R, Lapierre MA, et al. Associations between parenting, media use, cumulative risk, and children's executive functioning. J Dev Behav Pediatr 2014;35:367-377. 25007059.

103 Linebarger DL. Contextualizing video game play: The moderating effects of cumulative risk and parenting styles on the relations among video game exposure and problem behaviors. [References]. Psychology of Popular Media Culture 2015;4:375-396.

104 Miller CJ, Marks DJ, Miller SR, et al. Brief report: Television viewing and risk for attention problems in preschool children. J Pediatr Psychol 2007;32:448-452. 17012738.

105 Nathanson AI, Fries PT. Television exposure, sleep time, and neuropsychological function among preschoolers. [References]. Media Psychology 2014;17:237-261.

106 Carlson SM. Developmentally Sensitive Measures of Executive Function in Preschool Children. Developmental Neuropsychology 2005;28:595-616. http://dx.doi.org/10.1207/s15326942dn2802_3.

107 Herrmann D, Buck C, Sioen I, et al. Impact of physical activity, sedentary behaviour and muscle strength on bone stiffness in 2-10-year-old children-cross-sectional results from the IDEFICS study. Int 2015;12:112. 26377674.

108 Crispim PA, Peixoto MR, Jardim PC. Risk factors associated with high blood pressure in two-to five-year-old children. Arq Bras Cardiol 2014;102:39-46. 24263779.
